# Causal Assessment of Income Inequality on Self‐Rated Health and All‐Cause Mortality: A Systematic Review and Meta‐Analysis

**DOI:** 10.1111/1468-0009.12689

**Published:** 2024-01-31

**Authors:** MICHAL SHIMONOVICH, MHAIRI CAMPBELL, RACHEL M. THOMSON, PHILIP BROADBENT, VALERIE WELLS, DANIEL KOPASKER, GERRY McCARTNEY, HILARY THOMSON, ANNA PEARCE, S. VITTAL KATIKIREDDI

**Affiliations:** ^1^ MRC/CSO Social and Public Health Sciences Unit, School of Health and Wellbeing University of Glasgow; ^2^ School of Social and Political Sciences University of Glasgow

**Keywords:** causality, income inequality, systematic review

## Abstract

**Policy Points**

- Income is thought to impact a broad range of health outcomes. However, whether income inequality (how unequal the distribution of income is in a population) has an additional impact on health is extensively debated.
- Studies that use multilevel data, which have recently increased in popularity, are necessary to separate the contextual effects of income inequality on health from the effects of individual income on health.
- Our systematic review found only small associations between income inequality and poor self‐rated health and all‐cause mortality. The available evidence does not suggest causality, although it remains methodologically flawed and limited, with very few studies using natural experimental approaches or examining income inequality at the national level.

**Context:**

Whether income inequality has a direct effect on health or is only associated because of the effect of individual income has long been debated. We aimed to understand the association between income inequality and self‐rated health (SRH) and all‐cause mortality (mortality) and assess if these relationships are likely to be causal.

**Methods:**

We searched Medline, ISI Web of Science, Embase, and EconLit (PROSPERO: CRD42021252791) for studies considering income inequality and SRH or mortality using multilevel data and adjusting for individual‐level socioeconomic position. We calculated pooled odds ratios (ORs) for poor SRH and relative risk ratios (RRs) for mortality from random‐effects meta‐analyses. We critically appraised included studies using the Risk of Bias in Nonrandomized Studies – of Interventions tool. We assessed certainty of evidence using the Grading of Recommendations Assessment, Development and Evaluation framework and causality using Bradford Hill (BH) viewpoints.

**Findings:**

The primary meta‐analyses included 2,916,576 participants in 38 cross‐sectional studies assessing SRH and 10,727,470 participants in 14 cohort studies of mortality. Per 0.05‐unit increase in the Gini coefficient, a measure of income inequality, the ORs and RRs (95% confidence intervals) for SRH and mortality were 1.06 (1.03‐1.08) and 1.02 (1.00‐1.04), respectively. A total of 63.2% of SRH and 50.0% of mortality studies were at serious risk of bias (RoB), resulting in very low and low certainty ratings, respectively. For SRH and mortality, we did not identify relevant evidence to assess the specificity or, for SRH only, the experiment BH viewpoints; evidence for strength of association and dose–response gradient was inconclusive because of the high RoB; we found evidence in support of temporality and plausibility.

**Conclusions:**

Increased income inequality is only marginally associated with SRH and mortality, but the current evidence base is too methodologically limited to support a causal relationship. To address the gaps we identified, future research should focus on income inequality measured at the national level and addressing confounding with natural experiment approaches.

There is a general acceptance that the health of individuals is impacted by the characteristics of the society they live in, such as neighborhood safety and welfare policy.^1^, ^2^ Income inequality, a measure of the distribution of income within a population, has been suggested to be a major determinant of population health.^3^, ^4^ Many studies report that countries with high income inequality also report high rates of infant mortality,^5^ cases of COVID‐19,^6^ and unhappiness.^7^

Widening income inequality has been suggested to reduce the health of the whole population across the entire socioeconomic gradient, with psychosocial stress and its adverse consequences impacting the health of everyone.^8^, ^9^, ^10^ In contrast, others argue that economically unequal societies are more likely to have a high proportion of people experiencing low income and poverty and that the relationship between individuals experiencing poverty and health explains the association between income inequality and population health.^3^, ^11^, ^12^ In this sense, individual income is a “cross‐level” confounder, as it affects both income inequality, which is measured across an area, and health, which is measured for individuals.^13^, ^14^ Multilevel data contain both area‐level and individual‐level information and are needed to disentangle the effects of income inequality on health from the effects of individual‐level income and other cross‐level factors such as socioeconomic position (SEP).^15^, ^16^, ^17^, ^18^

The debate over whether policymakers should prioritize reducing poverty or income inequality (or both) has been ongoing since the first study that suggested that income inequality exerts a causal effect on health was published in 1992.^8^, ^9^, ^19^, ^20^ More recently, the debate has centered around the Richard Wilkinson and Kate Pickett 2010 book *The Spirit Level*.^21^ Interest in the possible causal link between income inequality and health^22^, ^23^ remains high^24^, ^25^, ^26^ because it has been suggested that, if causal, it may result in more than a million excess deaths per year across Organisation for Economic Co‐operation and Development countries.^20^ The recent rise of income inequality both in the United States^27^ and the United Kingdom^28^ for the first time in over a decade, and the suspected link between relative income and COVID‐19 outcomes, has bolstered calls for policymakers to renew their focus on income inequality.^29^ Although identifying the evidence needed to understand the causal role income inequality has on health has been a challenge,^30^ the volume of studies on this topic has increased since the last meta‐analyses were published more than a decade ago, as more studies use multilevel data to address confounding.^1^, ^20^, ^31^ A review exploring the evidence to understand the possible causal effects of income inequality on health is therefore needed.

Our aim was to carry out a causal review of income inequality and two important and commonly used indicators of health: self‐rated health (SRH) or all‐cause mortality (mortality).^32^ Death is an outcome that is meaningful and easily understood by many, with mortality considered more methodologically reliable than specific causes of death.^33^, ^34^ SRH provides a holistic measure of health, which is a strong predictor of more severe outcomes (including early mortality) and is thought to capture aspects of health that cannot be identified by clinical assessments.^35^

The aims of this systematic review were to (a) quantify the association of income inequality with individual‐level mortality and SRH, exploring factors that may explain differences among studies, and (b) assess evidence for causality using Bradford Hill (BH) viewpoints, a causal framework that is widely used in systematic reviews in population health research.

## Methods

### Search Strategy and Selection Criteria

A protocol for this systematic review was published^18^ and registered with PROSPERO (registration: CRD42021252791). This review follows the Preferred Reporting Items for Systematic Review and Meta‐Analysis reporting guidelines^36^ (see Appendix 1 for checklist) and recommendations from the *Cochrane Handbook for Systematic Reviews of Interventions*.^37^ A search strategy was developed with the support of an information scientist with terms such as “income,” “income inequality,” “Gini,” “mortality,” and “health” (full strategy in Appendices 2 and 3). We searched for cohort or cross‐sectional studies investigating the relationship between income inequality and mortality or SRH in Medline, ISI Web of Science, Embase, and EconLit published January 1, 1992, to January 1, 2021. Our review was updated to include studies published by June 6, 2022. To be included, studies had to adjust for at least one individual‐level measure of SEP (e.g., individual or household income, education, occupational status) using multilevel data with income inequality measured on an aggregate level. We did not include studies that considered specific causes of mortality or indicators of general health other than SRH (e.g., subjective well‐being). We included studies using any income inequality metric that was measured at the area level; individual‐level measures of inequality (e.g., Yitzhaki index) were excluded.^38^, ^39^, ^40^ We resolved disagreements over screening and critical appraisal by consensus or in consultation with a third author. Screening was guided by the directed acyclic graph in Appendix 4.

In the initial title‐only screening using Endnote, M.S. and P.B. excluded duplicates and clearly irrelevant studies. Two investigators (M.S. and M.C., S.V.K., A.P., R.T., or H.T.) independently screened titles and abstracts in Covidence and then independently screened the retrieved full texts, which appeared to evaluate income inequality and either outcome. Studies were excluded if (a) the majority of the sample was younger than 18 years old, (b) the exposure was not a measure of the distribution of income across an area, (c) the outcome was not mortality or SRH (e.g., subjective well‐being and mental health were not included), (d) multilevel data were not used, (e) individual‐level SEP was not adjusted for, (f) the study was not published in English and a translation could not be obtained, (g) the study was conducted prior to 1992 (based on one of the first studies suggesting a relationship between income inequality and health^19^), or (h) the study was a duplicate. We did not limit studies based on the statistical methods used to account for clustering.^1^ A full list of clarifications and minor deviations from our protocol can be found in Appendix 5.

Data extraction was conducted by one author (M.S.) and checked by a second author (M.C., S.V.K., A.P., H.T., or R.T.). We extracted information including author, publication year, study setting, income inequality indicator (e.g., Gini coefficient), analytical strategy to address clustering of individuals within areas, effect estimates for each outcome, and the functional form of the exposure (e.g., categorical or continuous) and outcome (e.g., dichotomous or ordinal; Appendix 6).

### Critical Appraisal

Each study included in our review was independently appraised by M.S. and a second reviewer (M.C., S.V.K., A.P., and R.T.) using the Risk of Bias in Nonrandomized Studies – of Interventions (ROBINS‐I) tool, as per Cochrane guidance.^41^ In ROBINS‐I, the nonrandomized study being appraised is compared with a hypothetical randomized controlled trial, known as a target trial, in which practical and ethical issues of randomly assigning income inequality are ignored (full critical appraisal guide in Appendix 7).^42^ The key characteristics of an ideal target trial and potential sources of bias are used to assess each study's overall risk of bias (RoB) according to seven domains: confounding, selection of participants into the study, classification of intervention, deviations from intended exposure, missing data, measurement of outcomes, and selection of the reported results.^43^ The following levels of risk are assigned to each domain and then to the study overall: low, moderate, serious, or critical. The study's overall risk is rated on the highest (most severe) domain‐level judgment.^43^, ^44^

As with most areas of population health research, we consider confounding to be one of the most important threats to bias.^45^, ^46^ Most nonrandomized studies will, at best, have a moderate RoB because of confounding, which means that confounding is expected but has been adequately addressed.^47^, ^48^ Studies will be deemed at serious risk of confounding if residual confounding is thought to be likely. In the case of the current review, for example, confounding poses a serious risk when only one indicator of individual‐level SEP was adjusted for (e.g., educational attainment) or the variables adjusted for were unreliably measured.^43^, ^49^ Additional guidance on the meaning of low, moderate, serious, and critical RoB for the other six domains are detailed in Appendix 7.

### Statistical Analysis

#### Data Standardization

We aimed to standardize estimates to produce a pooled odds ratio (OR) or risk ratio (RR) per 0.05‐unit increase in Gini coefficient for worse SRH and mortality, respectively (additional information on our analysis can be found in Appendices 8‐10, ^50^, ^51^, ^52^, ^53^, ^54^, ^55^, ^56^, ^57^, ^58^, ^59^). A 0.05‐unit increase in Gini coefficient is commonly used across population health research^20^, ^60^, ^61^, ^62^, ^63^ to describe a change in the distribution of income across an area^20^, ^64^, ^65^, ^66^, ^67^ that is considered to be meaningful.^68^ For instance, the increase in the Gini coefficient in the United Kingdom from 0.25 in 1979 to 0.34 in 1990^69^ and the 0.05‐unit rise in the United States from 0.36 in 1979 to 0.41 in 1990^60^ are considered large and meaningful.^69^ Hazard ratios and ORs were standardized to RRs based, respectively, on an approach by Zhang and Yu^61^ and Shor and colleagues.^62^ SRH is measured using a single‐item question about health with several responses from best to worst, such as excellent, very good, good, poor, and very poor, and the measure is highly predictive of mortality.^63^ Measures of SRH were often dichotomized and, if necessary, estimates were reverse coded to reflect worse SRH. Based on previous findings,^20^ we expected *I* ^2^, a measure of between‐study variation, to be high (>75%).^70^ Given our expectations around heterogeneity, we conducted random‐effects meta‐analyses using a restricted maximum likelihood approach. Analyses were performed using R Statistical Software^71^ using the *meta* ^72^ and *dosresmeta* ^73^ packages for the primary meta‐analyses, meta‐regressions, subgroup analyses, and sensitivity analyses. Data and code are available in a public, open‐access repository.^74^

#### Selection of Multiple Effect Estimates

The effect estimates included in a meta‐analysis are typically assumed to be independent, as pooling together nonindependent estimates can overestimate their precision when synthesized.^75^, ^76^ Nonindependence commonly results from including multiple effect estimates with overlapping samples from the same study (i.e., double counting).^75^ Multiple estimates can be found in a single report or across multiple reports of the same study population or sample.^77^, ^78^ To avoid double counting participants, it is recommended that reviewers select the most relevant, independent estimate(s) from each unique study.^78^ The approach to selecting estimates might introduce bias, such as if estimates are selected in a way that confirms reviewers’ perceptions or beliefs.^75^, ^76^ We therefore followed the decision rules described in Appendix 5. The estimates from each unique study and corresponding reports selected for our primary meta‐analyses are described in Appendices 9 and 10. These tables note which estimates were included in our sensitivity analyses, exploring if our approach to selecting estimates, as well as other statistical analysis decisions, affects our results.

#### Exploring Heterogeneity

As previously noted, we expected the estimates included in our review to vary considerably. Although it is not necessary for estimates to be comparable to be synthesized, considerable heterogeneity can make it challenging to interpret and apply review findings.^79^, ^80^ Therefore, we followed systematic review guidance from Cochrane that underscores the importance of identifying sources of heterogeneity (known as covariates or potential effect modifiers) through meta‐regressions and subgroups analyses.^70^ We explored suspected sources of heterogeneity: mean or median Gini coefficient in the sample under study; study RoB (moderate, serious, or critical); global regions where the study is set as classified by the World Bank^81^ (North America, Europe and central Asia, east Asia and Pacific, and Latin America and the Caribbean) and those spanning multiple regions; geographical scale, which refers to both the nature of the sample as well as the level at which income inequality was measured (areas defined as within‐country, local [e.g., cities, neighborhoods, districts]; within‐country, regional [e.g., states, provinces, regions, municipalities, prefectures]; and among countries, national [e.g., continents, world regions, countries]); studies set in the United States vs. outside the United States; adjusted variables (individual‐level variables only vs. individual and contextual/area‐level variables, with the latter potentially reflecting residual confounding or overadjustment); time between income inequality measure and outcome (time lag ≤ 6 years vs. time lag > 6 years); mean or median follow‐up duration; and average age of population (average age 60 years old or younger vs. older than 60 years). Meta‐regressions are used to estimate the amount of between‐study variance, which can be explained by the covariate.^82^ The regression coefficient estimated from a meta‐regression is the response to a one‐unit increase in the level of the covariates.^70^ Subgroup analyses differ from meta‐regressions, as stratified analyses were conducted for each level of the covariate.^83^ We explored publication bias using funnel plots and Egger's test for both outcomes.

### Causal Assessment

#### Certainty of Evidence

We considered causality using two complementary^84^ approaches. The first, the Grading of Recommendations Assessment, Development, and Evaluation (GRADE) approach, is considered the gold standard approach used in systematic reviews.^85^ GRADE provides a structured and transparent approach for rating the level of certainty across a body of evidence based on within‐ and between‐study considerations.^86^ The level of certainty is rated as very low, low, moderate, or high and reflects the confidence users have in effect estimates. The body of evidence automatically begins with high certainty and then may be downgraded according to five domains: RoB, inconsistency, indirectness, imprecision, and publication bias.^85^

#### Weight of Evidence

Our second approach to assessing causality was to use the highly regarded^87^, ^88^, ^89^ and widely used^90^ BH viewpoints.^91^ Epidemiologist Sir Austin Bradford Hill proposed nine characteristics to help researchers discern causal from noncausal associations: strength of association, consistency, specificity, temporality, dose–response gradient, plausibility, coherence, experiment, and analogy.^91^ According to Bradford Hill, strength of association refers to a large, but undefined, association between the exposure and outcome that suggests the relationship cannot be explained by a third variable. Consistency means observing similar associations across different settings or population. Specificity refers to evidence of a one‐to‐one relationship. Temporality indicates that the exposure occurred prior to the outcome. In a dose–response relationship, incremental increases (or decreases) of the exposure are observed alongside incremental increases (or decreases) of the outcome. Plausibility indicates credible explanations of the relationship. Coherence means that assumptions about the causal relationship fit with existing theoretical and empirical evidence. An association observed in an experiment is considered the strongest type of causal evidence. Finally, analogous evidence refers to associations between exposures and outcome with a similar underlying mechanism to the relationship under study.

There are currently no agreed‐on guidelines for how BH viewpoints should be used in causal assessment despite being commonly used in systematic reviews across population health. BH viewpoints have often not been applied systematically and transparently,^90^ which is particularly important for a topic as controversial as the relationship between income inequality and health.^3^, ^92^ To improve the use of BH viewpoints, we drew on the principles of process tracing, which is a causal inference method used for case study research, to evaluate the value of evidence for the causal claim.^93^, ^94^ The relative importance of evidence in process tracing is judged by its uniqueness to the hypothesis (if supportive evidence is found, how unlikely it is to be explained by something other than the hypothesis) and its definitiveness (certainty that if contradictory evidence is found, the hypothesis cannot be true).^93^, ^95^ Process tracing classifies evidence into four types according to their combinations of uniqueness and definitiveness, which we applied to each BH viewpoint. In order of strength, the BH viewpoints by process tracing type are as follows: “doubly decisive” (high uniqueness and definitiveness), “smoking gun” (high uniqueness, low definitiveness), “hoop” (low uniqueness, high definitiveness), and “straw in the wind” (low uniqueness, low definitiveness).^96^ We consider analogy and coherence to be straw‐in‐the‐wind viewpoints, as they can neither disconfirm (low uniqueness) nor confirm (low definitiveness) the hypothesis. The classification assigned to each of the BH viewpoints is described in Table 1. A figure summarizing the relative importance of BH viewpoints by type of process tracing test is in Appendix 11.

**Table 1:** Evidence in Support of and Against Each BH Viewpoint Grouped by the Perceived Uniqueness and Definitiveness of Each Viewpoint

BH Viewpoints	Evidence in Support of BH Viewpoint^a^	Evidence Against BH Viewpoint
**Doubly Decisive Viewpoint (High Uniqueness, High Definitiveness)** ^b^		
Experiment	NESs showing adverse effect of increasing income inequality on health given low or moderate RoB.	NESs showing a protective effect of increasing income inequality on health.
**Smoking Gun Viewpoints (High Uniqueness, Low Definitiveness)** ^c^		
Strength of association	Studies showing an adverse effect of income inequality on health in which (a) all measured confounding has been appropriately adjusted (indicated by RoB assessment) or (b) additional tests suggesting residual confounding and/or unmeasured confounding have been adequately addressed (e.g., tests using negative exposures/outcome, measures of the minimum observed association unlikely explained by confounding [e.g., E‐value]^97^).	Evidence that an increase in income inequality is associated with an improvement in health measured on the individual level given (a) or (b); put simply, the inverse of evidence in support of strength of association.
Dose–response gradient	We used studies that calculated category‐specific exposure–outcome relationships to evaluate the overall dose–response gradient. Categorical studies calculating the association at three or more levels of income inequality for each health outcome compared with a reference exposure level provide insight into the shape (e.g., linearity, threshold effect) of the dose–response gradient.^98^ As with the strength of association, the shape of the exposure–outcome relationship is considered in light of evidence of adequate adjustments for confounding (e.g., low/moderate RoB).	Evidence of a beneficial dose–response gradient (health improves as income inequality increase) from studies in which the shape is unlikely to be caused by confounding.
Specificity	As with many relationships in population health research, we did not expect to identify evidence of a one‐to‐one relationship between income inequality and health. Thus, evidence in support of specificity is that which suggests measured confounding is not responsible for nonspecific associations. In other words, evidence that residual confounding does not remain after confounding variables have been adjusted, such as from falsification tests (negative controls).^99^	Failing falsification tests, suggesting that some of the association between income inequality and health can be explained by measured confounding variables even after they have been adjusted for.
Consistency	Consistency is traditionally defined as similar estimates observed in studies covering different settings and populations (especially if bias has been addressed). As considerable heterogeneity is not uncommon in population health research, evidence in support of consistency also includes prespecified plausible explanations for differences in effect sizes (e.g., contextual differences such as geographical scale) supported by meta‐regressions and/or subgroup analyses.	Evidence against the hypothesis for consistency based on meta‐regressions or subgroup analyses (i.e., unexplained statistical heterogeneity). We do not consider statistical heterogeneity on its own as sufficient for consistency to be unmet.
**Hoop Viewpoints (Low Uniqueness, High Definitiveness)** ^d^		
Plausibility	Theoretical explanatory evidence of the mechanism by which income inequality has an adverse effect on health. Empirical evidence exploring the plausibility of the relationship under study (e.g., mediation analysis to look at mechanisms) may strengthen, but is not necessary, for the overall plausibility of the relationship.	Credible evidence that a relationship is not plausible.
Temporality	Evidence that indicates that reverse causation is unlikely would provide support for the temporality viewpoint. This includes evidence that shows that (a) income inequality precedes the health outcome (e.g., quasi‐experimental/experimental studies); (b) analytical approaches to reduce the possible effect of health on income inequality (e.g., longitudinal studies that adjust for prior or baseline health); or (c) a latent effect of income inequality on health in which an incubation period is thought to suggest, though to a somewhat lesser degree than (a) or (b), that the outcome is unlikely to cause the exposure.	Reverse causation is somewhat unlikely, as only studies that adjusted for individual‐level socioeconomic position were eligible, blocking the effect of health prior to income inequality on income inequality. There is also considerable disagreement over the thresholds for a latent effect. As a result, we do not consider an absence of evidence for (b) or (c) as sufficient for temporality to be unmet. Rather, only the inverse of (a) (that health precedes income inequality) would increase our confidence that reverse causation is occurring and result in temporality being unmet.

BH, Bradford Hill; NES, natural experimental study; RoB, risk of bias.

^a^Evidence is highly unique if it does not overlap with other theories and highly definitive if *not* finding it is a strong indication that the hypothesis is untrue (used to confirm and disconfirm hypothesis, respectively).^100^ As finding evidence for or against the BH viewpoints analogy or coherence will not impact the hypothesis (income inequality adversely affects health) either way, neither are considered.

^b^Experiment is the only highly unique and highly definitive viewpoint, as low RoB experimental/quasi‐experimental studies, but often unavailable in population health research, suggesting an adverse/beneficial effect is a strong indication that the hypothesis is true/untrue, respectively.

^c^Smoking gun viewpoints are highly unique but not definitive. For example, understanding statistical heterogeneity (i.e., consistency is met) may help confirm the hypothesis, but the income inequality and health relationship may be causal even if such evidence is not found (i.e., consistency is unmet).

^d^Hoop viewpoints are highly definitive, as no credible explanation of how causality occurs or evidence of reverse causation (i.e., plausibility or temporality are unmet, respectively) disconfirms the hypothesis, but if it is not unique the hypothesis may be untrue even if plausibility or temporality are met.

Having classified the BH viewpoints by process tracing type, we then explored the evidence for each viewpoint to evaluate our hypothesis: income inequality has an adverse effect on mortality or SRH (each outcome evaluated separately). We did not do this for analogy or coherence because they were considered to be “straw‐in‐the‐wind” viewpoints and therefore have limited value. Using the same principles of process tracing types, finding evidence in support of a highly unique BH viewpoint can help confirm the hypothesis, as supportive evidence is unlikely to be observed if the hypothesis is untrue.^93^ Finding evidence against the highly definitive BH viewpoints increases the possibility that the hypothesis is untrue. The possible judgments for the remaining BH viewpoints were as follows: “met” (evidence found in support of a BH viewpoint, suggesting the hypothesis is true), “unmet” (evidence found against a viewpoint, suggesting hypothesis is untrue), “inconclusive” (some evidence “met” and some evidence “unmet”), and “no evidence” (absence of evidence to evaluate the BH viewpoint). Judgments were guided by the meta‐analysis for each outcome, explorations of heterogeneity (e.g., meta‐regression and subgroup analyses), and results of critical appraisal using ROBINS‐I. The evidence in support of and against each of the seven BH viewpoints included in the causal assessment are also explained in Appendix 5.^93^

## Results

### Included Studies

From the 61,164 studies identified in our search, 9,848 duplicate reports were excluded prior to screening (flow chart in Appendix 12). Of the 51,316 records screened, 50,855 were excluded as irrelevant and 27,912 were excluded at the title‐only stage and the remainder at the abstract stage, with an additional 195 excluded because of duplication. We screened 463 full texts (Appendix 13 gives reasons for exclusion) and found 94 eligible studies using multilevel data to evaluate the association between income inequality and mortality (27/94, 28.1%) or SRH (67/94, 71.9%; list of included studies in Appendix 14). We excluded 28 studies with duplicate data on mortality (6/27, 20.0%)^101^, ^102^, ^103^, ^104^, ^105^, ^106^ or SRH (22/67, 29.7%).^107^, ^108^, ^109^, ^110^, ^111^, ^112^, ^113^, ^114^, ^115^, ^116^, ^117^, ^118^, ^119^, ^120^, ^121^, ^122^, ^123^, ^124^, ^125^, ^126^, ^127^, ^128^ Our primary analyses included 2,916,576 individuals in 38 studies considering SRH^129^, ^130^, ^131^, ^132^, ^133^, ^134^, ^135^, ^136^, ^137^, ^138^, ^139^, ^140^, ^141^, ^142^, ^143^, ^144^, ^145^, ^146^, ^147^, ^148^, ^149^, ^150^, ^151^, ^152^, ^153^, ^154^, ^155^, ^156^, ^157^, ^158^, ^159^, ^160^, ^161^, ^162^, ^163^, ^164^, ^165^, ^166^ and 14 studies with 10,727,470 million individuals considering mortality^141^, ^167^, ^168^, ^169^, ^170^, ^171^, ^172^, ^173^, ^174^, ^175^, ^176^, ^177^, ^178^, ^179^ (study characteristics in Appendices 15 and 16, respectively). An additional seven reports considering SRH^180^, ^181^, ^182^, ^183^, ^184^, ^185^, ^186^ and eight considering mortality,^187^, ^188^, ^189^, ^190^, ^191^, ^192^, ^193^, ^194^ including one natural experimental study,^191^ were included in one or more of the subgroup or sensitivity analyses. The natural experimental study exploits a resettlement policy for refugees arriving in Sweden between 1987 and 1994 whose placement in different municipalities and “initial exposure to income inequality is randomly determined conditional on a few key individual characteristics.”^191^ Despite its favorable study design, estimates from the natural experimental study were excluded from the main analysis, as it used a subset of the Swedish census that overlaps, but is smaller than, another report included in the primary meta‐analysis.^171^

Of the studies considering SRH, most used the Gini coefficient to measure income inequality except for two that used the Theil index^129^, ^130^ and one that used median share.^141^ All but six (15.8%) were cross‐sectional.^134^, ^137^, ^140^, ^141^, ^145^, ^166^ All studies considering mortality were cohort studies, with ten using the Gini coefficient^167^, ^169^, ^175^, ^177^, ^178^, ^179^ and the other three using the median share.^141^, ^168^, ^176^, ^187^ Categorical measures of income inequality, using various cutoffs, were used by 12 studies considering SRH (31.6%^137^, ^146^, ^149^, ^151^, ^153^, ^156^, ^157^, ^160^, ^162^, ^164^, ^165^) and five considering mortality (35.7%^172^, ^175^, ^178^). In the SRH studies, income inequality was predominantly measured within countries at the regional (e.g., states; 14/38, 39.5%^134^, ^135^, ^140^, ^142^, ^144^, ^149^, ^151^, ^154^, ^156^, ^159^, ^162^, ^166^) or local (e.g., cities, neighborhoods; 16/38, 42.1%^130^, ^131^, ^136^, ^139^, ^141^, ^146^, ^147^, ^152^, ^153^, ^157^, ^158^, ^160^, ^163^) level, with just eight studies estimating the impact of income inequality across countries and measured at the national level (18.4%^129^, ^132^, ^133^, ^139^, ^145^, ^148^, ^155^, ^161^). One study^166^ measuring income inequality within one country, but on a national level over time, was included in the within‐country regional group. One study^145^ which included a sample from multiple countries measured income inequality across regional (within‐country) areas. Likewise, the mortality studies predominantly considered income inequality on a local (10/14, 71.4%^141^, ^167^, ^170^, ^171^, ^172^, ^173^, ^174^, ^175^, ^176^, ^177^) or regional (28.6%^168^, ^169^, ^178^, ^179^) scale. We did not identify any that considered the relationship across multiple countries.

Among studies considering SRH, few (7/38, 18.4%) had moderate RoB,^139^, ^150^, ^157^, ^159^, ^161^ with the majority being rated as serious (24/38, 63.2%) ^129^, ^130^, ^132^, ^134^, ^136^, ^137^, ^138^, ^139^, ^140^, ^141^, ^143^, ^144^, ^145^, ^146^, ^147^, ^148^, ^151^, ^153^, ^154^, ^155^, ^156^, ^162^, ^163^, ^164^, ^165^ and a few rated as having critical RoB (7/38, 18.4%).^131^, ^133^, ^141^, ^152^, ^153^, ^158^, ^161^ In contrast, half of the studies considering mortality had moderate RoB (7/14, 50.0%^141^, ^168^, ^171^, ^173^, ^175^, ^178^, ^179^, ^187^) and half serious RoB (7/14, 50.0%^167^, ^169^, ^170^, ^172^, ^174^, ^176^, ^177^). Confounding of preexposure variables was the most common source of bias, with bias caused by the selection of participants into the study and bias caused by classification of interventions (e.g., missing or poorly defined information about values of income inequality) also being important issues (see Appendices 17 and 18 for full critical appraisal results). As noted in Appendix 7, studies that adjusted for variables that we considered to be downstream^195^, ^196^ from income inequality were deemed to have critical RoB caused by confounding (e.g., subjective poverty,^161^ perceived control,^133^ and the level of trust in the community^147^). Studies that did not sufficiently address confounding, including those evaluating the effects across multiple countries that did not adjust for area‐level variables (e.g., GDP^139^) or studies that adjusted for variables that we deemed to insufficiently address individual‐level SEP (e.g., only adjusted for age, gender, household income, and family size^141^), were often considered to have serious RoB.

### Meta‐Analysis of the Effects of Income Inequality on SRH and Mortality

**Figure 1:**
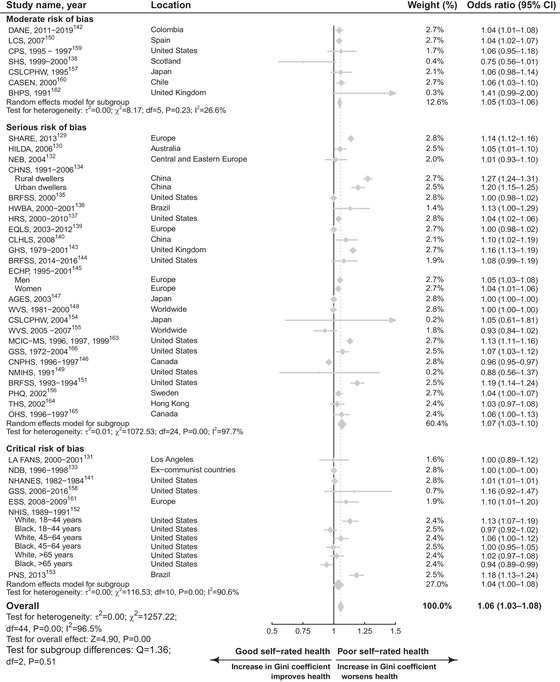
Forest Plot for a Meta‐Analysis of Studies Considering Income Inequality and Poor SRH, Stratified by RoB for a 0.05‐Unit Increase in the Gini Coefficient. The number of people were 2,916,576, and the number of studies were 38. AGES, Aichi Gerontological Evaluation Study Project; BHPS, British Household Panel Survey; BRFSS, Behavioral Risk Factor Surveillance System; CASEN, National Socioeconomic Characterization Survey; CHNS, China Health and Nutrition Survey; CI, confidence interval; CLHLS, Chinese Longitudinal Healthy Longevity Survey; CNPHS, Canadian National Population Health Survey; CPS, Current Population Survey; CSLCPHW, Comprehensive Survey of Living Conditions of People on Health and Welfare; DANE, Departamento Administrativo Nacional de Estadística; ECHP, European Community Household Panel survey; EQLS, European Quality of Life Surveys; ESS, European Social Survey; GHS, British General Household Survey; GSS, General Social Survey; HILDA, Household Income and Labour Dynamics in Australia; HRS, Health and Retirement Study; HWBA, Health, Well‐Being, and Aging study; LA FANS, Los Angeles Family and Neighborhood Survey; LCS, Life Conditions Survey; MCIC‐MS, Metropolitan Chicago Information Center Metro Survey; NDB, New Democracies Barometer; NEB, New European Barometer; NHANES, National Health and Nutrition Examination Survey; NHIS, National Health Interview Survey; NMIHS, National Maternal Infant Health Survey; OHS, Ontario Health Survey; PHQ. Stockholm County Council's Public Health Questionnaire; PNS, National Health Survey; RoB, risk of bias; SHARE, Survey of Health, Ageing and Retirement in Europe; SHS, Scottish household survey; THS, Thematic Household Surveys; WVS, World Values Survey.

**Figure 2:**
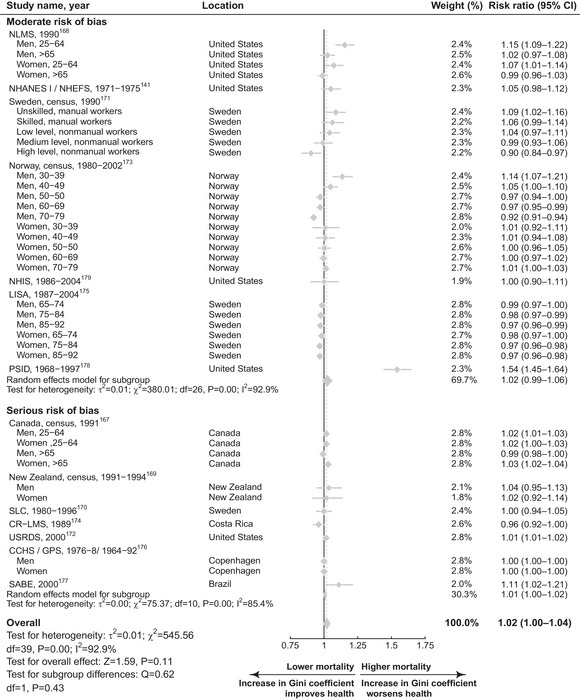
Forest Plot for a Meta‐Analysis of Studies Considering Income Inequality and All‐Cause Mortality, Stratified by RoB for a 0.05‐Unit Increase in the Gini Coefficient. The number of people were 10,727,470, and the number of studies were 14. Canada, census, Canadian Census Mortality Follow‐up Study; CCHS/GPS, Copenhagen City Heart Study; CI, confidence interval; CR‐LMS, Costa Rican Longitudinal Mortality Study; LISA, Linnaeus Database; NHANES I/NHEFS, National Health and Nutrition Examination Survey; NHIS, National Health Interview Survey; NLMS, US National Longitudinal Mortality Study; PSID, Panel Study of Income Dynamics; RoB, risk of bias; SABE, Health, Well‐Being, and Aging; SLC, Statistic Sweden's Survey of Living Conditions; USRDS: US Renal Data System.

As shown in Figure 1 and Figure 2, respectively, a 0.05‐unit increase in Gini coefficient was associated with poorer SRH (OR 1.06, 95% confidence interval [CI] 1.03‐1.08; *I* ^2^ = 96.5%; *n* = 38) but less so for mortality (RR 1.02, 95% CI 1.00‐1.04; *I* ^2^ = 92.9%, *n* = 14). When limited to studies with lower (moderate) RoB, the coefficient reduced very slightly for SRH (OR 1.05, 95% CI 1.03‐1.06; *n* = 7) and remained for mortality (RR 1.02, 95% CI 0.99‐1.06; *n* = 7), albeit with greater uncertainty.

### Meta‐Regression to Explore Reasons for Heterogeneity

The random‐effects meta‐regressions, reported in Table 2, indicated that none of the prespecified covariates (geographical scale, world region, age, time lag, length of follow‐up, adjusting for contextual variables, or RoB) appear to explain the high heterogeneity found across studies considering SRH (*I* ^2^ = 96.5%; *n* = 38) and mortality (*I* ^2^ = 92.9%, *n* = 14). Our subgroup analyses similarly did not explain sources of heterogeneity (see Appendices 19 and 20). Moreover, our sensitivity analyses in Appendix 21 indicate that decisions around which data should be in our primary analyses did not impact the overall estimates for either outcome.

**Table 2:** Meta‐Regressions Investigating Potential Sources of Heterogeneity Between Income Inequality and (a) SRH and (b) Mortality

SRH (*n* = 38)				Mortality (*n* = 14)			
Covariates	OR (95% CI)^a^	*p* Value^b^	τ^2^ ^c^	Covariates	RR (95% CI)^a^	*p* Value^b^	τ^2^ ^c^
Geographical scale^d^	Ref. group: local level, within‐country, *n* = 16			Ref. group: local level, within‐country, *n* = 10			
Regional level, within‐country (*n* = 14)^e^	1.10 (0.97‐1.25)	0.012	0.0038	Regional level, within‐country (*n* = 4)	1.10 (0.96‐1.26)	0.0021	0.0043
National level, among‐country (*n* = 8)^f^	1.03 (0.90‐1.17)	0.69	–	–	–	–	–
Mean/median income inequality^g^	Ref. group: <0.30 Gini, *n* = 5			Ref. group: <0.30 Gini coefficient, *n* = 7			
0.30‐0.40 (*n* = 16)	1.07 (0.93‐1.22)	0.32	0.0045	0.30‐0.40 (*n* = 6)	1.03 (0.88‐1.20)	0.58	0.0058
>0.40 (*n* = 17)	1.05 (0.92‐1.20)	0.60		>0.40 (*n* = 1)	1.01 (0.82‐1.25)	0.99	–
World region^h^	Ref. group: multiple world regions, *n* = 2			Ref. group: Asia and Pacific, *n* = 1			
Asia and Pacific (*n* = 7)	1.10 (0.96‐1.26)	0.027	0.0041	Europe and Central Asia (*n* = 5)	1.00 (0.87‐1.15)	0.64	0.0051
Europe and Central Asia (*n* = 11)	1.05 (0.92‐1.20)	0.12	–	Latin America and the Caribbean (*n* = 2)	1.02 (0.86‐1.22)	0.95	–
Latin America and the Caribbean (*n* = 4)	1.10 (0.95‐1.27)	0.042	–				–
North America (*n* = 14)	1.04 (0.91‐1.18)	0.18		North America (*n* = 6)	1.06 (0.92‐1.23)	0.61	–
Study setting	Ref. group: not United States, *n* = 28			Ref. group: not United States, *n* = 8			
United States (*n* = 10)	1.05 (0.92‐1.20)	0.73	0.0045	United States (*n* = 6)	1.08 (0.94‐1.24)	0.0042	0.0044
Average population age^i^	Ref. group: ≤60 years old, *n* = 33			Ref. group: ≤60 years old, *n* = 12			
>60 years old (*n* = 6)	1.04 (0.90‐1.19)	0.47	0.0044	>60 years old (*n* = 5)	0.99 (0.86‐1.14)	0.046	0.005
Covariate adjustments	Ref. group: individual‐level variables only, *n* = 16			Ref. group: individual‐level variables only, *n* = 3			
With area‐level variables (*n* = 22)	1.05 (0.92‐1.20)	0.42	0.0045	With area‐level variables (*n* = 11)	1.01 (0.89‐1.15)	0.00049	0.0042
Risk of bias	Ref. group: moderate, *n* = 7			Ref. group: moderate, *n* = 7			
Serious (*n* = 24)	1.07 (0.93‐1.22)	0.62	0.0044	Serious (*n* = 7)	1.01 (0.87‐1.18)	0.74	0.0057
Critical (*n* = 7)	1.04 (0.90‐1.19)	0.79	–	–	–	–	–
Time lag	Ref. group: <6 years, *n* = 37			Ref. group: <6 years, *n* = 13			
>6 years (*n* = 1)	1.13 (0.94‐1.36)	0.30	0.0044	>6 years (*n* = 1)	1.00 (0.80‐1.24)	0.78	0.0057
				**Follow‐Up Time: Ref. Group: <5 Years**			
				5‐10 years (*n* = 5)	1.02 (0.88‐1.19)	0.43	0.0057
				>10 years (*n* = 5)	1.03 (0.88‐1.20)	0.33	–

CI, confidence interval; mortality, all‐cause mortality; OR, odds ratio; Ref., reference; RR, risk ratio; SRH, self‐rated health.

^a^OR (95% CI) for each covariate reflects the change in poor SRH per 0.05‐unit increase in Gini coefficient. RR (95% CI) for each covariate reflects the change in mortality per 0.05‐unit increase in Gini coefficient. The values were calculated using random‐effects models with restricted maximum likelihood estimate.

^b^For testing differences, the null hypothesis coefficient has no effect.

^c^Between‐study variance explained by the covariates (i.e., residual heterogeneity beyond *I* ^2^).

^d^Geographical level describing nature of the sample (within‐country or among countries) and level at which income inequality was measured (local, regional, or national level) and nature of the sample (within‐country or among countries).

^e^Includes one study in which income inequality is measured at the national level over time, but the sample is limited to one country.

^f^Includes one study in which income inequality is measured at a regional level, but the sample spans multiple countries.

^g^Based on categories from Lin and colleagues.^51^

^h^Based on World Bank classifications.^197^

^i^Includes studies that report multiple levels of average age.^153^, ^167^, ^168^, ^173^

### Certainty of the Evidence Using the GRADE Approach

We used the GRADE approach to rate our certainty in the evidence that income inequality has a harmful, causal effect on SRH or mortality (evaluated separately; Table 3). We started with a baseline of high certainty in accordance with the ROBINS‐I approach.^47^ Across the studies considering SRH, certainty was “downgraded” by a total of four levels caused by RoB, inconsistency and publication bias, which resulted in very low certainty of a causal relationship, which is the lowest possible level of certainty described by GRADE. Most studies considering SRH were deemed to have serious or critical RoB, and as per GRADE guidance, we rated down confidence in the effect estimates caused by RoB by two levels.^200^ We downgraded certainty because of inconsistency by one level because heterogeneity was high and, based on the results from the meta‐regression, remained unexplained for studies considering SRH.^201^ Finally, we rated down certainty by one level because of publication bias after considering the funnel plot and Egger's regression test, which are both used to understand whether the study estimates are related to the sample size (as indicated by some measure of precision [e.g., standard errors]).^202^, ^203^ The Egger's test for funnel plot asymmetry was statistically significant (*p* = 0.00041) and should be considered along visual examination of the distribution of study effect estimates and standard errors in the funnel plot.^204^ Both suggested that studies with small sample sizes that find no statistically significant association are “missing” (see Appendices 22 and 23 for funnel plots). We rated down our confidence in the effect estimates for mortality because of RoB and inconsistency, resulting in low certainty that the relationship between income inequality and mortality is causal. The evidence for mortality had important methodological limitations with around half of the studies considering deemed to have serious RoB, leading to a downgrade by one level.^200^ Although studies conducted in the United States that measured the impact of income inequality on mortality reported a larger effect size than studies conducted elsewhere, more studies are needed to be conclusive on this point. We therefore also downgraded confidence in the effect estimates by one level because of inconsistency.^201^ For both SRH and mortality, we did not find any reasons to downgrade certainty because of imprecision (e.g., CIs of effect estimates include the null effect)^205^ or indirectness (e.g., evidence was incomparable with the population of interest).^206^

**Table 3:** Certainty of Evidence Using GRADE Approach

		Anticipated Absolute Effects			
Outcomes	No. of Participants (No. of Studies)	No Change in Income Inequality	0.05‐Unit Increase in Gini Coefficient	Relative Effect (95% CI)^a^	Overall Certainty of Evidence^b^
SRH	2,916,576 (*n* = 38)	21,590 per 100,000^c^	22,885 per 100,000	OR 1.06 (1.03‐1.08)	Very low^d^
Mortality (per year)	10,727,470 (*n* = 14)	925 per 100,000^e^	944 per 100,000	RR 1.02 (1.00‐1.04)	Low^f^

CI, confidence interval; mortality, all‐cause mortality; No., number; OR, odds ratio; RoB, risk of bias; RR, risk ratio; SRH, self‐rated health.

^a^OR (95% CI) for each covariate reflects the change in poor SRH per 0.05‐unit increase in Gini coefficient. RR (95% CI) for each covariate reflects the change in mortality per 0.05‐unit increase in Gini coefficient. The values were calculated using random‐effects models with restricted maximum likelihood estimate.

^b^High certainty: we are very confident that the true effect lies close to that of the estimate of the effect. Moderate certainty: we are moderately confident in the effect estimate; the true effect is likely to be close to the estimate of the effect, but there is a possibility that it is substantially different. Low certainty: our confidence in the effect estimate is limited; the true effect may be substantially different from the estimate of the effect. Very low certainty: we have very little confidence in the effect estimate; the true effect is likely to be substantially different from the estimate of effect.^86^

^c^Using data from Mutz and Lewis.^198^

^d^Certainty was rated down by two levels because of the RoB (most studies had serious or critical RoB), one level because of inconsistency (high, unexplained statistical heterogeneity), and one level because of publication bias (Egger's test *p*‐value = 0.00041). There was no change for indirectness or imprecision.

^e^Using 2019 data for England from the Office for National Statistics.^199^

^f^Certainty was rated down by one level because of RoB (half of the studies had serious RoB) and one level because of inconsistency (high, inconclusive explanations of heterogeneity). There was no change for indirectness, imprecision, or publication bias.

### Assessment of Causality Using BH Viewpoints

Our assessment using BH viewpoints found no evidence that the effects of income inequality on either SRH or mortality, reported in the meta‐analysis, were causal (Table 4). Many of the judgments regarding viewpoints were similar for SRH and mortality. We did not identify any studies that explored whether residual confounding remained after adjusting for hypothesized confounders and, therefore, found no evidence to judge specificity. We considered the RoB alongside the magnitude of the effects and the shape of the relationship to judge strength of association and the dose–response relationship, respectively, and found inconclusive evidence for both viewpoints. Plausibility and temporality were supported because the explanation for how income inequality might harm health was credible and there was no evidence of reverse causation, respectively.

**Table 4:** Causal Assessment for SRH and Mortality Using BH Viewpoints

	SRH Assessment^a^	Mortality Assessment^a^
**Doubly Decisive Viewpoint(s) (High Uniqueness, High Definitiveness)** ^b^		
Experiment	No evidence was found.	Inconclusive: we identified one natural experimental study^191^ that found that income inequality reduces the likelihood of death, although more studies are needed to draw conclusive judgments.
**Smoking Gun Viewpoint(s) (High Uniqueness, Low Definitiveness)** ^b^		
Strength of association	Inconclusive: although there are estimates showing both adverse and protective effects of income inequality, we do not believe the RoB has been properly addressed because estimates from studies with moderate RoB were smaller than from more biased studies.	Inconclusive: we observed a similar and somewhat mild adverse effect of income inequality on mortality regardless of RoB. As there does not appear to be evidence of a protective effect (as illustrated in forest plot in Figure 2), evidence for a strong (adverse) association between income inequality and mortality remains inconclusive.
Consistency	Unmet: none of the meta‐regressions, subgroup analyses, or sensitivity analyses convincingly elucidated reasons for the high between‐study heterogeneity.	Inconclusive: very high between‐study variation (*I* ^2^ = 92.9%) may be explained by contextual differences (such as United States vs. non–United‐States studies), but more studies are needed to draw conclusive judgments.
Specificity	No evidence was found. We did not identify any studies that used falsification outcomes/exposures to evaluate confounding.	
Dose–response gradient	Inconclusive: less than one‐third of studies examined a dose–response relationship between income inequality categories and SRH.^139^, ^146^, ^149^, ^151^, ^152^, ^153^, ^156^, ^157^, ^160^, ^162^, ^164^, ^165^ Moreover, only half reported a nonlinear relationship such that direction of the income inequality and SRH relationship varied across income inequality levels.	Inconclusive: fewer than one‐quarter of mortality studies could be used to evaluate a dose–response relationship, and of those four studies that did, the evidence was mixed. Most did not find a dose–response relationship, and one study reported different directions of effect for men and women.
**Hoop Viewpoints (Low Uniqueness, High Definitiveness)** ^b^		
Plausibility	Met: there are several credible (though debated) explanations of how income inequality may adversely affect health. Although most studies narratively described explanatory mechanistic evidence, and some included graphical representations of the putative relationship, to some extent, plausibility was established a priori.	
Temporality	Met: most studies (22/38, 57.9%) used different data sources for income inequality and SRH, reducing, though not eliminating, the likelihood of reverse causality. There was no clear explanation as to why some studies found poor SRH increased^140^, ^150^, ^159^, ^181^ and some that it decreased^143^, ^183^ when measured more than 6 years after income inequality.	Met: all studies considering mortality were longitudinal, which can be used to evaluate for reverse causation by adjusting for prior health.

BH, Bradford Hill; *I* ^2^, measure of between‐study variance (heterogeneity); mortality, all‐cause mortality; RoB, risk of bias; SRH, self‐rated health.

^a^See Table 1 for evidence in support of each BH viewpoint, meaning that viewpoint has been met, and evidence against each viewpoint, meaning it is unmet. Absence of supportive evidence or evidence against a viewpoint means there is “no evidence,” whereas some evidence against and some evidence in support of it means it is inconclusive.

^b^Evidence is highly unique if it does not overlap with other theories (used to confirm hypothesis). Evidence is highly definitive if *not* finding it is a strong indication that the hypothesis is untrue (disconfirms hypothesis).^101^

There were two slight differences in our judgments regarding viewpoints for SRH and mortality. First is the experiment viewpoint. We did not identify any quasi‐experimental or experimental study considering SRH and therefore could not judge this viewpoint. We identified just one^191^ natural experimental study, which found that a 0.05‐unit increase in the Gini coefficient was associated with a decreased risk of mortality, though the reported estimate was imprecise (RR 0.96, 95% CI 0.87‐1.15). We view the findings from this study we identified as potentially important because of its study design as, albeit inconclusive, evidence that income inequality does not have a causal effect on mortality. Second is consistency. Although we deemed the evidence to be unsupportive of the consistency viewpoint for studies measuring SRH because high heterogeneity remained unexplained, we found that some contextual factors may explain the differences across studies considering mortality but that this evidence was inconclusive.

## Discussion

Our findings suggest that higher levels of income inequality are associated with small increases in poor SRH and mortality after accounting for individual‐level SEP. Most studies considered income inequality measured at the local or regional, rather than the national, level. Based on our assessment using BH viewpoints, we did not find evidence that these effects were causal. A 0.05‐unit increase in the Gini coefficient was associated with a 2% (1.02 RR 95% CI 1.00‐1.04; Figure 2) increased risk of mortality using estimates from 14 studies with over ten million individuals. Pooled estimates from 38 studies with over two million individuals found that the same change in Gini coefficient increased the odds of poor SRH by 6% (OR 1.06, 95% CI 1.03‐1.08; Figure 1). Associations were similar when analyses were repeated within lower RoB studies. The conclusiveness of our findings is constrained by some key evidence gaps, including there being only one natural experimental study for mortality, no experimental or quasi‐experimental evidence for SRH, and no studies assessing the impact of income inequality on mortality at the national level among countries.

This review is the first to quantitatively summarize the relationship between income inequality and SRH and mortality in multilevel studies for many years.^20^ It is also the first to assess causality, meeting the current standards for high‐quality systematic reviews, which require methodological rigor and transparent reporting.^207^, ^208^ We used gold standard methods to conduct the review, including using the ROBINS‐I tool to critically appraise the included studies and the GRADE approach to evaluate certainty. We were transparent in our causal assessment, describing our methods in a prepublished protocol and carefully documenting minor amendments when necessary.

Our systematic review has several important limitations. First, we did not include non‐English language studies, though only six potentially relevant studies were excluded for that reason (listed in Appendix 13). Additionally, most of the studies were set in North America or Europe, which are predominantly made up of high‐income countries. Thus, our findings may not be applicable to other world regions, particularly those with many low‐ and middle‐income countries. Moreover, we could not standardize outcomes using measures of income inequality other than the Gini coefficient, meaning that our initial primary meta‐analyses assumed equivalent changes for Gini coefficient and non‐Gini coefficient measures.^50^ Studies have found that the effects of income inequality on health are similar when income inequality is measured using the Gini coefficient, Theil index, or the median share.^142^, ^143^, ^146^, ^150^, ^162^ The measure of income inequality did not explain differences across studies included in a previous meta‐analysis,^20^ whereas our sensitivity analyses similarly found that our results were not altered by including non‐Gini coefficient measures in our primary analyses. The comparability of estimates is a concern even when income inequality is measured using the same indicator. For instance, multiple versions of the Gini coefficient will often arise^209^, ^210^ because of variations in how the income and income‐sharing unit are defined,^38^ which often depend on the availability of data. Quantifying the extent to which these concerns impact our findings is challenging, as it would require reanalysis of error‐free data sets, which are unlikely to be identified.^211^ Finally, our approach to causal assessment using modified BH viewpoints is novel, and so, other researchers may have applied them differently.

There are several limitations related to the Gini coefficient that are worth noting, as it was the most common measure of income inequality. The Gini coefficient ranges from 0 (reflecting a society in which income is divided equally) to 1 (in which all income is allotted to just one individual),^212^, ^213^ with changes in inequalities occurring at different ends of the income distribution scale, potentially having different effects on health.^212^ The Gini coefficient, however, cannot distinguish between, for instance, income transferred from the richest to the poorest from changes within the middle of the income distribution scale, which both have the same value.^214^ Additionally, the small association between income inequality and health might be explained by, for instance, income inequality having an adverse effect on the health of people in the bottom half of the income distribution and a beneficial effect on those with the top share of income.^215^ More information is needed to explore this possible explanation, as the Gini coefficient cannot on its own help elucidate whether the effects of income inequality across an entire population different across subgroups within the population. We compared the effects of income inequality on SRH and mortality between studies using the Gini coefficient and a small number of studies using non‐Gini coefficient measures, some of which can account for changes in the concentration of income (see Appendices 19 and 20). Although we found no differences among the subgroups, these findings should be interpreted cautiously given the imbalance in the number of studies using these alternative measures.^216^

There have been several previous reviews evaluating the association between income inequality and SRH or mortality drawing on multilevel studies,^17^, ^20^, ^31^, ^217^, ^218^ although they were all conducted over a decade ago and have important methodological limitations and just one study quantitatively summarized the findings using in meta‐analyses.^219^, ^220^ We compare our findings with reviews that predominantly relied on studies using multilevel data that adjusted for individual‐level SEP. Our pooled estimates differ slightly from previous meta‐analyses estimates, published by Kondo and colleagues in 2008.^20^ This is perhaps unsurprising given that we included almost double the number of studies. We found a lower association between a 0.05‐unit increase in income inequality and mortality (RR 1.02, 95% CI 1.00‐1.04 [*I* ^2^ = 92.9%], compared with RR 1.08, 95% CI 1.06‐1.10 [*I* ^2^ = 96%]). In contrast, we found that the odds of poor SRH given a 0.05‐unit increase in Gini coefficient were slightly higher than, but remaining similar to, the estimates reported by Kondo and colleagues (OR 1.06, 95% CI 1.03‐1.08 [*I* ^2^ = 96.5%], compared with OR 1.04, 95% CI 1.02‐1.06 [*I* ^2^ = 88.8%]). Kondo and colleagues did not critically appraise the included studies or explicitly assess causality, but they concluded that income inequality may have a “modest” effect on health.^20^

In the review by Lynch and colleagues that had characteristics of a systematic review, they found that, for most countries, based on both ecological and multilevel studies, there was little evidence of an effect of income inequality on individual health.^17^ In their narrative synthesis, they concluded that low health insurance coverage in the United States may explain why studies set in the United States were more likely to find an adverse effect of income inequality on health than studies set in other wealthy countries (most of which found no effect). We similarly found that studies in the United States were more likely to report stronger associations for mortality than those set elsewhere, but the measures for SRH were comparable.

Our approach to causal assessment based on BH viewpoints considers the magnitude of the associations between income inequality and SRH and mortality, alongside RoB and possible explanations for heterogeneity. Estimates from studies with moderate RoB were comparable with those at serious RoB, suggesting a level of robustness to important biases such as confounding, a key consideration in nonrandomized studies. Additional evidence from studies using experimental data or addressing unmeasured confounding, an important concern in population health research, is needed to be conclusive. Future research should focus on natural experimental studies, as, though difficult to design or identify, they have the potential to decisively settle the debate over whether income inequality adversely affects health above and beyond the effect of individual income. Additional examples are limited but have included exogenous changes in income inequality during postsocialist transitions in several central and eastern European countries^221^, ^222^ and Swedish resettlement policies placing refugees in municipalities with differing income inequality levels.^191^

We could find no explanation for the high degree of heterogeneity across studies despite considering numerous possibilities. However, we did find that income inequality has a higher association with mortality for studies set in the United States when compared with those outside. This heterogeneity may be better understood through additional research estimating the impact of income inequality on mortality across multiple countries using multilevel data that would allow greater focus on among‐country comparisons. Future research should focus on improving the measurement of political and economic determinants of income inequality, including social security policies^20^ and macroeconomic development.^31^ Though difficult to measure, contextual factors such as globalization,^209^ strength of democratic institutes,^223^ or conditions in the labor market^224^, ^225^ may help determine whether findings about the relationship between income inequality and health can be applied to different settings. More research is also needed in low‐ and middle‐income countries to understand if the effects of income inequality on health in these countries differ, as is suspected, from effects in high‐income countries.^226^

It is important to underscore that our findings are limited to the effect of income inequality on SRH and mortality and that future research may find that income inequality has an effect on health inequalities^227^ or on other health outcomes than the ones we chose to focus on (e.g., subjective well‐being^228^). Our findings are also limited to the effects of income inequality on average health across whole populations, whereas future reviews that are specifically focused on the effect of income inequality across subgroups within populations might find, for instance, that income inequality has a worse effect on the health of those at the lower end of the income distribution compared with those at the higher end. Some evidence suggests that income inequality affects the health outcomes that are most likely to be impacted by an erosion in social cohesion,^229^ including suicide,^230^ drug overdoses,^231^ and mental health,^8^, ^232^, ^233^ although this evidence base might also benefit from a causally informed systematic review.

## Conclusion

The relationship between income inequality and health outcomes is complex and has been debated for many years.^232^ Here, we present a rigorous and thorough assessment of the potential causal link between income inequality and SRH and mortality after accounting for individual‐level SEP. Although we conclude that reducing income inequality per se may not improve health, the evidence base remains uncertain because of a lack of experimental and quasi‐experimental evidence and a limited number of multilevel studies examining income inequality and mortality at the national level. In the meantime, policymakers can focus their attention on evidence‐based solutions such as reducing poverty^234^ and unemployment,^235^ which have been shown to improve health across a society.

## Funding/Support

This work is supported by Wellcome Trust (218105/Z/19/Z and 205412/Z/16/Z), NHS Research Scotland (SCAF/15/02), Medical Research Council (MC_UU_00022/2), Chief Scientist Office (SPHSU17) and the European Research Council (949582). The funders played no active role in the design and conduct of the study;collection, management, analysis, and interpretation of the data; preparation, review, or approval of the manuscript; or decision to submit the manuscript for publication. All authors have completed the ICMJE uniform disclosure form at http://www.icmje.org/disclosureofinterest/ and declare no financial relationships with any organizations that might have an interest in the submitted work in the previous three years, and no other relationships or activities that could appear to have influenced the submitted work.

## Conflict of Interest Disclosures

No disclosures were reported.

## Supporting information

Online Appendix
