# Supplementary material for: Causal Assessment of Income Inequality on Self‐Rated Health and All‐Cause Mortality: A Systematic Review and Meta‐Analysis
Source: Milbank Q. 2024 Jan 31;102(1):141–82. doi: 10.1111/1468-0009.12689 (PMC10938942; doi:10.1111/1468-0009.12689)

Appendix 1 PRISMA Checklist of Reported Items in Systematic Review

| **Section and Topic** | **Item #** | **Checklist item** | **Location where item is reported** |
| --- | --- | --- | --- |
| **TITLE** | | |  |
| Title | 1 | Identify the report as a systematic review. | Title |
| **ABSTRACT** | | |  |
| Abstract | 2 | See the PRISMA 2020 for Abstracts checklist. | Abstract |
| **INTRODUCTION** | | |  |
| Rationale | 3 | Describe the rationale for the review in the context of existing knowledge. | *Introduction* |
| Objectives | 4 | Provide an explicit statement of the objective(s) or question(s) the review addresses. | *Introduction* |
| **METHODS** | | |  |
| Eligibility criteria | 5 | Specify the inclusion and exclusion criteria for the review and how studies were grouped for the syntheses. | *Search Strategy and Selection Criteria* |
| Information sources | 6 | Specify all databases, registers, websites, organisations, reference lists and other sources searched or consulted to identify studies. Specify the date when each source was last searched or consulted. | Appendix 2 (page 4) |
| Search strategy | 7 | Present the full search strategies for all databases, registers and websites, including any filters and limits used. | Appendix 3 (page 4) |
| Selection process | 8 | Specify the methods used to decide whether a study met the inclusion criteria of the review, including how many reviewers screened each record and each report retrieved, whether they worked independently, and if applicable, details of automation tools used in the process. | *Search Strategy and Selection Criteria*, Appendix 4 (page 7) and Appendix 5 (page 8) |
| Data collection process | 9 | Specify the methods used to collect data from reports, including how many reviewers collected data from each report, whether they worked independently, any processes for obtaining or confirming data from study investigators, and if applicable, details of automation tools used in the process. | *Search Strategy and Selection Criteria,* Appendix 5 (page 8) |
| Data items | 10a | List and define all outcomes for which data were sought. Specify whether all results that were compatible with each outcome domain in each study were sought (e.g., for all measures, time points, analyses), and if not, the methods used to decide which results to collect. | Appendix 6 (page 10) |
|  | 10b | List and define all other variables for which data were sought (e.g., participant and intervention characteristics, funding sources). Describe any assumptions made about any missing or unclear information. | Appendix 6 (page 10) |
| Study risk of bias assessment | 11 | Specify the methods used to assess risk of bias in the included studies, including details of the tool(s) used, how many reviewers assessed each study and whether they worked independently, and if applicable, details of automation tools used in the process. | Appendix 7 (page 12) |
| Effect measures | 12 | Specify for each outcome the effect measure(s) (e.g. risk ratio, mean difference) used in the synthesis or presentation of results. | *Selection of Multiple Effect Estimates*, Appendix 5 (page 8) |
| Synthesis methods | 13a | Describe the processes used to decide which studies were eligible for each synthesis (e.g. tabulating the study intervention characteristics and comparing against the planned groups for each synthesis (item #5)). | Appendix 5 (page 8) |
|  | 13b | Describe any methods required to prepare the data for presentation or synthesis, such as handling of missing summary statistics, or data conversions. | Appendices 5 and 8 (pages 8 and 14) |
|  | 13c | Describe any methods used to tabulate or visually display results of individual studies and syntheses. | *Data Standardization*, Appendix 5 (page 8) |
|  | 13d | Describe any methods used to synthesize results and provide a rationale for the choice(s). If meta-analysis was performed, describe the model(s), method(s) to identify the presence and extent of statistical heterogeneity, and software package(s) used. | Appendix 5 (page 8) |
|  | 13e | Describe any methods used to explore possible causes of heterogeneity among study results (e.g. subgroup analysis, meta-regression). | *Exploring heterogeneity* |
|  | 13f | Describe any sensitivity analyses conducted to assess robustness of the synthesized results. | *Data Standardization* |
| Reporting bias assessment | 14 | Describe any methods used to assess risk of bias due to missing results in a synthesis (arising from reporting biases). | Appendix 7 (page 12) |
| Certainty assessment | 15 | Describe any methods used to assess certainty (or confidence) in the body of evidence for an outcome. | *Certainty of Evidence*, Appendix 11 (page 20) |
| **RESULTS** | | |  |
| Study selection | 16a | Describe the results of the search and selection process, from the number of records identified in the search to the number of studies included in the review, ideally using a flow diagram. | *Critical Appraisal*, Appendix 12 (page 21) |
|  | 16b | Cite studies that might appear to meet the inclusion criteria, but which were excluded, and explain why they were excluded. | Appendices 9, 10, and 13 (page 22) |
| Study characteristics | 17 | Cite each included study and present its characteristics. | Appendices 14-16 (pages 42, 48, and 53) |
| Risk of bias in studies | 18 | Present assessments of risk of bias for each included study. | Appendices 17 and 18 (pages 55 and 56) |
| Results of individual studies | 19 | For all outcomes, present, for each study: (a) summary statistics for each group (where appropriate) and (b) an effect estimate and its precision (e.g. confidence/credible interval), ideally using structured tables or plots. | Figure 1 and Figure 2 |
| Results of syntheses | 20a | For each synthesis, briefly summarise the characteristics and risk of bias among contributing studies. | Figure 1 and Figure 2 |
|  | 20b | Present results of all statistical syntheses conducted. If meta-analysis was done, present for each the summary estimate and its precision (e.g. confidence/credible interval) and measures of statistical heterogeneity. If comparing groups, describe the direction of the effect. | Figure 1 and Figure 2 |
|  | 20c | Present results of all investigations of possible causes of heterogeneity among study results. | Table 2, Appendices 19 and 20 (pages 57 and 59) |
|  | 20d | Present results of all sensitivity analyses conducted to assess the robustness of the synthesized results. | Appendix 21 (page 61) |
| Reporting biases | 21 | Present assessments of risk of bias due to missing results (arising from reporting biases) for each synthesis assessed. | Appendices 22 and 23 (pages 63) |
| Certainty of evidence | 22 | Present assessments of certainty (or confidence) in the body of evidence for each outcome assessed. | Table 3 |
| **DISCUSSION** | | |  |
| Discussion | 23a | Provide a general interpretation of the results in the context of other evidence. | Table 4 and *Discussion* |
|  | 23b | Discuss any limitations of the evidence included in the review. | *Discussion* |
|  | 23c | Discuss any limitations of the review processes used. | *Discussion* |
|  | 23d | Discuss implications of the results for practice, policy, and future research. | *Discussion* |
| **OTHER INFORMATION** | | |  |
| Registration and protocol | 24a | Provide registration information for the review, including register name and registration number, or state that the review was not registered. | *Search Strategy and Selection Criteria* |
|  | 24b | Indicate where the review protocol can be accessed, or state that a protocol was not prepared. | *Search Strategy and Selection Criteria* |
|  | 24c | Describe and explain any amendments to information provided at registration or in the protocol. | Appendix 5 (page 8) |
| Support | 25 | Describe sources of financial or non-financial support for the review, and the role of the funders or sponsors in the review. | *Funding/Support* |
| Competing interests | 26 | Declare any competing interests of review authors. | *Conflict of Interest Disclosures* |
| Availability of data, code and other materials | 27 | Report which of the following are publicly available and where they can be found: template data collection forms; data extracted from included studies; data used for all analyses; analytic code; any other materials used in the review. | *Data Standardization* |

Appendix 2 Summary Search Results

|  | **Search dates** | |
| --- | --- | --- |
| **Database (supplier)** | **1992 – January 2021** | **January 1^st^, 2021 – June 6^th^, 2022** |
| Embase (OVID) | 22,783 | 1,488 |
| Medline (OVID) | 17,143 | 575 |
| Web of Science (Elsevier) | 13138 | 5,325 |
| EconLit (EBSCO) | 658 | 54 |
| Total (deduplicated) | 53,722 | 7,442 |

Appendix 3 Search Strategy and Results by Database

| **Set number** | **Searches** | **Results** |
| --- | --- | --- |
| **Embase** |  |  |
| 1 | Socioeconomic Factors/ | 96637 |
| 2 | Income/ | 55070 |
| 3 | (individual adj1 income).ab,ti. | 328 |
| 4 | (income inequality or income inequity).ab,ti. | 1464 |
| 5 | (gini adj (index or ratio or coefficient)).ab,ti. | 1158 |
| 6 | (health adj disparit*).ab,ti. | 13338 |
| 7 | Mortality/ | 693669 |
| 8 | “Cause of Death”/ | 111309 |
| 9 | Life Expectancy/ | 46946 |
| 10 | life expectancy.ab,ti. | 42401 |
| 11 | Health Status/ | 121493 |
| 12 | self-reported health.ab,ti. | 5844 |
| 13 | self- perceived health.ab,ti. | 1655 |
| 14 | self assessed health.ab,ti. | 802 |
| 15 | 1 or 2 or 3 or 4 or 5 or 6 | 159544 |
| 16 | 7 or 8 or 9 or 10 or 11 or 12 or 13 or 14 | 940357 |
| 17 | 15 and 16 | 22857 |
| 18 | limit 17 to yr = “1992 -Current” | 22783 |
|  | Identified in update (January 1, 2021 – June 6, 2022) | 1488 |
| **MEDLINE** | | |
| 1 | Socioeconomic Factors/ | 160214 |
| 2 | Income/ | 30043 |
| 3 | (individual adj1 income).ab,ti. | 266 |
| 4 | (income inequality or income inequity).ab,ti. | 1199 |
| 5 | (gini adj (index or ratio or coefficient)).ab,ti. | 795 |
| 6 | (health adj disparit*).ab,ti. | 8651 |
| 7 | Mortality/ | 45614 |
| 8 | “Cause of Death”/ | 50129 |
| 9 | Life Expectancy/ | 17778 |
| 10 | life expectancy.ab,ti. | 27137 |
| 11 | Health Status/ | 83366 |
| 12 | self-reported health.ab,ti. | 4316 |
| 13 | self- perceived health.ab,ti. | 1360 |
| 14 | self assessed health.ab,ti. | 702 |
| 15 | 1 or 2 or 3 or 4 or 5 or 6 | 191308 |
| 16 | 7 or 8 or 9 or 10 or 11 or 12 or 13 or 14 | 205693 |
| 17 | 15 and 16 | 19365 |
| 18 | limit 17 to yr = “1992 -Current” | 17143 |
|  | Identified in update (January 1, 2021 – June 6, 2022) | 575 |
| **Web of Science (Indexes = SCI-EXPANDED, SSCI, CPCI-SSH, ESCI Timespa*n =* 1992--2021)** | | |
| #1 | TS = (“Socioeconomic Factors” OR “income inequality” OR “income inequity”) OR TS = (gini NEAR (index or ratio or coefficient) ) OR TS = (“health disparit*”) OR TS = (individual NEAR income) OR TS = (income NEAR inequality) | 56004 |
| #2 | TS = (Mortality OR “cause of death” OR “life expectancy”) | 1039553 |
| #3 | TS = (self NEAR health) OR TS = (health NEAR status) | 169912 |
| #4 | #2 OR #3 | 1185754 |
| #5 | #1 AND #4 | 13138 |
|  | Identified in update (January 1, 2021 – June 6, 2022) | 5325 |
| **EconLit** | | |
| S1 | TI(“mortality rate*”) OR AB(“mortality rate*”) | 1865 |
| S2 | TI(mortality) OR AB(mortality) | 8598 |
| S3 | TI(socioeconomic) OR AB(socioeconomic) | 9412 |
| S4 | TI(“income inequalit*”) OR AB(“Income inequalit*”) | 7626 |
| S5 | TI(“life expectancy”) OR AB(“life expectancy”) | 2420 |
| S6 | TI(“gini index”) OR AB(“gini index”) | 440 |
| S7 | TI(“gini coefficient”) OR AB(“gini coefficient”) | 1152 |
| S8 | TI(“gini ratio”) OR AB(“gini ratio”) | 20 |
| S9 | S3 OR S4 OR S6 OR S7 OR S8 | 17650 |
| S10 | S1 OR S2 OR S5 | 10178 |
| S11 | S9 AND S10 | 658 |
|  | Identified in update (January 1, 2021 – June 6, 2022) | 54 |

Appendix 4 Directed Acyclic Graph Illustrating Confounding by Individual Income on the Relationship Between Income Inequality and Health

Individual-level income (*i_t_*)

Income#

Income inequality (*X_t_*)

Individual health (*y_t+1_*)

Area-level variables (e.g., income inequality, *X*) are capitalised while individual-level variables are lower-cased (e.g., individual-level income *i)* and annotated with subscript *t* to account for time. This simplified directed acyclic graph illustrates our general understanding of the relationship between income inequality and the health of all individuals in a population and is used to guide critical appraisal. Specifically, we stipulate that individual-level socioeconomic position (SEP), a confounding variable, should be adjusted for (represented by the square around individual-level SEP).

We include psychosocial factors as possible mediators of the effect of income inequality on health and adjusting for variables related to psychosial factors would reduce the effect of income inequality (a so-called ‘overadjustment’).[^195^](#bib195) While there is general consensus that individual-level SEP is a confounding variables,[^15^](#bib15)^,^ [^17^](#bib17) some have implied that SEP is a proxy measure of psychosocial factors[^4^](#bib4) which would indicate that adjusting for it would reduce some of income inequality’s effect.[^195^](#bib195) Adjusting for ‘mediator-outcome confounders’ may introduce collider bias.[^196^](#bib196) While this is a possibility, we posit that unadjusted individual SEP poses a greater risk as a confounding variable affected the exposure-outcome relationship than adjusting for individual SEP and possibly introducing collider bias. This approach is consistent with many recent reviews exploring the relationship between income inequality and health. [^17^](#bib17)^,^ [^196^](#bib196)

Finally, debate remains about whether area-level variables such as public service provision are co-exposures and thus should be conditioned upon or are mediators along the pathway (not shown) remains debated.[^12^](#bib12) As a result, we will privilage studies which test for the effects of different area-level variables.

Appendix 5 Clarifications of Review Methods and Minor Protocol Deviations

| **Review stage** | **Clarifications of methods and minor deviations from protocol** |
| --- | --- |
| **Study selection** | Deviating from our protocol, we did not include studies which explored the association between measures of relative deprivation (e.g., Yitzhaki index) and health because distribution of income, part of the relative deprivation measure, is specific to reference groups rather than across an area.[^39^](#bib39)^,^[^40^](#bib40) |
| **Causal assessment** | We incorporated process tracing to provide addition structure and clarification of causal assessment using Bradford Hill (BH) viewpoints. Process tracing is a method common in case study research and has facilitated a clear means of articulating evidence for each viewpoint and considering the implications of each viewpoint on causality. A paper detailing our approach is under review at *Research Synthesis Methods* and has been uploaded at OSF Preprints: osf.io/9wjs5. |
| **Data standardization** | Estimates[^129^](#bib129)^,^[^143^](#bib143)^,^[^145^](#bib145) using probit regression were converted to logit and Cochrane guidance was used for calculating standard errors from Z values[^130^](#bib130)^,^[^145^](#bib145)^,^[^154^](#bib154) and t-values[^144^](#bib144) as well as weighted mean standard error for studies missing standard error data. Studies evaluating the continuous odds or risk of a change in income inequality (e.g., one-unit increase in Gini coefficient) were standardized to a 0.05-unit increase.  In studies reporting estimates per levels of income inequality (e.g., lowest, low, high, and highest Gini coefficient), we used generalized least squares (GLS) regression to determine the change in self-rated health and mortality per 0.05-unit increase in Gini coefficient.[^98^](#bib98) Analysis was completed in R using the *dosresmeta* package.[^73^](#bib73) Data are available in a public, open access repository.[^74^](#bib74) Hazard ratios (HRs) and ORs were standardised to RRs based, respectively, on an approach by Zhang and Yu[^61^](#bib61) and Shor and colleagues.[^62^](#bib62)  The value or dose for each income inequality category was based on the midpoint of the reported range or, when unavailable, estimated from comparable data. We contacted authors for missing information on income inequality categories[^174^](#bib174)^,^[^156^](#bib156)^,^[^167^](#bib167)^,^[^180^](#bib180) (only[^180^](#bib180) responded with additional information). For a detailed approach to addressing missing data, see Appendix 8. |
| **Statistical analysis** | We included studies in our primary meta-analysis regardless of risk of bias (RoB) to make the pooled estimate comparable with the review on the topic, which did not appraise studies. We also included studies that reported both ordinal and dichotomous measures of self-rated health (SRH).  To avoid double counting estimates from multiple reports of the same study[^76^](#bib76) or multiple observations of a unique study (e.g., multiple observations using difference measures, method of aggregation, time point of exposure or estimate), two reviewers independently selected the most appropriate estimate to include in the primary meta-analysis as recommended by Cochrane. (However, we included estimates from multiple observations in our subgroup analysis; findings in [Appendices 19](#bib19) and [20](#bib20)). All decisions were made independently by two reviewers and disagreements were brought to a third reviewer (for complete list of decisions for multiple observations see [Appendices 9](#bib9) and [10](#bib10)). When more than one was available for the same outcome, we used the following to guide which estimate was selected (in order of importance):  • Lowest RoB  • Income inequality measured using the Gini coefficient  • Exposure does not interact with individual income  • Whole sample (vs stratified sample)  • Largest sample size  • Longest follow-up  • Continuous changes in income inequality (vs categorical)  • Regional level, within-country geographical scale (vs local level, within-country or national level, among-country)  • For SRH, cross-sectional models (vs time lag / temporal dummy variable)  We contacted authors for additional analysis using Gini coefficient if other income inequality measures were used but the authors of only one study[^129^](#bib129) provided Gini coefficient analysis while others did not have information or did not respond. Several non-Gini coefficient measures of income inequality, which we were unable to convert to a Gini coefficient scale, were nevertheless included in the primary meta-analyses. We are therefore assuming in our initial analysis that a 0.05-unit change in the Gini coefficient is equivalent to the same change in the Theil index and median share, the two non-Gini coefficient measures included. The Gini coefficient, the Theil index, and median share measures are correlated,[^50^](#bib50) and many included studies found similar effects on health regardless of the income inequality measure. A previous review also found that the choice of income inequality measure did not explain differences across estimates. Nevertheless, these measures are not directly comparable, and we therefore conducted sensitivity analyses to evaluate the impact of including those non-Gini coefficient estimates into our primary analysis (Appendix 20). |

Appendix 6 Summary of Data Extraction for Items

| **Item** | **Description** | **Categories (author’s own unless otherwise specified)** |
| --- | --- | --- |
| **Study information** | | |
| Covidence ID | Unique identifier for each article |  |
| Author | Last name of first or only author |  |
| Publication year | Year article was published |  |
| Study name | Full and abbreviated name of study |  |
| Study years | Years outcome was reported |  |
| Study location | City, country, or world region where study was based | World bank regions |
| Gini measure | Mean, median, inter-quartile range, and/or standard deviation of Gini coefficient | Low (>0.30), medium (0.30 – 0.40), high (>0.40) Gini coefficient[^51^](#bib51) |
| Geographical scale | Description and number of areas where income inequality is measured | Local (within-country), regional (within-country), International (among-countries) |
| **Study methods** | | |
| Analytical method | Reported analytical approach to multilevel modelling | Fixed effects; random effects; cluster-robust standard error |
| Study design | Type of design | Repeat cross-sectional; basic cross-sectional, longitudinal |
| Adjusted variables | Reported variables which have been adjusted for | Individual-level and area-level variables |
| **Population** | | |
| Study description | Description to indicate if estimate reflects whole sample or is stratified (e.g., by gender) |  |
| Total sample | Total participants included in analysis |  |
| Age | Mean age and/or range | 60 years old or younger, older than 60 years old |
| Gender | Proportion women |  |
| **Exposure** | | |
| Data source | Description, including year, of source of income inequality measures (e.g., Census) |  |
| Type | Categorical or continuous measure of income inequality |  |
| Metric | Income inequality metric (e.g., Gini coefficient, Theil, 90/10, median share) |  |
| Time lag | Time between income inequality measure and measure of outcome | Time lag of ≤ 6 years vs > 6 years |
| Referent and values | For each level of categorical exposure |  |
| **Outcome** | | |
| Domain | Concept to describe outcome | Self-rated health or all-cause mortality |
| Measure | Instrument used to measure outcome (e.g., lower 2 of 5 items for self-rated health) |  |
| Data source | Description, including year, of source of outcome measure (e.g., death register, surveys) |  |
| Type | Type of effect measure | Log or linear risk ratio, odds ratio, hazards ratio |
| Value | Effect estimate including measure of variance or t-test |  |

Appendix 7 Guidance for Critical Appraisal Ratings Using ROBINS-I Tool

| **Domain** | **Low** | **Moderate** | **Serious** | **Critical** |
| --- | --- | --- | --- | --- |
| Bias due to confounding | Confounding is not expected. | Confounding was sufficient, appropriate, and accurately measure. | Confounding was insufficiently addressed or improperly measured. | Confounding was inappropriate (e.g., adjusted for variables on the pathway between income inequality and individual health). |
| Bias in selection of participants into the study | All participants who were eligible for target trial were included. Low risk of selection bias was deemed more likely for evidence based on census data than surveys. | Selection based on factor(s) associated to income inequality and individual health (e.g., telephone access) but authors appropriately addressed bias or factors unlikely to introduce bias. | Selection into study was based on factors associated with income inequality and individual health but it was not addressed and appears to have introduced bias (e.g., low response rate). | Factor(s) introducing selection bias are very strongly related to both income inequality and individual health (e.g., articles selected on high-income earners) and were not addressed. |
| Bias due to missing information | Studies reported no missing data or analysis used to consider differences between groups. | Missingness appears to be balanced between groups. | Reasons/proportions for missingness differ substantially OR reasons/proportions were not provided but overall missing data appears meaningful | Reasons and/or proportion of missing information like to introduce bias and was not addressed. |
| Bias due to deviations from intended interventions | Participants did not deviate from intended intervention or that deviation did not affect the outcome. | Deviations from the level of income inequality were moderate. | Deviations were deemed to have a considerable effect on health (e.g., among-country measures that do not account for differences across countries such as GDP). | Deviations were too substantial and impactful to be addressed via analysis. |
| Bias in measurement of results | Studies explicitly note that assessors were blind to result measurement. | Unlikely that assessors knew of level of income inequality of participants (e.g., income inequality measured exogenously). | Possible that assessors knew both income inequality level and health (e.g., assessors both conducted analysis and collected health data). | Methods of assessing outcome are different between comparison groups. |
| Bias in selection of the reported result | Studies detail analysis a priori in a published protocol. | Studies did not publish protocol, but no evidence of selective reporting. | Reviewers suspect selection of reported results based on findings. | Evidence that results were selected based on findings. |
| Bias in classification of interventions | Clearly defined intervention. | Reviewers can make strong assumptions of interventions based on other information reported. | Intervention is not clearly defined and cannot be determined based on additional information (e.g., inputting missing data for income inequality levels). | Misclassification of intervention is substantial (e.g., recall bias) (unlikely) |

Appendix 8 Approach for Addressing Missing Reported Data

| Type of missing data | Studies with missing data | Approach for addressing missing data |
| --- | --- | --- |
| Income inequality value | Latif 2015[^181^](#bib181) | World Bank Development Research Group[^52^](#bib52) |
|  | Subramanian et al., 2003a[^159^](#bib159) | Zheng, 2009[^166^](#bib166) |
|  | Evans, 2020[^139^](#bib139) | Vauclair et al., 2015[^161^](#bib161) |
|  | Baum, 2016[^130^](#bib130) | Organisation for Economic Co-operation and Development Statistics[^53^](#bib53) |
|  | Chiavegatto Filho et al., 2012[^136^](#bib136) | Pabayo et al., 2013[^177^](#bib177) |
|  | Auger et al., 2012[^137^](#bib137) | Breau, 2015[^53^](#bib53) (1996 Gini coefficients) |
|  | Backlund et al., 2007[^168^](#bib168) | Lynch et al., 1988[^55^](#bib55) |
|  | Zheng 2012[^179^](#bib179) | Lochner et al., 2001[^104^](#bib104) |
|  | Kimmel et al., 2013[^172^](#bib172) | Lopez, 2005[^113^](#bib113) |
|  | Osler 2002[^176^](#bib176) | Gustafsson and Johansson, 1999[^56^](#bib56) (1987/1992 median share) |
|  | Massa et al., 2018[^153^](#bib153); Massa et al., 2021[^182^](#bib182); Shibuya, 2002[^157^](#bib157); Xi, 2005[^165^](#bib165); Ng et al., 2020[^175^](#bib175) | If not otherwise reported, calculated range of Gini coefficient values based on mean, standard deviation and overall range.[^57^](#bib57) If not available, Gini coefficient mean and standard deviation were estimated using method recommended by Cochrane.[^58^](#bib58)^,^[^70^](#bib70) |
|  | Hou, 2005[^146^](#bib146) | Breau, 2015[^54^](#bib54) (2006 Gini coefficients) |
|  | Choi et al., 2015[^147^](#bib147) | Used midpoint of highest and lowest income inequality categories to estimate continuous increase in income inequality. |
| **Missing confidence intervals** | Evans et al., 2020[^139^](#bib139); Karlsdotter et al., 2012[^150^](#bib150); LeClere and Soobader 2000[^152^](#bib152) | Standard error calculated from weighted means of all standard errors using method recommended by Cochrane.[^58^](#bib58)^,^ [^70^](#bib70) |
| **Reported outcomes** | Caicedo-Velasquez and Restrepo-Mendez 2020[^180^](#bib180) | Author sent total number of individuals and number of individuals with poor SRH in each category. |
|  | Kravdal, 2008[^173^](#bib173) | Author said to use number of deaths extracted from Kondo et al., 2009[^20^](#bib20) |

Appendix 9 Recorded Decisions for Multiple Observations: Self-Rated Health

| Study name | Included reports by analysis type (all analyses[^a^](#tbfnafn1) or additional analysis only[^b^](#tbfnafn2)) | Excluded duplicate reports[^c^](#tbfnafn3) |
| --- | --- | --- |
| Australia | | |
| Household Income and Labour Dynamics in Australia (HILDA) survey | All analyses: Baum et al., 2016[^130^](#bib130) |  |
| Brazil | | |
| Health, Well-Being and Aging study | All analyses: Chiavegatto et al., 2012[^136^](#bib136) |  |
| National Health Survey (PNS) | All analyses: Massa et al., 2018[^153^](#bib153)  Average population age: Massa and Chiavegatto, 2021[^182^](#bib182) |  |
| Canada | | |
| Canadian National Population Health Survey (CNPHS) | All analyses: Hou and Myles, 2005[^146^](#bib146)  Time lag analysis: Latif and Policy, 2015[^181^](#bib181) | Study length: Hou and Chen, 2003[^112^](#bib112)  Exposure measure: McLeod et al., 2003[^117^](#bib117) |
| Ontario Health Survey (OHS), an expansion of the National Population Health Survey (NPHS) | All analyses: Xi et al., 2005[^165^](#bib165) |  |
|  |  |  |
| Chile | | |
| National Socioeconomic Characterization Survey (CASEN) | All analyses: Subramanian et al., 2003[^160^](#bib160) |  |
| China | | |
| China Health and Nutrition Survey (CHNS) | All analyses: Cai et al., 2020[^134^](#bib134)  Time lag analysis: Pei and Rodriguez, 2006[^183^](#bib183) | Less information: Fang and Rizzo, 2012[^111^](#bib111); Li and Zhu, 2006[^113^](#bib113); Ting et al., 2022[^127^](#bib127) |
| Chinese Longitudinal Healthy Longevity Survey (CLHLS) | All analyses: Feng et al., 2012[^140^](#bib140) |  |
| Colombia | | |
| Departamento Administrativo Nacional de Estadística (DANE) | All analyses: Góngora-Salazar et al., 2022[^142^](#bib142)  Geographical scale: Caicedo-Velasquez and Restrepo, 2020[^180^](#bib180) |  |
| Europe (between countries) | | |
| European Community Household Panel survey (ECHP) | All analyses: Hildebrand and Van, 2009[^145^](#bib145) |  |
| New Democracies Barometer (NDB), New Baltic Barometer (NBB), and New Russia Barometer (NRB) | All analyses: Bobak et al., 2000[^133^](#bib133) |  |
| New European Barometer (NEB) | All analyses: Bobak et al., 2007[^132^](#bib132) |  |
| European Quality of Life Surveys (EQLS) | All analyses: Evans et al., 2020[^139^](#bib139) | Less information: Zagorski et al., 2014[^128^](#bib128) |
| European Social Survey (ESS) | All analyses: Vauclair et al., 2015[^161^](#bib161) |  |
| Survey of Health, Ageing and Retirement in Europe (SHARE) | All analyses: Adeline and Delattre, 2017[^129^](#bib129) | Less information: Pasqualini et al., 2017[^121^](#bib121) |
| Hong Kong | | |
| Thematic Household Surveys (THS) | All analyses: Wong et al., 2009[^164^](#bib164) |  |
| Japan | | |
| Aichi Gerontological Evaluation Study Project (AGES) | All analyses: Ichida et al., 2009[^147^](#bib147) | Sample size: Aida et al., 2011[^107^](#bib107) |
| Comprehensive Survey of Living Conditions of People on Health and Welfare (CSLCPHW) | All analyses: 1995 - Shibuya et al., 2002[^157^](#bib157)  All analyses: 2004 - Oshio and Kobayashi, 2009[^154^](#bib154) | Oshio and Kobayashi 2010[^120^](#bib120) |
| Spain | | |
| Life Conditions Survey (LCS) | All analyses: Karlsdotter et al., 2012[^150^](#bib150) |  |
| Sweden | | |
| Stockholm County Council’s Public Health Questionnaire (PHQ) | All analyses: Rostila et al., 2012[^156^](#bib156) |  |
| United Kingdom | | |
| British Household Panel Survey (BHPS) | All analyses: Weich et al., 2002[^162^](#bib162) | Less information: Lorgelly and Lindley, 2008[^115^](#bib115) |
| British General Household Survey (GHS) | All analyses: Gravelle and Sutton, 2009[^143^](#bib143) |  |
| Scottish household survey (SHS) | All analyses: Craig, 2005[^138^](#bib138) |  |
| United States | | |
| Behavioral Risk Factor Surveillance System (BRFSS) | All analyses: 1993--1994 - Kennedy et al., 1998[^151^](#bib151)  All analyses: 2000 - Chen and Gotway, 2012[^135^](#bib135)  All analyses: 2014--2016 - Haithcoat et al., 2021[^144^](#bib144)  Covariate adjustments: Subramanian et al., 2001[^185^](#bib185) | Sample size: Lopez 2004[^114^](#bib114) |
| Los Angeles Family and Neighborhood Survey (L.A. FANS) | All analyses: Bjornstrom and Medicine, 2011[^131^](#bib131) |  |
| Current Population Survey (CPS) | All analyses: Subramanian et al., 2003[^159^](#bib159)  Time lag analysis: Subramanian and Kawachi, 2003[^184^](#bib184) | Less information: Subramaniam and Kawachi, 2006[^126^](#bib126)  Categorical model: Blakely et al., 2002[^110^](#bib110); Shi and Starfield, 2000[^122^](#bib122); Blakely et al., 2000[^108^](#bib108)  Co-interventions: Blakely et al., 2001[^109^](#bib109)  Outcome measure: Mellor and Milyo, 2002[^118^](#bib118), Mellor and Milyo, 2003[^119^](#bib119) |
| General Social Survey (GSS) | All analyses: 1972--2004 - Zheng, 2009[^166^](#bib166)  All analyses: 2006--2016 - Sommet and Elliot, 2022[^158^](#bib158) |  |
| Health and Retirement Study (HRS) | All analyses: Choi et al., 2015[^137^](#bib137) |  |
| Metropolitan Chicago Information Center Metro Survey (MCIC-MS) | All analyses: Wen et al., 2003[^163^](#bib163) |  |
| National Health and Nutrition Examination Survey (NHANES) | All analyses: Fiscella and Franks, 2000[^141^](#bib141) |  |
| National Health Interview Survey (NHIS) | All analyses: Leclere and Soobader, 2000[^152^](#bib152) | Sample size: Soobader and Leclere, 1999[^125^](#bib125) |
| National Longitudinal Survey of Youth (NLSY) | Non-Gini coefficient estimate sensitivity analysis only: Zimmerman and Bell, 2006[^186^](#bib186) |  |
| National Maternal Infant Health Survey (NMIHS) | All analyses: Kahn et al., 2000[^149^](#bib149) |  |
| **Multiple world regions** | | |
| World Values Survey (WVS) | All analyses: 1981--2000 - Jen et al., 2009[^148^](#bib148)  All analyses: 2005 -2007 - Qi, 2012[^155^](#bib155) | Sample size: Rozer et al., 2016[^121^](#bib121); Rozer and Volker, 2016[^123^](#bib123)  Study length: Mansyur et al., 2008[^116^](#bib116) |

^a^All analyses (n = 38)[^129^](#bib129)^-^[^166^](#bib166) = in primary analysis, meta-regressions, and subgroup analyses and/or sensitivity analyses.

^b^Additional analyses (n = 7)[^180^](#bib180)^,^[^186^](#bib186) = in subgroup and/or sensitivity analyses only. Key subgroup/sensitivity analyses: Covariate adjustments = subgroup and/or sensitivity analyses with multiple models adjusting for different variables. Geographical scale = subgroup and/or sensitivity analyses with income inequality measured across multiple geographical scales (e.g., regional within-country and local within-country). Time lag = subgroup and/or sensitivity analyses with different time lags between income inequality and health outcome.

^c^Duplicates (n = 22)[^107^](#bib107)^,^[^128^](#bib128) = articles with duplicate information deemed less relevant than articles in primary and/or additional analyses. Less information = less study detail provided (e.g., do not report sample size or confidence intervals). Study length = shorter follow-up time or fewer repeated cross-sections. Sample size = smaller sample size. Exposure measure = income inequality measured using non-Gini coefficient exposure. Categorical model = levels of income inequality (vs continuous change). Co-intervention: exposure is multifactorial (i.e., income inequality alongside another exposure). Outcome measure = reports ordinal outcome (vs binary) or marginal effects.

Appendix 10 Recorded Decisions for Multiple Observations: All-Cause Mortality

| Study name | Included reports by analysis type (all analyses[^a^](#tbfnafn1) or additional analysis only[^b^](#tbfnafn2)) | Excluded duplicate reports[^c^](#tbfnafn3) |
| --- | --- | --- |
| Brazil | | |
| Health, Well-Being, and Aging (SABE) Survey | All analyses: Pabayo et al., 2013[^177^](#bib177) |  |
| Canada | | |
| Canadian Census Mortality Follow-up Study (CCMFS) | All analyses: Auger et al., 2012[^167^](#bib167) |  |
| Copenhagen | | |
| Copenhagen City Heart Study (CCHS), Glostrup Population Study (GPS) | All analyses: Osler et al., 2002[^176^](#bib176) |  |
| Costa Rica | | |
| Costa Rican Longitudinal Mortality Study (CR-LMS) | All analyses: Modrek et al., 2012[^174^](#bib174) |  |
| Hong Kong | | |
| Study name not specified | Sensitivity analysis of abstract-only studies: Lau et al., 2011[^194^](#bib194) |  |
| New Zealand | | |
| New Zealand census | All analyses: Blakely et al., 2003[^169^](#bib169) |  |
| Norway | | |
| Norway census | All analyses: Kravdal, 2008[^173^](#bib173)  Geographical scale: Dahl, 2006[^188^](#bib188)  Covariate adjustments: Elstad, 2011[^190^](#bib190) | Outcome measure: Kinge, 2015[^104^](#bib104) |
| Sweden | | |
| Linnaeus Database | All analyses: Ng et al., 2020[^175^](#bib175) | Study length: Edvinsson et al., 2013[^101^](#bib101) |
| Statistic Sweden’s Survey of Living Conditions (ULF study) | All analyses: Gerdtham and Johannesson, 2004[^170^](#bib170)  Grönqvist et al., 2012[^191^](#bib191) |  |
| Sweden census | All analyses: Henriksson, et al., 2007[^171^](#bib171)  Sensitivity analysis of non-Gini coefficient studies: Henriksson et al., 2006[^192^](#bib192) |  |
| United States | | |
| Cancer Prevention Study-II (CPS-II) | Sensitivity analysis of studies which do not address clustering: Kahn et al., 1999[^193^](#bib193) |  |
| National Health and Nutrition Examination Survey (NHANES I) Epidemiologic Follow-up Study (NHEFS) | All analyses: Fiscella and Franks, 2000[^141^](#bib141)  Covariate adjustments: Fiscella and Franks, 1997[^187^](#bib187) |  |
| National Health Interview Survey (NHIS) | All analyses: Zheng, 2012[^179^](#bib179) | Sample size: Eibner and Evans, 2005[^102^](#bib102)  Study length: Lochner et al., 2001[^105^](#bib105); Waitzman et al., 1999[^106^](#bib106) |
| United States National Longitudinal Mortality Study (NLMS) | All analyses: Backlund et al., 2007[^168^](#bib168) | Co-interventions: Kim, 2016[^103^](#bib103) |
| Panel Study of Income Dynamics (PSID) | All analyses: Zhao et al., 2021[^178^](#bib178) Sensitivity analysis of studies which do not address clustering: Daly 1998[^189^](#bib189) |  |
| United States Renal Data System (USRDS) | All analyses: Kimmel et al., 2013[^172^](#bib172) |  |

^a^All analyses (n = 14)[^167^](#bib167)^-^[^179^](#bib179)^,^[^142^](#bib142) = in primary analysis, meta-regressions, and subgroup analyses and/or sensitivity analyses.

^b^Additional analyses (n = 8)[^187^](#bib187)^,^[^194^](#bib194) = in subgroup and/or sensitivity analyses only. Key subgroup/sensitivity analyses: Covariate adjustments = subgroup and/or sensitivity analyses with multiple models adjusting for different variables. Geographical scale = subgroup and/or sensitivity analyses with income inequality measured across multiple geographical scales (e.g., regional within-country and local within-country). Time lag = subgroup and/or sensitivity analyses with different time lags between income inequality and health outcome.

^c^Duplicates (n = 6)[^101^](#bib101)^,^[^106^](#bib106) = articles with duplicate information deemed less relevant than articles in primary and/or additional analyses. Study length = shorter follow-up time or fewer repeated cross-sections. Sample size = smaller sample size. Co-interventions: exposure is multifactorial (i.e., income inequality alongside another exposure). Outcome measure = reports ordinal outcome (vs binary) or marginal effects.

Appendix 11 Matrix of Bradford Hill Viewpoints by Process Tracing Types


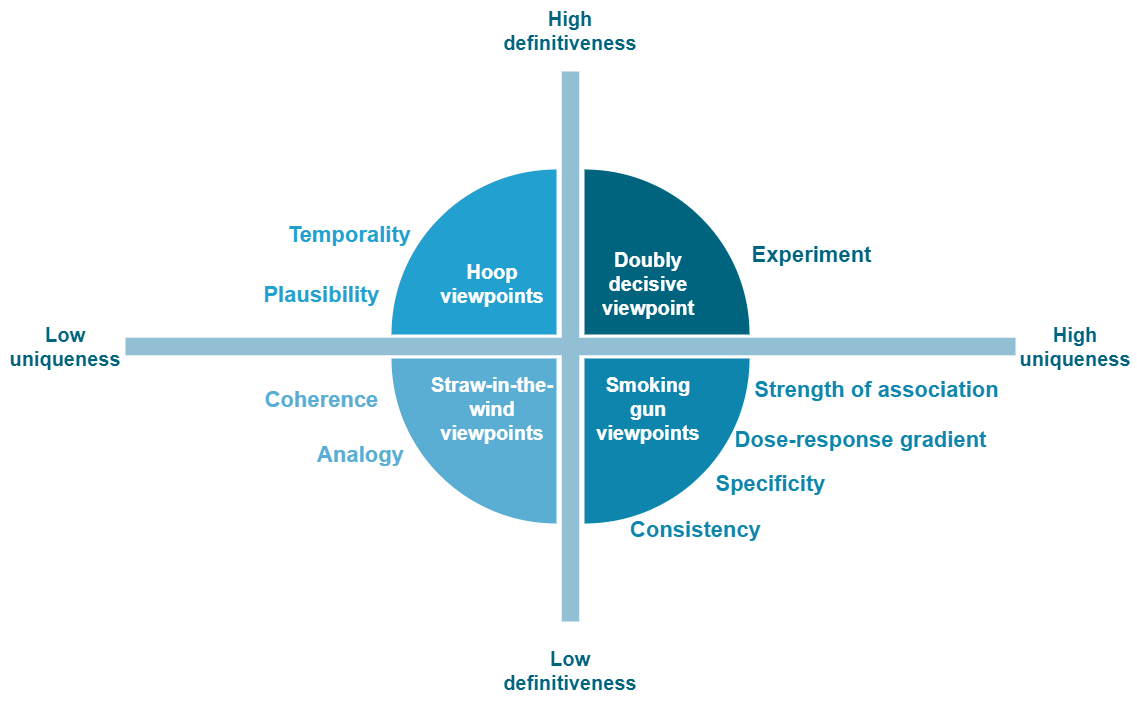


Finding evidence in support of a highly unique viewpoint can confirm the hypothesis while finding evidence against a highly definitive viewpoint can disconfirm the hypothesis.

Appendix 12 PRISMA Flowchart


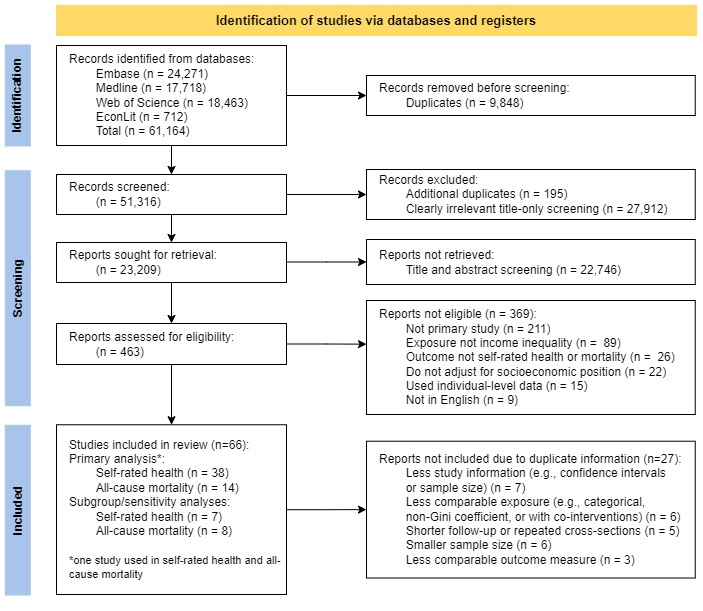


Flowchart detailing the database searches, the number of titles and abstracts screened, the full texts retrieved, and studies included in the primary and/or subgroup/sensitivity analyses for each outcome.

Appendix 13 Reasons for Excluding Retrieved Full Texts

| Excluded study | Reason^a^ |
| --- | --- |
| Aberg Yngwe M, Kondo N, Hagg S, Kawachi I. Relative deprivation and mortality–a longitudinal study in a Swedish population of 4.7 million, 1990–2006. BMC Public Health. 2012;12:664. | 2 |
| Adjaye-Gbewonyo K, Kawachi I. Use of the Yitzhaki Index as a test of relative deprivation for health outcomes: a review of recent literature. Soc Sci Med. 2012;75(1):129-37. | 4 |
| Alam MS, Islam MS, Shahzad SJH, Bilal S. Rapid rise of life expectancy in Bangladesh: does financial development matter? Int J Finance & Econ. 2020. | 4 |
| Alam MS, Shahbaz M, Paramati SR. The role of financial development and economic misery on life expectancy: evidence from post financial reforms in India. Social Indicators Res. 2016;128(2):481-497. | 4 |
| Allen J, Chakraborty S. Aspirations, health and the cost of inequality. J Economic Dynamics & Control. 2018;86:144-164. | 4 |
| Almeida G, Sarti FM, Ferreira FF, Diaz MDM, Campino ACC. Income-related inequality and inequity in children’s health care: a longitudinal analysis using data from Brazil. Revista Panamericana de Salud Pública. 2013;33(2):90-97. | 4 |
| Anonymous. Income inequality and mortality among working-age people in Canada and the US. Health Rep. 1999;11(3):77-82(Eng); 5. | 4 |
| Arno PS, House JS, Viola D, Schechter C. Social security and mortality: the role of income support policies and population health in the United States. J Public Health Policy. 2011;32(2):234-250. | 2 |
| Asafu-Adjaye J. Income inequality and health: a panel data analysis of transition economies. In: Roy KC, Chatterjee S, eds. Growth, development, and Poverty Alleviation in the Asia-Pacific. Nova Publishers; 2007:91-110. | 4 |
| Asgeirsdottir TL, Ragnarsdottir DO. Health-income inequality: the effects of the Icelandic economic collapse. Int J Equity in Health. 2014;13:50. | 2 |
| Ash M, Robinson DE. Inequality, race, and mortality in U.S. cities: a political and econometric review of Deaton and Lubotsky. Soc Sci Med. 2009;68(11):1909-13. | 4 |
| Auger N, Zang G, Daniel M. Community-level income inequality and mortality in Quebec, Canada. Public Health. 2009;123(6):438-443. | 5 |
| Avendano M, Hessel P. The income inequality hypothesis rejected? Eur J Epidemiol. 2015;30(8):595-598. | 4 |
| Babones SJ. Income inequality and population health: correlation and causality. Soc Sci Med. 2008;66(7):1614-1626. | 4 |
| Baek SH, Kim KT. Retesting the income inequality hypothesis: pooled time-series-cross-section regression with a new statistical case selection method. Asian Social Work and Policy Review. 2018;12(3):191-199. | 5 |
| Bagger JP. Taxation and life expectancy in Western Europe. Central Eur J Public Health. 2004;12(2):113-114. | 2 |
| Baker D, Illsley R. Income distribution and life expectancy. BMJ. 1992;304(6828):715. | 4 |
| Bakkeli NZ. Income inequality and health in China: a panel data analysis. Soc Sci Med. 2016;157:39-47. | 3 |
| Balia S, Jones AM. Mortality, lifestyle and socio-economic status. J Health Econ. 2008;27(1):1-26. | 2 |
| Banerjee N, De A, Basu R, Poali M. Global inequality in life expectancies: what role does income inequality play? Econ Bulletin. 2021;41(2):553-563. | 4 |
| Basky G. Death and tax brackets: link between income inequality and mortality holds true in US, but not in Canada. CMAJ. 2000;162(13):1866. | 4 |
| Bassino JP. Inequality in Japan (1892-1941): physical stature, income, and health. Econ & Human Biology. 2006;4(1):62-88. | 2 |
| Beckfield J. Does income inequality harm health? New cross-national evidence. J Health Soc Behav. 2004;45(3):231-248. | 5 |
| Bezruchka S. Income inequality and population health. Hierarchy and health are related. BMJ. 2002;324(7343):978. | 4 |
| Biggs B, King L, Basu S, Stuckler D. Is wealthier always healthier? The impact of national income level, inequality, and poverty on public health in Latin America. Soc Sci Med. 2010;1(2):266-273. | 2 |
| Blakely T, Wilson N. Shifting dollars, saving lives: what might happen to mortality rates, and socio-economic inequalities in mortality rates, if income was redistributed? Soc Sci Med. 2006;62(8):2024-2034. | 4 |
| Blakely T, Woodward A. Income inequality and mortality in Canada and the United States – third explanation is plausible. BMJ. 2000;321(7275):1532-1533. | 4 |
| Blakely TA, Kawachi I. Education does not explain association between income inequality and health. BMJ. 2002;324(7349):1336. | 4 |
| Blazquez-Fernandez C, Cantarero-Prieto D, Pascual-Saez M. Does rising income inequality reduce life expectancy? New evidence for 26 European countries (1995-2014). Global Economic Review. 2018;47(4):464-479. | 4 |
| Bocoum I, Tohon AB, Rukundo R, Macombe C, Reveret JP. Effect of income inequality on health in Quebec: new insights from panel data. Sustainability. 2019;11(20). | 4 |
| Bonneuil N. Health Component of inequalities associated with income mobility over the life cycle. Social Indicators Res. 2019;141(1):391-411. | 3 |
| Brekke KA, Kverndokk S. Inadequate bivariate measures of health inequality: the impact of income distribution. Scandinavian J Econ. 2012;114(2):323-333. | 4 |
| Brezzi M, De Mello L. Inequalities in Latin America: trends and implications for policy. Hacienda Publica Espanola-Review of Public Econ. 2016(219). | 4 |
| Brodish PH, Hakes JK. Quantifying the individual-level association between income and mortality risk in the United States using the National Longitudinal Mortality Study. Soc Sci Med. 2016;170:180-187. | 2 |
| Brodish PH, Massing M, Tyroler HA. Income inequality and all-cause mortality in the 100 counties of North Carolina. Southern Medical Journal. 2000;93(4):386-391. | 4 |
| Bronnum-Hansen H, Foverskov E, Andersen I. Income inequality in life expectancy and disability-free life expectancy in Denmark. J Empidemiol and Community Health. 2020;10. | 4 |
| Bütikofer A, Karadakic R, Salvanes KG. Income Inequality and mortality: a Norwegian perspective*. Fiscal Studies. 2021;42(1):193-221. | 3 |
| Cabieses B, Espinoza MA. Exploring income inequality in self-reported health status in chile after the health care reform of 2005. Value in Health. 2013;16(7):A679-A680. | 4 |
| Caicedo B, Fernandez DB. Self-rated health in adults: influence of poverty and income inequality in the area of residence. Gaceta Sanitaria. 2015;29(2):97-104. | 6 |
| Caner A, Yigit YC. Relative deprivation and its association with health indicators: Lower inequality may not improve health. Soc Sci Med – Population Health. 2019;7. | 4 |
| Cantarero D, Pascual M, Sarabia JM. Effects of income inequality on population health: new evidence from the European community household panel. App Econ. 2005;37(1):87-91. | 4 |
| Chan CH, Wong HK, Yip PSF. Associations of relative income deprivation with perceived happiness and self-rated health among the Hong Kong Chinese population. Int J Public Health. 2017;62(6):697-707. | 2 |
| Chan MF, Taylor BJ. Impact of demographic change, socioeconomics, and health care resources on life expectancy in Cambodia, Laos, and Myanmar. Public Health Nurs. 2013;30(3):183-192. | 2 |
| Chandola T, Mikkilineni S, Chandran A, Bandyopadhyay SK, Zhang N, Bassanesi SL. Is socioeconomic segregation of the poor associated with higher premature mortality under the age of 60? A cross-sectional analysis of survey data in major Indian cities. BMJ. 2018;8(2). | 2 |
| Chaudhry R, Dranitsaris G, Mubashir T, Bartoszko J, Riazi S. A country level analysis measuring the impact of government actions, country preparedness and socioeconomic factors on COVID-19 mortality and related health outcomes. EclinicalMedicine. 2020;25 (no pagination). | 2 |
| Chia-Chang C. The mediating roles of GDP on the relationship between income inequality and life expectancy. Asian Academy of Management Journal. 2021;26(2):101-116. | 5 |
| Chiang T. Economic transition and changing relation between income inequality and mortality in Taiwan: regression analysis. BMJ. 1999;319(7218):1162-1165. | 4 |
| Chiavegatto ADP, Kawachi I, Gotlieb SLD. Propensity score matching approach to test the association of income inequality and mortality in Sao Paulo, Brazil. J Epidemiol and Community Health. 2012;66(1):14-17. | 4 |
| Clark R, Snawder K. A Cross-national analysis of lifespan inequality, 1950--2015: examining the distribution of mortality within countries. Social Indicators Res. 2020;148(3):705-732. | 4 |
| Clarkwest A. Neo-materialist theory and the temporal relationship between income inequality and longevity change. Soc Sci Med. 2008;66(9):1871-1881. | 4 |
| Clough-Gorr KM, Egger M, Spoerri A. A Swiss paradox? Higher income inequality of municipalities is associated with lower mortality in Switzerland. Eur J Epidemiology. 2015;30(8):627-636. | 5 |
| Coburn D. Income inequality, social cohesion and the health status of populations: the role of neo-liberalism. Soc Sci Med. 2000;51(1):135-146. | 4 |
| Coburn D. Beyond the income inequality hypothesis: class, neo-liberalism, and health inequalities. Soc Sci Med. 2004;58(1):41-56. | 2 |
| Cooper D, McCausland WD, Theodossiou I. Income inequality and wellbeing: the plight of the poor and the curse of permanent inequality. J Economic Issues. 2013;47(4):939-957. | 3 |
| Cooper RS, Kennelly JF, Durazo-Arvizu R, Oh HJ, Kaplan G, Lynch J. Relationship between premature mortality and socioeconomic factors in black and white populations of US metropolitan areas. Public Health. 2001;116(5):464-473. | 4 |
| Costa G, Marinacci C, Caiazzo A, Spadea T. Individual and contextual determinants of inequalities in health: the Italian case. Int J Health Serv. 2003;33(4):635-667. | 2 |
| Costa-Font J, Hernandez-Quevedo C. Concentration Indices of income-related self-reported health: a meta-regression analysis. App Economic Perspectives and Policy. 2015;37(4):619-633. | 4 |
| Cramm JM, Moller V, Nieboer AP. Individual- and neighbourhood-level indicators of subjective well-being in a small and poor eastern cape township: the effect of health, social capital, marital status, and income. Social Indicators Res. 2012;105(3):581-593. | 3 |
| Cukur A, Bekmez S. Examining the relationship between income inequality and self assessed health in Turkey with nationally representative health survey data. Iktisat Isletme Ve Finans. 2012;27(314):73-104. | 6 |
| Curran M, Mahutga MC. Income inequality and population health: a global gradient? J Health and Social Behavior. 2018;59(4):536-553. | 4 |
| Currie J, Schwandt H, Thuilliez J. Pauvrete, egalite, mortalite: mortality (in)equality in France and the United States. J Population Econ. 2020;33(1):197-231. | 4 |
| Dachs JNW, Ferrer M, Florez CE, Barros AJD, Narvaez R, Valdivia M. Inequalities in health in Latin America and the Caribbean: Descriptive and exploratory results for self-reported health problems and health care in twelve countries. Revista Panamericana de Salud Pública. 2002;11(5-6):335-355. | 2 |
| Daly MC, Wilson DJ. Inequality and mortality: new evidence from U.S. county panel data. Federal Reserve Bank of San Francisco, Working Paper Series: 2013-13; 2013. | 1 |
| Davey Smith G. Income inequality and mortality: Why are they related? BMJ. 1996;312(7037):987-8. | 1 |
| Davila MG, Polanco VP, Santaella J. Income Inequality and Life Expectancy in the United States. Am J Public Health. 2016;106(8):1350-. | 1 |
| De Maio F, Linetzy B, Ferrante D. Income inequality and self-rated health: ecological results from the 2005 / 2009 Argentine National Risk Factor Surveys. Am J Epidemiol. 2011;173:S145-S. | 1 |
| De Maio FG. Ecological analysis of the health effects of income inequality in Argentina. Public Health. 2008;122(5):487-96. | 1 |
| De Maio FG, Linetzky B, Ferrante D, Fleischer NL. Extending the income inequality hypothesis: ecological results from the 2005 and 2009 Argentine National Risk Factor Surveys. Glob Public Health. 2012;7(6):635-47. | 1 |
| De Vogli R, Ferrie JE, Chandola T, Kivimaki M, Marmot MG. Unfairness and health: Evidence from the Whitehall II Study. J Epidemiol Community Health. 2007;61(6):513-8. | 2 |
| De Vogli R, Mistry R, Gnesolto R, Cornia GA. Has the relationship between income inequality and life expectancy disappeared? Evidence from Italy and top industrialised countries. J Epidemiol Community Health. 2005;59(2):158-62. | 4 |
| Deaton A. Inequalities in income and inequalities in health. Princeton University, Woodrow Wilson School of Public and International Affairs, Center for Health and Wellbeing., Working Papers: 280; 1999. | 1 |
| Deaton A, Lubotsky D. Mortality, Inequality and Race in American Cities and States. National Bureau of Economic Research, Inc, NBER Working Papers: 8370; 2001. | 1 |
| Deaton A, Lubotsky D. Income inequality and mortality in U.S. cities: Weighing the evidence - A response to Ash. Princeton University, Woodrow Wilson School of Public and International Affairs, Center for Health and Wellbeing., Working Papers: 1166; 2009. | 1 |
| Deaton A, Paxson C. Health, income, and inequality over the life cycle. 1998. | 1 |
| Deaton A, Paxson C. Mortality, Income, and Income Inequality over Time in Britain and the United States. NBER Conference Report series.Chicago and London:University of Chicago Press; 2004. | 1 |
| Decoster A, Minten T, Spinnewijn J. The Income Gradient in Mortality during the Covid-19 Crisis: Evidence from Belgium. The J Economic Inequality. 2021;19(3):551-70. | 2 |
| Deeming C, Jones K. Investigating the Macro Determinants of Self-Rated Health and Well-Being Using the European Social Survey: Methodological Innovations across Countries and Time. Int J Sociology. 2015;45(4):256-85. | 2 |
| Detollenaere J, Desmarest AS, Boeckxstaens P, Willems S. The link between income inequality and health in Europe, adding strength dimensions of primary care to the equation. Soc Sci Med. 2018;201:103-10. | 4 |
| Do DP. The dynamics of income and neighborhood context for population health: do long-term measures of socioeconomic status explain more of the black/white health disparity than single-point-in-time measures? Soc Sci Med. 2009;68(8):1368-75. | 2 |
| Do DP, Finch BK. The link between neighborhood poverty and health: context or composition? Am J Epidemiol. 2008;168(6):611-9. | 2 |
| do Rosario Giraldes M. Equity in socioeconomic areas with impact on health in countries of European Union. [Portuguese]. Cadernos de saude publica. 2001;17(3):533-44. | 6 |
| Dorling D, Mitchell R, Pearce J. The global impact of income inequality on health by age: an observational study. BMJ. 2007;335(7625):873. | 1 |
| Dowling PT, Allevato JM. Inequality in income and mortality in US - Lower mortality in Hispanic population may have affected findings. BMJ. 1996;313(7052):301-. | 4 |
| Drabo A. Impact of income inequality on health: does environment quality matter? Environment and Planning a-Economy and Space. 2011;43(1):146-65. | 3 |
| Drukker M, Feron FJM, van Os J. Income inequality at neighbourhood level and quality of life - A contextual analysis. Social Psychiatry and Psychiatric Epidemiol. 2004;39(6):457-63. | 3 |
| Duleep HO. Mortality and Income Inequality among Economically Developed Countries. Social Security Bulletin. 1995;58(2):34-50. | 5 |
| Dunn JR, Burgess B, Ross NA. Income distribution, public services expenditures, and all cause mortality in US States. J Epidemiol Community Health. 2005;59(9):768-74. | 5 |
| Dunn JR, Schaub P, Ross NA. Unpacking income inequality and population health - The peculiar absence of geography. Canadian J Public Health. 2007;98:S10-S7. | 4 |
| Eckersley R. Beyond inequality: Acknowledging the complexity of social determinants of health. Soc Sci Med. 2015;147:121-5. | 4 |
|  |  |
| Elgar FJ. Income inequality, trust, and population health in 33 countries. Am J Public Health. 2010;100(11):2311-5. | 5 |
| Ellison GT. Income inequality, social trust, and self-reported health status in high-income countries. Ann the New York Academy of Sciences. 1999;896:325-8. | 4 |
| Elstad JI, Dahl E, Hofoss D. [Skewed income distribution and geographical mortality differences]. Tidsskr Nor Laegeforen. 2005;125(22):3082-4. | 6 |
| Elstad JI, Dahl E, Hofoss D. Associations between relative income and mortality in Norway: a register-based study. Eur J Public Health. 2006;16(6):640-4. | 2 |
| Esmaeili A, Mansouri S, Moshavash M. Income inequality and population health in Islamic countries. Public Health. 2011;125(9):577-84. | 4 |
| Fan CJ, Ouyang W, Tian L, Song Y, Miao WS. Elderly Health Inequality in China and its Determinants: A Geographical Perspective. Int J Environmental Res and Public Health. 2019;16(16). | 2 |
| Fang P, Dong S, Xiao J, Liu C, Feng X, Wang Y. Regional inequality in health and its determinants: evidence from China. Health Policy. 2010;94(1):14-25. | 4 |
| Ferre JC. Economic Inequalities in Latin America at the Base of Adverse Health Indicators. Int J Social Determinants of Health and Health Serv. 2016;46(3):501-22. | 4 |
| Forster DP. Income distribution and life expectancy. BMJ. 1992;304(6828):715-6. | 4 |
| Franca MC, Paes NA. Income, income inequality and mortality in metropolitan regions of Brazil: an exploratory approach. Papeles De Poblacion. 2007;13(53):225-39. | 4 |
| Franzini L, Giannoni M. Determinants of health disparities between Italian regions. BMC Public Health. 2010;10:296. | 2 |
| Franzini L, Ribble J, Spears W. The effects of income inequality and income level on mortality vary by population size in Texas counties. J Health and Social Behavior. 2001;42(4):373-87. | 4 |
| Fukuda Y, Nakamura K, Takano T. Wide range of socioeconomic factors associated with mortality among cities in Japan. Health Promotion Int. 2004;19(2):177-87. | 2 |
| Furnee CA, Pfann GA. Individual vulnerability and the nurturing state: the case of self-reported health and relative income. Soc Sci Med. 2010;1(1):125-33. | 4 |
| Galea S, Ahern J, Karpati A. A model of underlying socioeconomic vulnerability in human populations: evidence from variability in population health and implications for public health. Soc Sci Med. 2005;60(11):2417-30. | 3 |
| Gavurová B, Kováč V, Šoltés M, Kot S, Majerník J. Income Inequality in Non-communicable Diseases Mortality among the Regions of the Slovak Republic. Central Eur J Public Health. 2017;25:S31-S6. | 3 |
| Gero K, Miyawaki A, Kawachi I. Relative Income Deprivation and All-Cause Mortality in Japan: Do Life Priorities Matter? Ann Behavioral Medicine. 2020;54(9):665-79. | 2 |
| Gero K, Yazawa A, Kondo N, Hanazato M, Kondo K, Kawachi I. Comparison of three indices of relative income deprivation in predicting health status. Soc Sci Med. 2022;294:114722. | 2 |
| Ghosal RK. Globalisation and Inequality. Indian Economic Journal. 2005;53(2):71-86. | 4 |
| Giordano GN, Mewes J, Miething A. Trust and all-cause mortality: a multilevel study of US General Social Survey data (1978-2010). J Epidemiol Community Health. 2019;73(1):50-5. | 2 |
| Goldman N. Social inequalities in health: Disentangling the underlying mechanisms. Ann New York Academy of Sciences. 2001;954:118-39. | 4 |
| Gravelle H. Mortality and distribution of income - Widening income inequalities cause poorer health - Reply. BMJ. 1998;316(7144):1612-. | 4 |
| Gravelle H. How much of the relation between population mortality and unequal distribution of income is a statistical artefact? BMJ. 1998;316(7128):382-5. | 4 |
| Gravelle H, Sutton M. Income related inequalities in self assessed health in Britain: 1979--1995. J Epidemiol Community Health. 2003;57(2):125-9. | 2 |
| Gravelle H, Wildman J, Sutton M. Income, income inequality and health: what can we learn from aggregate data? Soc Sci Med. 2002;54(4):577-89. | 4 |
| Hajebi E, Razmi MJ. Effect of Income Inequality on Health Status in a Selection of Middle and Low Income Countries. Equilibrium: Quarterly J Economics and Economic Policy. 2014;9(4):133-52. | 4 |
| Hamilton TG, Kawachi I. Changes in income inequality and the health of immigrants. Soc Sci Med. 2013;80:57-66. | 2 |
| Hao Y, Liu JH, Lu ZN, Shi RJ, Wu HT. Impact of income inequality and fiscal decentralization on public health: Evidence from China. Economic Modelling. 2021;94:934-44. | 3 |
| He Y, Zhou L, Li J, Wu J. An empirical analysis of the impact of income inequality and social capital on physical and mental health - take China’s micro-database analysis as an example. Int J Equity Health. 2021;20(1):241. | 2 |
| Henriksson G, Weitoft GR, Allebeck P. Associations between income inequality at municipality level and health depend on context - a multilevel analysis on myocardial infarction in Sweden. Soc Sci Med. 2010;71(6):1141-9. | 3 |
| Herzer D, Nunnenkamp P. Income Inequality and Health: Evidence from Developed and Developing Countries. Economics: The Open-Access. 2015;9:1-57. | 4 |
| Hill TD, Jorgenson A. Bring out your dead!: A study of income inequality and life expectancy in the United States, 2000--2010. Health Place. 2018;49:1-6. | 5 |
| Hill TD, Jorgenson AK, Ore P, Balistreri KS, Clark B. Air quality and life expectancy in the United States: An analysis of the moderating effect of income inequality. Soc Sci Med - Population Health. 2019;7. | 2 |
| Hoffmann R, Hu YN, de Gelder R, Menvielle G, Bopp M, Mackenbach JP. The impact of increasing income inequalities on educational inequalities in mortality - An analysis of six European countries. Int J Equity in Health. 2016;15. | 4 |
| Hong E, Ahn BC. Income-related health inequalities across regions in Korea. Int J Equity in Health. 2011;10. | 2 |
| Houghton F. Income inequality and health in contemporary Ireland. Irish J Medical Science. 2002;171(4):235-6. | 4 |
| Houweling TAJ, Kunst AE, Mackenbach JP. World Health Report 2000: Inequality index and socioeconomic inequalities in mortality. Lancet. 2001;357(9269):1671-2. | 4 |
| Hu Y, van Lenthe FJ, Mackenbach JP. Income inequality, life expectancy and cause-specific mortality in 43 European countries, 1987--2008: a fixed effects study. Eur J Epidemiol. 2015;30(8):615-25. | 5 |
| Huijts T, Eikemo TA, Skalicka V. Income-related health inequalities in the Nordic countries: examining the role of education, occupational class, and age. Soc Sci Med. 2010;71(11):1964-72. | 2 |
| Ickert C, Senthilselvan A, Jhangri GS. Multilevel Modeling of Health Inequalities at the Intersection of Multiple Social Identities in Canada. The Sociological Q. 2021;63(2):214-46. | 2 |
| Idrovo AJ. [Income inequality, corruption, and life expectancy at birth in Mexico]. Revista de Salud Pública. 2005;7(2):121-9. | 6 |
| Idrovo AJ, Ruiz-Rodriguez M, Manzano-Patino AP. Beyond the income inequality hypothesis and human health: a worldwide exploration. Revista de Salud Pública. 2010;44(4):695-702. | 4 |
| Inoue Y, Umezaki M, Watanabe C. Emergence of income inequality and its impact on subjective quality of life in an ethnic minority community in Hainan Island, China. Anthropological Science. 2012;120(1):51-60. | 2 |
| Islam MS, Mondal MNI, Tareque MI, Rahman MA, Hoque MN, Ahmed MM, et al. Correlates of healthy life expectancy in low- and lower-middle-income countries. BMC Public Health. 2018;18(1):476. | 2 |
| Jaeggi AV, Blackwell AD, von Rueden C, Trumble BC, Stieglitz J, Garcia AR, et al. Do wealth and inequality associate with health in a small-scale subsistence society? Elife. 2021;10(05):14. | 5 |
| Japaridze I, Sayour N. Dying from envy: The role of inequality. Health Econ. 2021;30(6):1374-92. | 2 |
| Jen MH, Jones K, Johnston R. Compositional and contextual approaches to the study of health behaviour and outcomes: Using multi-level modelling to evaluate Wilkinson’s income inequality hypothesis. Health Place. 2009;15(1):198-203. | 4 |
| Johnston R, Jen MH, Jones K. Regional Variations in Voting at British General Elections, 1950–2001: Group-Based Latent Trajectory Analysis. Health Place. 2009;15(4):1163-5. | 4 |
| Judge K. Income distribution and life expectancy: a critical appraisal. BMJ. 1995;311(7015):1282-5. | 4 |
| Judge K, Mulligan JA, Benzeval M. Income inequality and population health. Soc Sci Med. 1998;46(4-5):567-79. | 5 |
| Judge K, Mulligan JA, Benzeval M. The relationship between income inequality and population health. Soc Sci Med. 1998;47(7):983-5. | 4 |
| Jutz R. The role of income inequality and social policies on income-related health inequalities in Europe. Int J Equity in Health. 2015;14(1). | 4 |
| Kanazawa S. Mind the gap… in intelligence: re-examining the relationship between inequality and health. British J Health Psychology. 2006;11(Pt 4):623-42. | 4 |
| Kaplan GA. Inequality in income and mortality in the United States: Analysis of mortality and potential pathways. BMJ. 1996;312(7041):1253-. | 4 |
| Karlsdotter K, Martin Martin JJ, Lopez del Amo Gonzalez Mdel P. [Influence of income, income inequalities and social capital on the health of persons aged 65 and over in Spain in 2007]. Gaceta Sanitaria. 2011;25 Suppl 2:59-65. | 6 |
| Karlsson M, Nilsson T, Lyttkens CH, Leeson G. Income inequality and health: importance of a cross-country perspective. Soc Sci Med. 2010;70(6):875-85. | 4 |
| Karpati AM, Bassett MT, McCord C. Neighbourhood mortality inequalities in New York City, 1989--1991 and 1999--2001. J Epidemiol Community Health. 2006;60(12):1060-4. | 2 |
| Kawachi I. Income inequality and economic residential segregation. J Epidemiol Community Health. 2002;56(3):165-6. | 4 |
| Kawachi I, Kennedy BP. Health and social cohesion: why care about income inequality? BMJ. 1997;314(7086):1037-40. | 4 |
| Kawachi I, Kennedy BP. The relationship of income inequality to mortality: does the choice of indicator matter? Soc Sci Med. 1997;45(7):1121-7. | 4 |
| Kawachi I, Kennedy BP. Income inequality and health: pathways and mechanisms. Health Serv Res. 1999;34(1 Pt 2):215-27. | 4 |
| Kawachi I, Kennedy BP, Lochner K, ProthrowStith D. Social capital, income inequality, and mortality. Am J Public Health. 1997;87(9):1491-8. | 5 |
| Kennedy BP, Kawachi I, ProthrowStith D. Income distribution and mortality: Cross sectional ecological study of the Robin Hood index in the United States. BMJ. 1996;312(7037):1004-7. | 3 |
| Khang YH, Kim HR. Socioeconomic Inequality in mortality using 12-year follow-up data from nationally representative surveys in South Korea. Int J Epidemiol. 2005;34(3):630-7. | 2 |
| Khang YH, Lim D, Bahk J, Kim I, Kang HY, Chang Y, et al. A publicly well-accepted measure versus an academically desirable measure of health inequality: cross-sectional comparison of the difference between income quintiles with the slope index of inequality. BMJ. 2019;9(6):e028687. | 2 |
| Khang YH, Yang S, Cho HJ, Jung-Choi K, Yun SC. Decomposition of socio-economic differences in life expectancy at birth by age and cause of death among 4 million South Korean public servants and their dependents. Int J Epidemiol. 2010;39(6):1656-66. | 2 |
| Khullar D, Fisher J, Chandra A. Trickle-down innovation and the longevity of nations. Lancet. 2019;393(10187):2272-4. | 4 |
| Kibele EUB, Jasilionis D, Shkolnikov VM. Widening socioeconomic differences in mortality among men aged 65 years and older in Germany. J Epidemiol Community Health. 2013;67(5):453-7. | 2 |
| Kim D. Do USA state-level social spending and income inequality predict individual mortality? A fixed-effects, instrumental variable analysis. J Epidemiol Community Health. 2011;65:A119-A. | 4 |
| Kim HH, Katelyn Kim HJ. Income inequality, emotional anxiety, and self-rated health in times of the coronavirus pandemic: Evidence from a cross-national survey. Res Soc Stratif Mobil. 2021;75:100640. | 2 |
| Kim J. Is Regional Income Inequality Associated With the Individual Health of Older Adults? Evidence From the Health and Retirement Study. Poverty Public Policy. 2016;8(4):416-29. | 3 |
| Kim K-t. Which Income Inequality Influences Which Health Indicators? Analysis of the Income Inequality Hypothesis with Market and Disposable Gini Indicators. Social Indicators Res. 2019;146(3):473-85. | 4 |
| Kim KT. The relationships between income inequality, welfare regimes and aggregate health: a systematic review. Eur J Public Health. 2017;27(3):397-404. | 3 |
| Kim KT. Income inequality, welfare regimes and aggregate health: Review of reviews. Int J Social Welfare. 2019;28(1):31-43. | 3 |
| Kim KT. Revisiting the Income Inequality Hypothesis With 292 OECD Regional Units. Int J Social Determinants of Health and Health Serv. 2019;49(2):360-70. | 4 |
| Koivusilta LK. Country-Level Investment in Cultural Opportunity Structures. A Potential Source of Health Differences Between 21 European Countries. Social Indicators Res. 2018;137(3):1091-118. | 4 |
| Kondo N. Socioeconomic disparities and health: impacts and pathways. J Epidemiol. 2012;22(1):2-6. | 4 |
| Kondo N, Sembajwe G, Kawachi I, van Dam RM, Subramanian SV, Yamagata Z. Income inequality, mortality, and self rated health: meta-analysis of multilevel studies. BMJ. 2009;339. | 4 |
| Kondo N, Kawachi I, Subramanian SV, Takeda Y, Yamagata Z. Do social comparisons explain the association between income inequality and health?: Relative deprivation and perceived health among male and female Japanese individuals. Soc Sci Med. 2008;67(6):982-7. | 2 |
| Kondo N, van Dam RM, Sembajwe G, Subramanian SV, Kawachi I, Yamagata Z. Income inequality and health: the role of population size, inequality threshold, period effects and lag effects. J Epidemiol Community Health. 2012;66(6):e11. | 4 |
| Kunitz SJ, Pesis-Katz I. Mortality of white Americans, African Americans, and Canadians: the causes and consequences for health of welfare state institutions and policies. Milbank Q. 2005;83(1):5-39. | 4 |
| Kunst AE, Bos V, Lahelma E, Bartley M, Lissau I, Regidor E, et al. Trends in socioeconomic inequalities in self-assessed health in 10 European countries. Int J Epidemiol. 2005;34(2):295-305. | 2 |
| Kunst AE, Geurts JJ, van den Berg J. International variation in socioeconomic inequalities in self reported health. J Epidemiol Community Health. 1995;49(2):117-23. | 2 |
| Kuo CT, Chen DR. Double disadvantage: income inequality, spatial polarization and mortality rates in Taiwan. J Public Health. 2018;40(3):e228-e34. | 4 |
| Kuo CT, Chiang TL. The association between relative deprivation and self-rated health, depressive symptoms, and smoking behavior in Taiwan. Soc Sci Med. 2013;89:39-44. | 2 |
| Lago S, Cantarero D, Rivera B, Pascual M, Blazquez-Fernandez C, Casal B, et al. Socioeconomic status, health inequalities and non-communicable diseases: a systematic review. J Public Health-Heidelberg. 2018;26(1):1-14. | 4 |
| Lai DJ, Huang J, Risser JM, Kapadia AS. Statistical properties of generalized Gini coefficient with application to health inequality measurement. Social Indicators Res. 2008;87(2):249-58. | 4 |
| Lakshman R, McConville A, How S, Flowers J, Wareham N, Cosford P. Association between area-level socioeconomic deprivation and a cluster of behavioural risk factors: cross-sectional, population-based study. J Public Health. 2011;33(2):234-45. | 2 |
| Lange-Maia BS, De Maio F, Avery EF, Lynch EB, Laflamme EM, Ansell DA, et al. Association of community-level inequities and premature mortality: Chicago, 2011--2015. J Epidemiol Community Health. 2018;72(12):1099-103. | 2 |
| Laporte A. A note on the use of a single inequality index in testing the effect of income distribution on mortality. Soc Sci Med. 2002;55(9):1561-70. | 4 |
| Laporte A, Ferguson BS. Income inequality and mortality: time series evidence from Canada. Health Policy. 2003;66(1):107-17. | 4 |
| Lau EW, Schooling CM, Tin KY, Leung GM. Income inequality and cause-specific mortality during economic development. Ann Epidemiol. 2012;22(4):285-94. | 5 |
| Lawrence C. The effects of income inequality on health. Australian Health Review. 1999;22(1):97-106. | 4 |
| Leigh A, Jencks C. Inequality and Mortality: Long-Run Evidence from a Panel of Countries. J Health Econ. 2007;26(1):1-24. | 4 |
| Lewer D, Jayatunga W, Aldridge RW, Edge C, Marmot M, Story A, et al. Premature mortality attributable to socioeconomic inequality in England between 2003 and 2018: an observational study. Lancet Public Health. 2020;5(1):e33-e41. | 2 |
| Lim Y, Kim H, Kim M, Cho Y. Income Inequality as an Effect Modifier of the Association Between PM10 and Self-Rated Health-a Multi-Level Study. Epidemiol. 2008;19(6):S276-S. | 2 |
| Liu T, Dutton DJ. With great inequality comes great responsibility: the role of government spending on population health in the presence of changing income distributions. Can J Public Health. 2020;21. | 4 |
| Liu T, Dutton DJ. With great inequality comes great responsibility: the role of government spending on population health in the presence of changing income distributions. Can J Public Health. 2021;112(2):199-209. | 2 |
| Lobmayer P, Wilkinson R. Income, inequality and mortality in 14 developed countries. Sociol Health Illness. 2000;22(4):401-14. | 2 |
| Lobmayer P, Wilkinson RG. Inequality, residential segregation by income, and mortality in US cities. J Epidemiol Community Health. 2002;56(3):183-7. | 4 |
| Lundberg O, Fritzell J. Income distribution, income change and health: on the importance of absolute and relative income for health status in Sweden. WHO Regional Publications Eur Series. 1994;54:37-58; discussion 123. | 4 |
| Luo W, Xie Y. Economic growth, income inequality and life expectancy in China. Soc Sci Med. 2020;256 (no pagination). | 4 |
| Lynch J. Income inequality and health: expanding the debate. Soc Sci Med. 2000;51(7):1001-5; discussion 9. | 4 |
| Lynch J, Harper S, Davey Smith G. Commentary: Plugging leaks and repelling boarders--where to next for the SS income inequality? Int J Epidemiol. 2003;32(6):1029-36; discussion 37. | 4 |
| Lynch J, Harper S, Kaplan GA, Davey Smith G. Associations between income inequality and mortality among US states: the importance of time period and source of income data. Am J Public Health. 2005;95(8):1424-30. | 5 |
| Lynch J, Smith GD. Commentary: Income inequality and health: the end of the story? Int J Epidemiol. 2002;31(3):549-51. | 4 |
| Lynch J, Smith GD, Harper S, Hillemeier M. Is income inequality a determinant of population health? Part 2. U.S. National and regional trends in income inequality and age- and cause-specific mortality. Milbank Q. 2004;82(2):355-400. | 4 |
| Lynch J, Smith GD, Harper S, Hillemeier M, Ross N, Kaplan GA, et al. Is income inequality a determinant of population health? Part 1. A systematic review. Milbank Q. 2004;82(1):5-99. | 4 |
| Lynch J, Smith GD, Hillemeier M, Shaw M, Raghunathan T, Kaplan G. Income inequality, the psychosocial environment, and health: comparisons of wealthy nations. Lancet. 2001;358(9277):194-200. | 5 |
| Lynch JW, Kaplan GA. Understanding how inequality in the distribution of income affects health. J Health Psychology. 1997;2(3):297-314. | 4 |
| Lynch JW, Kaplan GA, Pamuk ER, Cohen RD, Heck KE, Balfour JL, et al. Income inequality and mortality in metropolitan areas of the United States. Am J Public Health. 1998;88(7):1074-80. | 4 |
| Lynch JW, Smith GD, Kaplan GA, House JS. Income inequality and mortality: importance to health of individual income, psychosocial environment, or material conditions. BMJ. 2000;320(7243):1200-4. | 4 |
| Macinko JA, Shi L, Starfield B, Wulu JT, Jr. Income inequality and health: a critical review of the literature. Medical Care Res and Review. 2003;60(4):407-52. | 4 |
| Mackenbach JP. Income inequality and population health - Evidence favouring a negative correlation between income inequality and life expectancy has disappeared. BMJ. 2002;324(7328):1-2. | 4 |
| Mackenbach JP. Convergence and divergence of life expectancy in Europe: a centennial view. Eur J Epidemiol. 2013;28(3):229-40. | 2 |
| Mackenbach JP, Looman CW. Changing patterns of mortality in 25 European countries and their economic and political correlates, 1955--1989. Int J Public Health. 2013;58(6):811-23. | 2 |
| Mackenbach JP, Rubio Valverde J, Bopp M, Bronnum-Hansen H, Costa G, Deboosere P, et al. Progress against inequalities in mortality: register-based study of 15 European countries between 1990 and 2015. Eur J Epidemiol. 2019;34(12):1131-42. | 4 |
| Malmstrom M, Johansson SE, Sundquist J. A hierarchical analysis of long-term illness and mortality in socially deprived areas. Soc Sci Med. 2001;53(3):265-75. | 2 |
| Manz KM, Mansmann U. Inequality indices to monitor geographic differences in incidence, mortality and fatality rates over time during the COVID-19 pandemic. PLoS One. 2021;16(5):e0251366. | 3 |
| Marmot M. Income inequality, social environment, and inequalities in health. J Policy Analysis Management. 2001;20(1):156-9. | 4 |
| Martikainen P, Valkonen T. Inequalities in health. Policies to reduce income inequalities are unlikely to eradicate inequalities in mortality. BMJ. 1999;319(7205):319. | 4 |
| Maskileyson D. Health trajectories of immigrants in the United States: Does income inequality of country of origin matter? Soc Sci Med. 2019;230:246-55. | 2 |
| Massey P, Durrheim D. Income inequality and health status: a nursing issue. Australian J Advanced Nursing. 2007;25(2):84-8. | 4 |
| Materia E, Cacciani L, Bugarini G, Cesaroni G, Davoli M, Mirale MP, et al. Income inequality and mortality in Italy. Eur J Public Health. 2005;15(4):411-7. | 4 |
| Matthew P, Brodersen DM. Income inequality and health outcomes in the United States: An empirical analysis. Social Science Journal. 2018;55(4):432-42. | 3 |
| Mayrhofer T, Schmitz H. Testing the Relationship between Income Inequality and Life Expectancy: A Simple Correction for the Aggregation Effect When Using Aggregated Data. J Population Econ. 2014;27(3):841-56. | 4 |
| McCartney G, Hearty W, Arnot J, Popham F, Cumbers A, McMaster R. Impact of Political Economy on Population Health: A Systematic Review of Reviews. Am J Public Health. 2019;109(6):e1-e12. | 4 |
| McGrail KM, van Doorslaer E, Ross NA, Sanmartin C. Income-related health inequalities in Canada and the United States: a decomposition analysis. Am J Public Health. 2009;99(10):1856-63. | 2 |
| McIsaac SJ, Wilkinson RG. Income distribution and cause-specific mortality. Eur J Public Health. 1997;7(1):45-53. | 4 |
| McLaughlin DK, Stokes CS. Income inequality and mortality in US counties: does minority racial concentration matter? Am J Public Health. 2002;92(1):99-104. | 4 |
| McLaughlin DK, Stokes CS, Nonoyama A. Residence and income inequality: Effects on mortality among US counties. Rural Sociol. 2001;66(4):579-98. | 4 |
| Meijer M, Rohl J, Bloomfield K, Grittner U. Do neighborhoods affect individual mortality? A systematic review and meta-analysis of multilevel studies. Soc Sci Med. 2012;74(8):1204-12. | 2 |
| Melix BL, Uejio CK, Kintziger KW, Reid K, Duclos C, Jordan MM, et al. Florida neighborhood analysis of social determinants and their relationship to life expectancy. BMC Public Health. 2020;20(1):632. | 4 |
| Mellor JM, Milyo J. Reexamining the evidence of an ecological association between income inequality and health. J Health Politics, Policy and Law. 2001;26(3):487-522. | 4 |
| Mellor JM, Milyo J. Exploring the relationships between income inequality, socioeconomic status and health: a self-guided tour? Int J Epidemiol. 2002;31(3):685-7. | 4 |
| Mellor JM, Milyo J. On the Use of Age-Adjusted Mortality Rates in Studies of Income Inequality and Population Health. J Health Politics, Policy and Law. 2002;27(2):293-6. | 4 |
| Mellor JM, Milyo JD. Income inequality and health. J Policy Analysis Management. 2001;20(1):151-5. | 4 |
| Messias E. Income inequality, illiteracy rate, and life expectancy in Brazil. Am J Public Health. 2003;93(8):1294-6. | 4 |
| Messias E. Income inequality, death, and depression: An ecological analysis of US States. Eur Psychiatry. 2012;27(SUPPL. 1). | 4 |
| Mfenyana K, Griffin M, Yogeswaran P, Modell B, Modell M, Chandia J, et al. Socio-economic inequalities as a predictor of health in South Africa - The Yenza cross-sectional study. South African Med J. 2006;96(4):323-30. | 2 |
| Milyo J, Kennedy BP, Kawachi I, Glass R, Prothrow-Stith D. Income distribution, socioeconomic status, and self rated health in US (multiple letters). BMJ. 1999;318(7195):1417-8. | 4 |
| Milyo J, Parnerkar I. Income Inequality, Social Capital and Mortality: Re-examining the State-Level Data. Harris School of Public Policy Studies, University of Chicago, Working Papers: 0309; 2003. | 1 |
| Milyo JD, Mellor JM. Is inequality bad for our health? Critical Review. 1999;13(3-4):359-72. | 4 |
| Mode NA, Evans MK, Zonderman AB. Race, Neighborhood Economic Status, Income Inequality and Mortality. PLoS One. 2016;11(5). | 4 |
| Moore EG, Pacey MA. Changing Income Inequality and the Elderly in Canada 1991--1996: Provincial Metropolitan and Local Dimensions. McMaster University, Social and Economic Dimensions of an Aging Population Research Papers; 2001. | 1 |
| Moore S. Peripherality, income inequality, and life expectancy: revisiting the income inequality hypothesis. Int J Epidemiol. 2006;35(3):623-32. | 4 |
| Mortensen LH, Rehnberg J, Dahl E, Diderichsen F, Elstad JI, Martikainen P, et al. Shape of the association between income and mortality: a cohort study of Denmark, Finland, Norway and Sweden in 1995 and 2003. BMJ. 2016;6(12):e010974. | 4 |
| Muennig P. Redistribution and health. Am J Public Health. 2005;95(8):1306; -7. | 4 |
| Muller A. Education, income inequality, and mortality: a multiple regression analysis. BMJ. 2002;324(7328):23-5. | 4 |
| Muller A. Association between income inequality and mortality among US States: Considering population at risk. Am J Public Health. 2006;96(4):590-1. | 4 |
| Muntaner C, Chung H. Commentary: macrosocial determinants, epidemiology, and health policy: should politics and economics be banned from social determinants of health research? J Public Health Policy. 2008;29(3):299-306. | 4 |
| Muntaner C, Lynch J. Income inequality, social cohesion, and class relations: A critique of Wilkinson’s neo-Durkheimian research program. Int J Health Serv. 1999;29(1):59-81. | 4 |
| Muramatsu N. County-level income inequality and depression among older Americans. Health Serv Res. 2003;38(6):1863-83. | 3 |
| Nakaya T, Dorling D. Geographical inequalities of mortality by income in two developed island countries: a cross-national comparison of Britain and Japan. Soc Sci Med. 2005;60(12):2865-75. | 4 |
| Naumova EN, Cohen SA. Commentary: population-level risk factors, population health, and health policy. J Public Health Policy. 2008;29(3):290-8. | 4 |
| Navarro V, Borrell C, Benach J, Muntaner C, Quiroga A, Rodriguez-Sanz M, et al. The importance of the political and the social in explaining mortality differentials among the countries of the OECD, 1950--1998. Int J Social Determinants of Health and Health Serv. 2003;33(3):419-94. | 4 |
| Neumayer E, Plumper T. Inequalities of Income and Inequalities of Longevity: A Cross-Country Study. Am J Public Health. 2016;106(1):160-5. | 4 |
| Nowatzki NR. Wealth inequality and health: a political economy perspective. Int J Social Determinants of Health and Health Serv. 2012;42(3):403-24. | 4 |
| Nuru-Jeter AM, LaVeist TA. Racial segregation, income inequality, and mortality in US metropolitan areas. J Urban Health. 2011;88(2):270-82. | 4 |
| Nuru-Jeter AM, Williams T, LaVeist TA. Distinguishing the race-specific effects of income inequality and mortality in U.S. metropolitan areas. Int J Social Determinants of Health and Health Serv. 2014;44(3):435-56. | 4 |
| O'Donnell MP. Erosion of our moral compass, social trust, and the fiscal strength of the United States: income inequality, tax policy, and well-being. Am J Health Promotion. 2012;26(4):iv-xi. | 4 |
| Odusanya IA, Akinlo AE. Income Inequality and Population Health in Sub-Saharan Africa: A Test of Income Inequality-Health Hypothesis. J Population and Social Studies. 2021;29:235-54. | 5 |
| Okulicz-Kozaryn A. Income Inequality and Wellbeing. App Res in Quality of Life. 2015;10(3):405-18. | 3 |
| Omer AS, Bezruchka S, Longhi D, Kelly Z, Brown M, Hagopian A. The effects of household assets inequality and conflict on population health in Sudan. Ann Global Health. 2015;81 (1):116. | 4 |
| Orji A, Okechukwu E. Income, Income Distribution and Health Outcomes in Nigeria: Empirical Evidence from National Demographic and Health Surveys. Nigerian J Economic and Social Studies. 2015;57(1):101-50. | 3 |
| Oronce CIA, Scannell CA, Kawachi I, Tsugawa Y. Association Between State-Level Income Inequality and COVID-19 Cases and Mortality in the USA. J Gen Intern Med. 2020;35(9):2791-3. | 3 |
| Oshio T, Kobayashi M. Area-Level Income Inequality and Individual Happiness: Evidence from Japan. J Happiness Studies. 2011;12(4):633-49. | 3 |
| Oshio T, Urakawa K. The Association Between Perceived Income Inequality and Subjective Well-being: Evidence from a Social Survey in Japan. Social Indicators Res. 2014;116(3):755-70. | 2 |
| Park J, Ryu SY, Han MA, Choi SW. The association between income inequality and all-cause mortality across urban communities in Korea. BMC Public Health. 2015;15:574. | 4 |
| Pascual M, Cantarero D, Sarabia JM. Income Inequality and Health: Do the Equivalence Scales Matter? Atlantic Economic Journal. 2005;33(2):169-78. | 5 |
| Paul S. Income Inequality and Individual Health Status: Evidence from India. J Quantitative Econ. 2021. | 3 |
| Pickett KE, Pearl M. Multilevel analyses of neighbourhood socioeconomic context and health outcomes: a critical review. J Epidemiol Community Health. 2001;55(2):111-22. | 4 |
| Pickett KE, Wilkinson RG. Income inequality and health: a causal review. Soc Sci Med. 2015;128:316-26. | 4 |
| Pocas A, Soukiazis E, Antunes M. Factors Explaining Life Expectancy at Age 65: A Panel Data Approach Applied to European Union Countries. Social Indicators Res. 2020;150(1):265-88. | 2 |
| Pop IA, van Ingen E, van Oorschot W. Inequality, Wealth and Health: Is Decreasing Income Inequality the Key to Create Healthier Societies? Social Indicators Res. 2013;113(3):1025-43. | 4 |
| Porta M, Borrell C, Copete JL. Commentary: Theory in the fabric of evidence on the health effects of inequalities in income distribution. Int J Epidemiol. 2002;31(3):543-6. | 4 |
| Prag P, Mills M, Wittek R. Income and Income Inequality as Social Determinants of Health: Do Social Comparisons Play a Role? Eur Sociological Review. 2014;30(2):218-29. | 4 |
| Prus SG, Brown RL. Age-specific Income Inequality and Life Expectancy: New Evidence. McMaster University, Social and Economic Dimensions of an Aging Population Research Papers; 2008. | 1 |
| Puterman E, Weiss J, Hives BA, Gemmill A, Karasek D, Mendes WB, et al. Predicting mortality from 57 economic, behavioral, social, and psychological factors. Proceedings of the National Academy of Sciences of the United States of America. 2020;117(28):16273-82. | 2 |
| Qin W, Xu L, Wu S, Shao H. Income, Relative Deprivation and the Self-Rated Health of Older People in Urban and Rural China. Front Public Health. 2021;9:658649. | 2 |
| Rahkonen O, Arber S, Lahelma E, Martikainen P, Silventoinen K. Understanding income inequalities in health among men and women in Britain and Finland. Int J Health Serv. 2000;30(1):27-47. | 4 |
| Ram R. Income inequality, poverty, and population health: evidence from recent data for the United States. Soc Sci Med. 2005;61(12):2568-76. | 4 |
| Rambotti S. Recalibrating the spirit level: An analysis of the interaction of income inequality and poverty and its effect on health. Soc Sci Med. 2015;139:123-31. | 4 |
| Rasella D, Aquino R, Barreto ML. Impact of income inequality on life expectancy in a highly unequal developing country: the case of Brazil. J Epidemiol Community Health. 2013;67(8):661-6. | 4 |
| Razum O. Income inequality and mortality in Canada and the United States - Low mortality in Canadian cities may be driven by low mortality in immigrants. BMJ. 2000;321(7275):1533-. | 4 |
| Rebeira M, Grootendorst P, Coyte PC, Aguirregabiria V. Does Rising Income Inequality Affect Mortality Rates in Advanced Economies? Economics: The Open-Access. 2017;11. | 4 |
| Regidor E, Calle ME, Navarro P, Dominguez V. Trends in the association between average income, poverty and income inequality and life expectancy in Spain. Soc Sci Med. 2003;56(5):961-71. | 4 |
| Regidor E, Martinez D, Astasio P, Ortega P, Calle ME, Dominguez V. Trends of socioeconomic inequalities and socioeconomic inequalities in self-perceived health in Spain. Gaceta Sanitaria. 2006;20(3):178-82. | 2 |
| Regidor E, Ronda E, Pascual C, Martinez D, Calle ME, Dominguez V. Decreasing socioeconomic inequalities and increasing health inequalities in Spain: a case study. Am J Public Health. 2006;96(1):102-8. | 2 |
| Regidor E, Santos JM, Ortega P, Calle ME, Astasio P, Martinez D. Decreasing income inequality and emergence of the association between income and premature mortality: Spain, 1970--2010. Health & Place. 2014;27:30-7. | 4 |
| Reijneveld SA, Verheij RA, de Bakker DH. Relative importance of urbanicity, ethnicity and socioeconomic factors regarding area mortality differences. J Epidemiol Community Health. 1999;53(7):444-5. | 2 |
| Rodgers GB. Income and inequality as determinants of mortality: an international cross-section analysis. 1979. Int J Epidemiol. 2002;31(3):533-8. | 4 |
| Rodriguez Garcia J. [Socioeconomic inequality and its association with mortality indicators in the departments of Colombia in 2000]. Revista Panamericana de Salud Pública. 2007;21(2-3):111-24. | 6 |
| Ronzio CR. Urban premature mortality in the U.S. between 1980 and 1990: changing roles of income inequality and social spending. J Public Health Policy. 2003;24(3-4):386-400. | 4 |
| Ronzio CR, Pamuk E, Squires GD. The politics of preventable deaths: local spending, income inequality, and premature mortality in US cities. J Epidemiol Community Health. 2004;58(3):175-9. | 4 |
| Roos LL, Magoon J, Gupta S, Chateau D, Veugelers PJ. Socioeconomic determinants of mortality in two Canadian provinces: multilevel modelling and neighborhood context. Soc Sci Med. 2004;59(7):1435-47. | 2 |
| Rose SM, Hatzenbuehler S. Embodying social class The link between poverty, income inequality and health. Int Social Work. 2009;52(4):459-+. | 4 |
| Ross NA, Dorling D, Dunn JR, Henriksson G, Glover J, Lynch J, et al. Metropolitan-income inequality and working-age mortality: A cross-sectional analysis using comparable data from five countries. J Urban Health. 2005;82(1):101-10. | 4 |
| Ross NA, Lynch J. Commentary: the contingencies of income inequality and health: reflections on the Canadian Experience. Int J Epidemiol. 2004;33(2):318-9. | 4 |
| Ross NA, Wolfson MC. Income inequality and mortality in Canada and the United States. An analysis of provinces/states. Ann the New York Academy of Sciences. 1999;896:338-40. | 4 |
| Ross NA, Wolfson MC, Dunn JR, Berthelot JM, Kaplan GA, Lynch JW. Relation between income inequality and mortality in Canada and in the United States: cross sectional assessment using census data and vital statistics. BMJ. 2000;320(7239):898-902. | 4 |
| Rowley KG, O'Dea K, Anderson I, McDermott R, Saraswati K, Tilmouth R, et al. Lower than expected morbidity and mortality for an Australian Aboriginal population: 10-year follow-up in a decentralised community. Medical J Australia. 2008;188(5):283-7. | 2 |
| Ruiz JM, Steffen P, Smith TB. Hispanic mortality paradox: a systematic review and meta-analysis of the longitudinal literature. Am J Public Health. 2013;103(3):e52-60. | 2 |
| Ruiz-Ramos M, Escolar Pujolar A, Sanchez Perea J, Garrucho Rivero G. Trends in social inequalities in mortality in the city of Seville [Spain] [1994-2002]. [Spanish]. Gaceta Sanitaria. 2006;20(4):303-10. | 6 |
| Sabanayagam C, Shankar A. Income is a stronger predictor of mortality than education in a national sample of US adults. J Health, Population and Nutrition. 2012;30(1):82-6. | 2 |
| Safaei J. Income and health inequality across Canadian provinces. Health & Place. 2007;13(3):629-38. | 2 |
| Safaei J. Post-communist Health Transitions in Central and Eastern Europe. Econ Res Int. 2012. | 4 |
| Said A, Guan H. A decline in adult mortality, ages 45--64, in Wisconsin over the last 20 years: is it enough? WMJ. 2003;102(8):47-51. | 2 |
| Sainsbury P, Harris E. Understanding the causes of health inequalities: incorporating personal, local, national, and global perspectives. NSW Public Health Bulletin. 2002;13(6):121-3. | 4 |
| Sanmartin C, Ross NA, Tremblay S, Wolfson M, Dunn JR, Lynch J. Labour market income inequality and mortality in North American metropolitan areas. J Epidemiol Community Health. 2003;57(10):792-7. | 4 |
| Santos JV, Freitas A. Self-perceived health inequalities by income distribution in European Union. Value in Health. 2018;21:S175-S. | 4 |
| Scheiring G, Irdam D, King LP. Cross-country evidence on the social determinants of the post-socialist mortality crisis in Europe: a review and performance-based hierarchy of variables. Sociology of Health & Illness. 2019;41(4):673-91. | 4 |
| Schneider MPA, Yasar Y. Is inequality deadly and for whom? A Bayesian Model Averaging analysis. Social Science Journal. 2016;53(3):357-70. | 4 |
| Schutte S, Chastang JF, Parent-Thirion A, Vermeylen G, Niedhammer I. Association between socio-demographic, psychosocial, material and occupational factors and self-reported health among workers in Europe. J Public Health. 2014;36(2):194-204. | 2 |
| Senn S. Mortality and distribution of income. Societies with narrower income distributions are healthier. BMJ. 1998;316(7144):1611-2. | 4 |
| Shah A, Bhat R, Mackenzie S, Koen C. A cross-national study of the relationship between elderly suicide rates and life expectancy and markers of socioeconomic status and health care. Int Psychogeriatrics. 2008;20(2):347-60. | 5 |
| Shams K. Income and Health Satisfaction: Evidence from Rural Pakistan. J Happiness Studies. 2015;16(6):1455-74. | 3 |
| Shartova N, Tikunov V, Chereshnya O. Health disparities in Russia at the regional and global scales. Int J Equity Health. 2021;20(1):163. | 2 |
| Shi J, Tarkiainen L, Martikainen P, van Raalte A. The impact of income definitions on mortality inequalities. Soc Sci Med - Population Health. 2021;15:100915. | 2 |
| Shi L, Macinko J, Starfield B, Politzer R, Xu J. Primary care, race, and mortality in US states. Soc Sci Med. 2005;61(1):65-75. | 4 |
| Shi L, Macinko J, Starfield B, Wulu J, Regan J, Politzer R. The relationship between primary care, income inequality, and mortality in US States, 1980--1995. J Am Board of Family Practice. 2003;16(5):412-22. | 4 |
| Shi L, Starfield B. The effect of primary care physician supply and income inequality on mortality among blacks and whites in US metropolitan areas. Am J Public Health. 2001;91(8):1246-50. | 4 |
| Shmueli A. Population health and income inequality: new evidence from Israeli time-series analysis. Int J Epidemiol. 2004;33(2):311-7. | 4 |
| Siddiqi A, Hertzman C. Economic growth, income equality, and population health among the Asian Tigers. Int J Social Determinants of Health and Health Serv. 2001;31(2):323-33. | 4 |
| Siegel M, Mosler K. Semiparametric modelling of age-specific variations in income related health inequalities. Health Econ. 2014;23(7):870-8. | 4 |
| Simons AMW, Groffen DAI, Bosma H. Socio-economic inequalities in all-cause mortality in Europe: an exploration of the role of heightened social mobility. Eur J Public Health. 2013;23(6):1010-2. | 4 |
| Singh GK, Siahpush M. Increasing inequalities in all-cause and cardiovascular mortality among US adults aged 25--64 years by area socioeconomic status, 1969--1998. Int J Epidemiol. 2002;31(3):600-13. | 2 |
| Spence ND. Does Social Context Matter? Income Inequality, Racialized Identity, and Health Among Canada’s Aboriginal Peoples Using a Multilevel Approach. J Racial and Ethnic Health Disparities. 2016;3(1):21-34. | 2 |
| Stacy C, Meixell B, Srini T. Inequality Versus Inclusion in US Cities. Social Indicators Res. 2019;145(1):117-56. | 4 |
| Stafford M, Cummins S, Macintyre S, Ellaway A, Marmot M. Gender differences in the associations between health and neighbourhood environment. Soc Sci Med. 2005;60(8):1681-92. | 2 |
| Stanistreet D, Scott-Samuel A, Bellis MA. Income inequality and mortality in England. J Public Health. 1999;21(2):205-7. | 5 |
| Starfield B, Shi LY. Policy relevant determinants of health: an international perspective. Health Policy. 2002;60(3):201-18. | 2 |
| Subramanian SV, Kawachi I. Response: In defence of the income inequality hypothesis. Int J Epidemiol. 2003;32(6):1037-40. | 4 |
| Subramanian SV, Kawachi I. Commentary: Plugging leaks and repelling boarders - where to next for the SS Income Inequality? Response: In defence of the income inequality hypothesis. Int J Epidemiol. 2003;32(6):1037-40. | 4 |
| Subramanian SV, Kawachi I. Income inequality and health: what have we learned so far? Epidemiologic Reviews. 2004;26:78-91. | 4 |
| Subramanian SV, Kawachi I. Commentary: Chasing the elusive null - the story of income inequality and health. Int J Epidemiol. 2007;36(3):596-9. | 4 |
| Subramanyam M, Kawachi I, Berkman L, Subramanian SV. Relative deprivation in income and self-rated health in the United States. Soc Sci Med. 2009;69(3):327-34. | 2 |
| Subramanian SV, Kim D, Kawachi I. Covariation in the socioeconomic determinants of self rated health and happiness: a multivariate multilevel analysis of individuals and communities in the USA. J Epidemiol Community Health. 2005;59(8):664-9. | 2 |
| Subramanian SV, Kubzansky L, Berkman L, Fay M, Kawachi I. Neighborhood effects on the self-rated health of elders: uncovering the relative importance of structural and service-related neighborhood environments. The J Gerontology. 2006;61(3):S153-60. | 2 |
| Sutton GC. Mortality and distribution of income. Widening income inequalities cause poorer health. BMJ. 1998;316(7144):1612. | 4 |
| Szczepaniak M, Geise A. Examining the relationships between income inequalities and different dimensions of well-being in selected Central Eastern European (CEE) countries. PLoS One. 2021;16(4):e0250469. | 4 |
| Szeles MR. Comparative Examination of Self-Perceived Health and Other Measures of the Quality of Life Across the EU-27. Social Indicators Res. 2018;137(1):391-411. | 4 |
| Szwarcwald CL, Bastos FI, Esteves MA, de Andrade CL, Paez MS, Medici EV, et al. [Income inequality and health: the case of Rio de Janeiro]. Cadernos de saude publica. 1999;15(1):15-28. | 6 |
| Torre R, Myrskyla M. Income Inequality and Population Health: An Analysis of Panel Data for 21 Developed Countries, 1975--2006. Population Studies. 2014;68(1):1-13. | 4 |
| Tridico P. Growth, Inequality and Poverty in Emerging and Transition Economies. Transition Studies Review. 2010;16(4):979-1001. | 4 |
| Undurraga EA, Behrman JR, Leonard WR, Godoy RA. The effects of community income inequality on health: Evidence from a randomized control trial in the Bolivian Amazon. Soc Sci Med. 2016;149:66-75. | 2 |
| Vafaei A, Rosenberg MW, Pickett W. Relationships between income inequality and health: a study on rural and urban regions of Canada. Rural and Remote Health. 2010;10(2). | 4 |
| van Doorslaer E, Wagstaff A, Bleichrodt H, Calonge S, Gerdtham UG, Gerfin M, et al. Income-related inequalities in health: some international comparisons. J Health Econ. 1997;16(1):93-112. | 4 |
| Van Ourti T, van Doorslaer E, Koolman X. The effect of income growth and inequality on health inequality: Theory and empirical evidence from the European Panel. J Health Econ. 2009;28(3):525-39. | 4 |
| Veenstra G. Social capital and health (plus wealth, income inequality and regional health governance). Soc Sci Med. 2002;54(6):849-68. | 4 |
| Veenstra G. Income inequality and health. Coastal communities in British Columbia, Canada. Canadian J Public Health. 2002;93(5):374-9. | 5 |
| Veugelers PJ, Yip AM, Kephart G. Proximate and contextual socioeconomic determinants of mortality: multilevel approaches in a setting with universal health care coverage. Am J Epidemiol. 2001;154(8):725-32. | 2 |
| Vincens N, Emmelin M, Stafstrom M. Social capital, income inequality and the social gradient in self-rated health in Latin America: A fixed effects analysis. Soc Sci Med. 2018;196:115-22. | 2 |
| Wagstaff A, van Doorslaer E. Income inequality and health: what does the literature tell us? Annual Review of Public Health. 2000;21:543-67. | 4 |
| Ward JL, Viner RM. The impact of changes in national wealth and income inequality on early life course mortality in low and middle-income countries 1990--2012. Archives of Disease in Childhood. 2017;102 (Supplement 1):A127. | 3 |
| Weaver RA, Rivello R. The distribution of mortality in the United States: The effects of income (inequality), social capital, and race. Omega: J Death and Dying. 2007;54(1):19-39. | 4 |
| Wight RG, Cummings JR, Miller-Martinez D, Karlamangla AS, Seeman TE, Aneshensel CS. A multilevel analysis of urban neighborhood socioeconomic disadvantage and health in late life. Soc Sci Med. 2008;66(4):862-72. | 2 |
| Wildman J. The impact of income inequality on individual and societal health: Absolute income, relative income and statiscal artefacts. Health Econ. 2001;10(4):357-61. | 4 |
| Wildman J. Modelling health, income and income inequality: the impact of income inequality on health and health inequality. J Health Econ. 2003;22(4):521-38. | 4 |
| Wildman J, Gravelle H, Sutton M. Health and income inequality: attempting to avoid the aggregation problem. Applied Econ. 2003;35(9):999-1004. | 3 |
| Wilkinson R. Changing Inequalities in Rich Countries: Analytical and Comparative Perspectives. Sociologicky Casopis-Czech Sociological Review. 2015;51(3):550-6. | 4 |
| Wilkinson R, Bezruchka S. Income inequality and population health. Better measures of social differentiation and hierarchy are needed. BMJ. 2002;324(7343):978. | 4 |
| Wilkinson RG. Income distribution and life expectancy. BMJ. 1992;304(6820):165-8. | 4 |
| Wilkinson RG. Inequalities and health. Lancet. 1994;343(8896):538. | 4 |
| Wilkinson RG. Commentary: income inequality summarises the health burden of individual relative deprivation. BMJ. 1997;314(7096):1727-8. | 4 |
| Wilkinson RG. Comment: income, inequality, and social cohesion. Am J Public Health. 1997;87(9):1504-6. | 4 |
| Wilkinson RG. Mortality and distribution of income. Low relative income affects mortality. BMJ. 1998;316(7144):1611-2. | 4 |
| Wilkinson RG. Income inequality and population health. Soc Sci Med. 1998;47(3):411-2. | 4 |
| Wilkinson RG. Commentary: The changing relation between mortality and income. Int J Epidemiol. 2007;36(3):492-4; discussion 502. | 4 |
| Wilkinson RG, Kawachi I, Kennedy BP. Mortality, the social environment, crime and violence. Sociology of Health & Illness. 1998;20(5):578-97. | 4 |
| Wilkinson RG, Pickett KE. Income inequality and population health: a review and explanation of the evidence. Soc Sci Med. 2006;62(7):1768-84. | 4 |
| Wilkinson RG, Pickett KE. Income inequality and socioeconomic gradients in mortality. Am J Public Health. 2008;98(4):699-704. | 4 |
| Wolfson MC, Kaplan G, Lynch J, Ross N, Backlund E. Relation between income inequality and mortality: empirical demonstration. Western J Medicine. 2000;172(1):22-4. | 4 |
| Wu W. Replication Data for: The impact of income inequality on mortality. A replicaiton study of Leigh and Jencks (J Health Economics, 2007). Harvard Dataverse. 2022;2. | 5 |
| Yang M, Eldridge S, Merlo J. Multilevel survival analysis of health inequalities in life expectancy. Int Journal for Equity in Health. 2009;8 (no pagination). | 2 |
| Yang T-C, Jensen L. Exploring the Inequality-Mortality Relationship in the US with Bayesian Spatial Modeling. Population Res and Policy Review. 2015;34(3):437-60. | 4 |
| Yang TC, Chen VYJ, Shoff C, Matthews SA. Using quantile regression to examine the effects of inequality across the mortality distribution in the U.S. counties. Soc Sci Med. 2012;74(12):1900-10. | 4 |
| Yang TC, Matthews SA, Park K. Looking through a different lens: Examining the inequality-mortality association in US counties using spatial panel models. Applied Geography. 2017;86:139-51. | 4 |
| Yngwe MA, Diderichsen F, Burstrom B, Whitehead M, Holland P. The role of income differences in explaining social inequalities in self rated health in Sweden and Britain. J Epidemiol Community Health. 2001;55(8):556-61. | 2 |
| Zhao Z. Income Inequality, Unequal Health Care Access, and Mortality in China. Population and Development Review. 2006;32(3):461-83. | 4 |

^a^Reason for exclusion: 1 = Used individual-level data (*n =* 15); 2 = Wrong exposure – exposure not measure of distribution of income across an area (*n =* 89); 3 = Wrong outcome – outcome not self-rated health or all-cause mortality (*n =* 26); 4 = Wrong study design – not primary study or study using multilevel data (*n =* 208); 5 = Wrong covariate adjustments – did not adjust for individual-level socioeconomic position (*n =* 22); 6 = Wrong language – not in English or could not find translation (*n =* 9)

Appendix 14 List of Included Studies

| Primary meta-analysis: self-rated health |
| --- |
| ^129^Adeline A, Delattre E. Some microeconometric evidence on the relationship between health and income. *Health Econ Rev*. 2017;7(1):27. |
| ^130^Baum S, Kendall E, Parekh S. Self-assessed health status and neighborhood context. *J Prev Interv Community*. 2016;44(4):283-295. |
| ^131^Bjornstrom EES. The neighborhood context of relative position, trust, and self-rated health. *Soc Sci Med*. 2011;73(1):42-49. |
| ^132^Bobak M, Murphy M, Rose R, Marmot M. Societal characteristics and health in the former communist countries of Central and Eastern Europe and the former Soviet Union: a multilevel analysis. *J Epidemiol Community Health*. 2007;61(11):990. |
| ^133^Bobak M, Pikhart H, Rose R, Hertzman C, Marmot M. Socioeconomic factors, material inequalities, and perceived control in self-rated health: cross-sectional data from seven post-communist countries. *Soc Sci Med*. 2000;51(9):1343-1350. |
| ^134^Cai JL, Laporte A, Zhang L, et al. Impacts of absolute and relative income on self-rated health in urban and rural China. *Int J Health Serv*. 2022;52(1):129-140. |
| ^135^Chen Z, Gotway Crawford CA. The role of geographic scale in testing the income inequality hypothesis as an explanation of health disparities. *Soc Sci Med*. 2012;75(6):1022-1031. |
| ^136^Chiavegatto ADP, Lebrao ML, Kawachi I. Income inequality and elderly self-rated health in Sao Paulo, Brazil. *Ann Epidemiol*. 2012;22(12):863-867. |
| ^137^Choi H, Burgard S, Elo IT, Heisler M. Are older adults living in more equal counties healthier than older adults living in more unequal counties? A propensity score matching approach. *Soc Sci Med*. 2015;141:82-90. |
| ^138^Craig N. Exploring the generalisability of the association between income inequality and self-assessed health. *Soc Sci Med*. 2005;60(11):2477-2488. |
| ^139^Evans MDR, Kelley J, Kelley CGE, Kelley SMC. Income inequality in the great recession did not harm subjective health in Europe, 2003--2012. *Appl Res Qual Life*. 2020;15(5):1451-1473. |
| ^140^Feng Z, Wang WW, Jones K, Li Y. An exploratory multilevel analysis of income, income inequality and self-rated health of the elderly in China. *Soc Sci Med*. 2012;75(12):2481-2492. |
| ^141^Fiscella K, Franks P. Individual income, income inequality, health, and mortality: what are the relationships? *Health Serv Res*. 2000;35(1):307-318. |
| ^142^Góngora-Salazar P, Casabianca MS, Rodríguez-Lesmes P. Income inequality and self-rated health status in Colombia. *Int J Equity Health*. 2022;21(1):69. |
| ^143^Gravelle H, Sutton M. Income, relative income, and self-reported health in Britain 1979--2000. *J Health Econ*. 2009;18(2):125-145. |
| ^144^Haithcoat TL, Avery EE, Bowers KA, Hammer RD, Shyu CR. Income inequality and health: expanding our understanding of state-level effects by using a geospatial big data approach. *Soc Sci Comput Rev*. 2021;39(4):543-561. |
| ^145^Hildebrand V, Van Kerm P. Income inequality and self-rated health status: evidence from the European Community Household Panel. *Demography*. 2009;46(4):805-825. |
| ^146^Hou F, Myles J. Neighbourhood inequality, neighbourhood affluence and population health. *Soc Sci Med*. 2005;60(7):1557-1569. |
| ^147^Ichida Y, Kondo K, Hirai H, Hanibuchi T, Yoshikawa G, Murata C. Social capital, income inequality and self-rated health in Chita peninsula, Japan: a multilevel analysis of older people in 25 communities. *Soc Sci Med*. 2009;69(4):489-499. |
| ^148^Jen MH, Jones K, Johnston R. Global variations in health: evaluating Wilkinson’s income inequality hypothesis using the World Values Survey. *Soc Sci Med*. 2009;68(4):643-653. |
| ^149^Kahn RS, Wise PH, Kennedy BP, Kawachi I. State income inequality, household income, and maternal mental and physical health: cross sectional national survey. *BMJ*. 2000;321(7272):1311-1315. |
| ^150^Karlsdotter K, Martin JJM, Gonzalez M. Multilevel analysis of income, income inequalities and health in Spain. *Soc Sci Med*. 2012;74(7):1099-1106. |
| ^151^Kennedy BP, Kawachi I, Glass R, Prothrow-Stith D. Income distribution, socioeconomic status, and self rated health in the United States: multilevel analysis. *BMJ*. 1998;317(7163):917-921. |
| ^152^LeClere FB, Soobader MJ. The effect of income inequality on the health of selected US demographic groups. *Am J Public Health*. 2000;90(12):1892-1897. |
| ^153^Massa KHC, Pabayo R, Chiavegatto Filho ADP. Income inequality and self-reported health in a representative sample of 27 017 residents of state capitals of Brazil. *J Public Health*. 2018;40(4):e440-e446. |
| ^154^Oshio T, Kobayashi M. Income inequality, area-level poverty, perceived aversion to inequality, and self-rated health in Japan. *Soc Sci Med*. 2009;69(3):317-326. |
| ^155^Qi YQ. The impact of income inequality on self-rated general health: evidence from a cross-national study. *Res Soc Stratif Mobil*. 2012;30(4):451-471. |
| ^156^Rostila M, Kolegard ML, Fritzell J. Income inequality and self-rated health in Stockholm, Sweden: a test of the ‘income inequality hypothesis’ on two levels of aggregation. *Soc Sci Med*. 2012;74(7):1091-1098. |
| ^157^Shibuya K, Hashimoto H, Yano E. Individual income, income distribution, and self rated health in Japan: cross sectional analysis of nationally representative sample. *BMJ*. 2002;324(7328):16-19. |
| ^158^Sommet N, Elliot AJ. The effects of U.S. county and state income inequality on self-reported happiness and health are equivalent to zero. *Quality of Life Res*. 2022;31(7):1999-2009. |
| ^159^Subramanian SV, Blakely T, Kawachi I. Income inequality as a public health concern: where do we stand? Commentary on “Is exposure to income inequality a public health concern?” *Health Serv Res*. 2003;38(1 Pt 1):153-167. |
| ^160^Subramanian SV, Delgado I, Jadue L, Vega J, Kawachi I. Income inequality and health: multilevel analysis of Chilean communities. *J Epidemiol Community Health*. 2003;57(11):844-848. |
| ^161^Vauclair CM, Marques S, Lima ML, et al. Perceived age discrimination as a mediator of the association between income inequality and older people’s self-rated health in the European region. *J Gerontol B Psychol Sci Soc Sci*. 2015;70(6):901-912. |
| ^162^Weich S, Lewis G, Jenkins SP. Income inequality and self rated health in Britain. *J Epidemiol Community Health*. 2002;56(6):436-441. |
| ^163^Wen M, Browning CR, Cagney KA. Poverty, affluence, and income inequality: neighborhood economic structure and its implications for health. *Soc Sci Med*. 2003;57(5):843-860. |
| ^164^Wong IO, Cowling BJ, Lo SV, Leung GM. A multilevel analysis of the effects of neighbourhood income inequality on individual self-rated health in Hong Kong. *Soc Sci Med*. 2009;68(1):124-132. |
| ^165^Xi GL, McDowell I, Nair R, Spasoff R. Income inequality and health in Ontario - a multilevel analysis. *Can J Public Health*. 2005;96(3):206-211. |
| ^166^Zheng H. Rising U.S. income inequality, gender and individual self-rated health, 1972--2004. *Soc Sci Med*. 2009;69(9):1333-1342. |
| Primary meta-analysis: all-cause mortality |
| ^167^Auger N, Hamel D, Martinez J, Ross NA. Mitigating effect of immigration on the relation between income inequality and mortality: a prospective study of 2 million Canadians. *J Epidemiol Community Health*. 2012;66(6):e5. |
| ^168^Backlund E, Rowe G, Lynch J, Wolfson MC, Kaplan GA, Sorlie PD. Income inequality and mortality: a multilevel prospective study of 521 248 individuals in 50 US states. *Int J Epidemiol*. 2007;36(3):590-596. |
| ^169^Blakely T, Atkinson J, O’Dea D. No association of income inequality with adult mortality within New Zealand: a multi-level study of 1.4 million 25–64 year olds. *J Epidemiol Community Health*. 2003;57(4):279. |
| ^170^Gerdtham U-G, Johannesson M. Absolute income, relative income, income inequality, and mortality. *J Hum Resour*. 2004;39(1):228-247. |
| ^171^Henriksson G, Allebeck P, Weitoft GR, Thelle D. Are manual workers at higher risk of death than non-manual employees when living in Swedish municipalities with higher income inequality? *Eur J Public Health*. 2007;17(2):139-144. |
| ^172^Kimmel PL, Fwu CW, Eggers PW. Segregation, income disparities, and survival in hemodialysis patients. *J the Am Society of Nephrology*. 2013;24(2):293-301. |
| ^173^Kravdal O. Does income inequality really influence individual mortality? Results from a ‘fixed-effects analysis’ where constant unobserved municipality characteristics are controlled. *Demogr Res*. 2008;18:205-232. |
| ^174^Modrek S, Dow WH, Rosero-Bixby L. Long-term association of economic inequality and mortality in adult Costa Ricans. *Soc Sci Med*. 2012;74(2):158-166. |
| ^175^Ng N, Lundevaller E, Malmberg G, Edvinsson S. Income inequality and old-age mortality in Sweden: do regional development and lagged effect matter? *Health Place*. 2020;64:102384. |
| ^176^Osler M, Prescott E, Gronbaek M, Christensen U, Due P, Engholm G. Income inequality, individual income, and mortality in Danish adults: analysis of pooled data from two cohort studies. *BMJ*. 2002;324(7328):13-16. |
| ^177^Pabayo R, Chiavegatto Filho AD, Lebrao ML, Kawachi I. Income inequality and mortality: results from a longitudinal study of older residents of Sao Paulo, Brazil. *Am J Public Health (N Y)*. 2013;103(9):e43-e49. |
| ^178^Zhao L, Hessel P, Simon Thomas J, Beckfield J. Inequality in place: effects of exposure to neighborhood-level economic inequality on mortality. *Demography*. 2021;58(6):2041-2063. |
| ^179^Zheng H. Do people die from income inequality of a decade ago? *Soc Sci Med*. 2012;75(1):36-45. |
| Subgroup/sensitivity analyses: self-rated health |
| ^180^Caicedo-Velasquez B, Restrepo-Mendez MC. The role of individual, household, and area of residence factors on self-rated health in Colombian adults: a multilevel study. *Biomedica*. 2020;40(2):296-308. |
| ^181^Latif E. Income inequality and health: panel data evidence from Canada. *BE J Econ Anal Policy*. 2015;15(2):927-959. |
| ^182^Massa KHC, Chiavegatto ADP. Income inequality and self-reported health among older adults in Brazil. *J Appl Gerontol*. 2021;40(2):152-161. |
| ^183^Pei X, Rodriguez E. Provincial income inequality and self-reported health status in China during 1991--7. *J Epidemiol Community Health*. 2006;60(12):1065-1069. |
| ^184^Subramanian SV, Kawachi I. The association between state income inequality and worse health is not confounded by race. *Int J Epidemiol*. 2003;32(6):1022-1028. |
| ^185^Subramanian SV, Kawachi I, Kennedy BP. Does the state you live in make a difference? Multilevel analysis of self-rated health in the US. *Soc Sci Med*. 2001;53(1):9-19. |
| ^186^Zimmerman FJ, Bell JF. Income inequality and physical and mental health: testing associations consistent with proposed causal pathways. *J Epidemiol Community Health*. 2006;60(6):513-521. |
| Subgroup/sensitivity analyses: all-cause mortality |
| ^187^Fiscella K, Franks P. Poverty or income inequality as predictor of mortality: longitudinal cohort study. *BMJ*. 1997;314(7096):1724-1727. |
| ^188^Dahl E, Elstad JI, Hofoss D, Martin-Mollard M. For whom is income inequality most harmful? A multi-level analysis of income inequality and mortality in Norway. *Soc Sci Med*. 2006;63(10):2562-2574. |
| ^189^Daly MC, Duncan GJ, Kaplan GA, Lynch JW. Macro-to-micro links in the relation between income inequality and mortality. *Milbank Q*. 1998;76(3):315-339. |
| ^190^Elstad JI. Does the socioeconomic context explain both mortality and income inequality? Prospective register-based study of Norwegian regions. *Int J Equity Health*. 2011;10:7. |
| ^191^Grönqvist H, Johansson P, Niknami S. Income inequality and health: lessons from a refugee residential assignment program. *J Health Econ*. 2012;31(4):617-629. |
| ^192^Henriksson G, Allebeck P, Weitoft GR, Thelle D. Income distribution and mortality: implications from a comparison of individual-level analysis and multilevel analysis with Swedish data. *Scand J Public Health*. 2006;34(3):287-294. |
| ^193^Kahn HS, Patel AV, Jacobs EJ, Calle EE, Kennedy BP, Kawachi I. Pathways between area-level income inequality and increased mortality in U.S. men. *Ann N Y Acad Sci*. 1999;896:332-334. |
| ^194^Lau EW, Schooling MC, Tin KY, Leung GM. Income inequality and cause-specific mortality during economic development. *Ann Epidemiol*. 2012;22(4):285-294. |
| Duplicates (not included in analyses): self-rated health |
| ^107^Aida J, Kondo K, Kondo N, Watt RG, Sheiham A, Tsakos G. Income inequality, social capital and self-rated health and dental status in older Japanese. *Soc Sci Med*. 2011;73(10):1561-1568. |
| ^108^Blakely TA, Kennedy BP, Glass R, Kawachi I. What is the lag time between income inequality and health status? *J Epidemiol Community Health*. 2000;54(4):318-319. |
| ^109^Blakely TA, Kennedy BP, Kawachi I. Socioeconomic inequality in voting participation and self-rated health. *Am J Public Health*. 2001;91(1):99-104. |
| ^110^Blakely TA, Lochner K, Kawachi I. Metropolitan area income inequality and self-rated health - a multi-level study. *Soc Sci Med*. 2002;54(1):65-77. |
| ^111^Fang H, Rizzo JA. Does inequality in China affect health differently in high- versus low-income households? *App Econ*. 2012;44(9):1081-1090. |
| ^112^Hou F, Chen J. Neighbourhood low income, income inequality and health in Toronto. *Health Rep*. 2003;14(2):21-34. |
| ^113^Li H, Zhu Y. Income, income inequality, and health: Evidence from China. *J Comparative Econ*. 2006;34(4):668-693. |
| ^114^Lopez R. Income inequality and self-rated health in US metropolitan areas: a multi-level analysis. *Soc Sci Med*. 2005;61(10):2267-2409. |
| ^115^Lorgelly PK, Lindley J. What is the relationship between income inequality and health? Evidence from the BHPS. *Health Econ*. 2008;17(2):249-265. |
| ^116^Mansyur C, Amick BC, Harrist RB, Franzini L. Social capital, income inequality, and self-rated health in 45 countries. *Soc Sci Med*. 2008;66(1):43-56. |
| ^117^McLeod CB, Lavis JN, Mustard CA, Stoddart GL. Income inequality, household income, and health status in Canada: a prospective cohort study. *Am J Public Health*. 2003;93(8):1287-1293. |
| ^118^Mellor JM, Milyo J. Income inequality and health status in the United States - evidence from the current population survey. *J Human Resources*. 2002;37(3):510-539. |
| ^119^Mellor JM, Milyo J. Is exposure to income inequality a public health concern? Lagged effects of income inequality on individual and population health. *Health Serv Res*. 2003;38(1):137-151 |
| ^120^Oshio T, Kobayashi M. Income inequality, perceived happiness, and self-rated health: evidence from nationwide surveys in Japan. *Soc Sci Med*. 2010;70(9):1358-1366. |
| ^121^Pasqualini M, Lanari D, Minelli L, Pieroni L, Salmasi L. Health and income inequalities in Europe: what is the role of circumstances? *Econ Hum Biol*. 2017;26:164-173. |
| ^122^Rozer J, Kraaykamp G, Huijts T. National income inequality and self-rated health: the differing impact of individual social trust across 89 countries. *Eur Soc*. 2016;18(3):245-263. |
| ^123^Rozer JJ, Volker B. Does income inequality have lasting effects on health and trust? *Soc Sci Med*. 2016;149:37-45. |
| ^124^Shi L, Starfield B. Primary care, income inequality, and self-rated health in the United States: a mixed-level analysis. *Int J Health Serv*. 2000;30(3):541-555. |
| ^125^Soobader MJ, LeClere FB. Aggregation and the measurement of income inequality: effects on morbidity. *Soc Sci Med*. 1999;48(6):733-744. |
| ^126^Subramanian SV, Kawachi I. Whose health is affected by income inequality? A multilevel interaction analysis of contemporaneous and lagged effects of state income inequality on individual self-rated health in the United States. *Health Place*. 2006;12(2):141-156. |
| ^127^Ting S, Zang W, Chen C, Chen D. Income distribution and health: what do we know from Chinese data? *PLoS One*. 2022;17(1):e0263008. |
| ^128^Zagorski K, Evans MDR, Kelley J, Piotrowska K. Does national income inequality affect individuals’ quality of life in europe? inequality, happiness, finances, and health. *Soc Indic Res*. 2014;117(3):1089-1110. |
| Duplicates (not included in analyses): all-cause mortality |
| ^101^Edvinsson S, Lundevaller EH, Malmberg G. Do unequal societies cause death among the elderly? A study of the health effects of inequality in Swedish municipalities in 2006. *Glob Health Action*. 2013;6:1-9. |
| ^102^Eibner C, Evans WN. Relative deprivation, poor health habits, and mortality. *J Human Resources*. 2005;40(3):591-620. |
| ^103^Kim D. The associations between US state and local social spending, income inequality, and individual all-cause and cause-specific mortality: the National Longitudinal Mortality Study. *Prev Med*. 2016;84:62-68. |
| ^104^Kinge JM, Vallejo-Torres L, Morris S. Income related inequalities in avoidable mortality in Norway: a population-based study using data from 1994--2011. *Health Policy*. 2015;119(7):889-898. |
| ^105^Lochner K, Pamuk E, Makuc D, Kennedy BP, Kawachi I. State-level income inequality and individual mortality risk: a prospective, multilevel study. *Am J Public Health*. 2001;91(3):385-391. |
| ^106^Waitzman NJ, Smith KR, Stroup A. The direct and indirect effects of metropolitan area inequality on mortality. a hierarchical analysis. *Ann N Y Acad Sci*. 1999;896:347-349. |

Appendix 15 Characteristics of Included Studies Reporting the Association Between Income Inequality and Self-Rated Health (SRH)

| **Author, year** | **Study information (study year/name, geographical scale, sample size and age)** | **Reported outcomes (follow-up time and no. of events)** | **Covariate adjustments for statistical model used in primary meta-analysis** | |
| --- | --- | --- | --- | --- |
| **Moderate risk of bias** | | | |  |
| Craig, 2005[^138^](#bib138) | 1999-2000 Scottish household survey (SHS); Scotland, 32 local authorities (Gini = 0.287). Sample: *n =* 28,340, age: 16--64. | Lower 2 of 3 (10,486 cases, 37.0% of total) | Individual-level: age, gender, income, economic status, education  Area-level: mean local authority income | |
| Góngora-Salazar et al., 2022[^142^](#bib142) | 2011-2019 Departamento Administrativo Nacional de Estadística (DANE); Colombia, 9 regions (Gini = 0.51). Sample: *n* = 567,678, age: 24--75. | Lower 2 of 4 (130,566 cases, 23.0% of total) | Individual-level: age, gender, household income, household size, equivalised income, ethnicity, marital status, health insurance status, educational level, employment status, urban dwelling, household tenure  Area-level: mean regional income, area-level socioeconomic development | |
| Karlsdotter et al., 2012[^150^](#bib150) | 2007 Life Conditions Survey (LCS); Spain, 17 regions (Gini = 0.272). Sample: *n* = 28,023, age: 17–88. | Lower 2 of 5 (9,584 cases, 34.2% of total) | Individual-level: age, gender, personal income, education level, unemployment, marital status, nationality  Area-level: NR | |
| Shibuya et al., 2002[^157^](#bib157) | 1995 Comprehensive Survey of Living Conditions of People on Health and Welfare (CSLCPHW); Japan, 46 prefectures (Gini = 0.287). Sample: *n* = 80,900, age: >15. | Lower 2 of 5 (8,415 cases, 10.4% of total) | Individual-level: age, gender, household income, marital status, medical check-up  Area-level: dummy variables for 12 geopolitical blocks in Japan | |
| Subramanian et al., 2003[^159^](#bib159) | 1995 – 1997 Current Population Survey (CPS); United States, 50 states (Gini = 0.429). Sample: *n* = 90,006, age: >45. | Lower 2 of 5 (cases NR) | Individual-level: age groups, gender, equivalized household income, race  Area-level: state-level median household income, divisional dummies for each census division in the United States | |
| Subramanian et al., 2003[^160^](#bib160) | 2000 National Socioeconomic Characterization Survey (CASEN); Chile, 285 communities (Gini = 0.528). Sample: *n* = 98,344, age: 15--99. | Lower 2 of 5 (8,513 cases, 8.7% of total) | Individual-level: age, gender, equivalised monthly household income, education, employment status, marital status, ethnicity, type of insurance, urban-rural residence  Area-level: community median income | |
| Weich et al., 2002[^162^](#bib162) | 1991 British Household Panel Survey (BHPS); UK, 18 regions (Gini = 0.29). Sample: *n* = 8,366, age: 16–75. | Lower 2 of 5 (2,431 cases, 29.1% of total) | Individual-level: age, gender, social class by head of household, education, employment, marital status, ethnicity, housing tenure  Area-level: NR | |
| **Serious risk of bias** | | | |  |
| Adeline and Delattre, 2017[^129^](#bib129) | 2013 Survey of Health, Ageing and Retirement in Europe (SHARE); Europe, 15 countries (Gini = 0.33). Sample: *n* = 63,626, age: >50. | Highest of 5^d^ (cases NR) | Individual-level: age, gender, individual income, income squared, number of years of education, job situation, marital status  Area-level: GDP, country dummy variables | |
| Evans and Kelley, 2020[^139^](#bib139) | 2003-2012 European Quality of Life Surveys (EQLS); Europe, 24 countries (Gini = 0.317). Sample: *n* = 64,912, age: >18. | Highest of 5^d^ (cases NR) | Individual-level: age, gender, income, education, marital status, attended worship services  Area-level: GDP | |
| Feng et al., 2012[^140^](#bib140) | 2008 Chinese Longitudinal Healthy Longevity Survey (CLHLS); China, 23 provinces (Gini = 0.5). Sample: *n* = 14,744, age: 60–116. | Lower 2 of 5 (2,395 cases, 16.2% of total) | Individual-level: age, gender, family equivalised income, education, schooling/non-schooling, with/without old-age insurance, urban/rural residence  Area-level: NR | |
| Gravelle and Sutton, 2009[^143^](#bib143) | 1979-2001 British General Household Survey (GHS); UK, 11 regions (Gini = 0.323). Sample: *n* = 231,208, age: 17--29. | Highest of 3^d^ (cases NR) | Individual-level: age, gender, gross household income, social class, educational attainment, housing tenure, health status  Area-level: regional mean income | |
| Haithcoat et al., 2021[^144^](#bib144) | 2014-2016 Behavioural Risk Factor Surveillance System (BRFSS); United States, 48 states (Gini = 0.46). Sample: *n* = 95,461, age: 18--80. | Lower 2 of 5 (17,183 cases, 18.0% of total) | Individual-level: age, gender, race/ethnicity, educational attainment, relationship status, income, insurance, current smoker, alcohol consumption, exercise in past 30 days  Area-level: median household income, % on SNAP, % insured | |
| Hildebrand and Van, 2009[^145^](#bib145) | 1995-2001 European Community Household Panel survey (ECHP); Europe, 52 regions (Gini = 0.3). Sample: *n* = 506,598, age: 25--74. | Lower 2 of 5 (42,336 cases, 8.4% of total) | Individual-level: age, gender, household income, education, marital status  Area-level: country of residence | |
| Hou and Myles, 2005[^146^](#bib146) | 1996-1997 Canadian National Population Health Survey (CNPHS); Canada, Neighbourhoods (Gini = 0.287) (number of areas NR). Sample: *n* = 34,592, age: >15. | Highest of 5^d^ (cases NR) | Individual-level: age, gender, family income, education, racial minority status, immigrant status  Area-level: neighbourhood median income, % people with university degrees | |
| Ichida et al., 2009[^147^](#bib147) | 2003 Aichi Gerontological Evaluation Study Project (AGES); Japan, 25 Kyuusons (communities) (Gini = 0.31). Sample: *n* = 15,225, age: 65--101. | Lower 2 of 4 (4,293 cases, 28.2% of total) | Individual-level: age, gender, equivalised annual income (before tax), marital status, educational attainment, type of housing  Area-level: average income, % trust | |
| Baum et al., 2016[^130^](#bib130) | 2006 Household Income and Labour Dynamics in Australia (HILDA) survey; Australia, Neighbourhoods^b^ (number of areas NR, Gini = 0.336). Sample: *n* = 1,680, age: 18--96. | Highest of 3^d^ (cases NR) | Individual-level: age, gender, low-income household, has university degree, employed/unemployed, overweight, obese, smoker, physically active  Area-level: NR | |
| Jen et al., 2009[^148^](#bib148) | 1981-2000 World Values Survey (WVS); Worldwide, 69 countries (Gini = 0.397). Sample: *n* = 171,264, age: 15--97. | Lower 1 of 3 (59,483 cases, 34.7% of total) | Individual-level: age, gender, individual income, marital status  Area-level: GDP | |
| Kahn et al., 2000[^149^](#bib149) | 1991 National Maternal Infant Health Survey (NMIHS); United States, 50 states (Gini = 0.42). Sample: *n* = 8,060, age: >15. | Lower 2 of 5 (781 cases, 9.7% of total) | Individual-level: age, individual income, education, marital status, race/ethnicity, household size  Area-level: NR | |
| Kennedy et al., 1998[^151^](#bib151) | 1993-1994 Behavioral Risk Factor Surveillance System (BRFSS); United States, 50 states (Gini = 0.332). Sample: *n* = 205,245, age: >18. | Lower 2 of 5 (29,679 cases, 14.5% of total) | Individual-level: age, gender, household income, educational attainment, race, health insurance status, recent use of healthcare services, household composition, smoking status, obesity  Area-level: NR | |
| Oshio and Kobayashi, 2009[^154^](#bib154) | 2004 Comprehensive Survey of Living Conditions of People on Health and Welfare (CSLCPHW); Japan, 47 prefectures (Gini = 0.355). Sample: *n* = 1,305, age: 20--89. | Lower 2 of 5 (299 cases, 22.9% of total) | Individual-level: age, gender, household income, education, marital status, smoking, drinking  Area-level: individual social capital, prefecture median income, % >65, prefecture social capital | |
| Qi, 2012[^155^](#bib155) | 2005 -2007 World Values Survey (WVS); Worldwide, 50 countries (Gini = 0.4). Sample: *n* = 61,756, age: 15--98. | Upper 2 of 5 (20,009 cases, 32.4% of total) | Individual-level: age, gender, household income, education, marital status  Area-level: GDP/capita, public health expenditure, % physicians, % immunization of measles, urban population | |
| Rostila et al., 2012[^156^](#bib156) | 2002 Stockholm County Council’s Public Health Questionnaire (PHQ); Sweden, 25 municipalities (Gini = 0.314). Sample: *n* = 31,182, age: 18--84. | Lower 2 of 5 (cases NR) | Individual-level: age, gender, family income, marital status  Area-level: average area income | |
| Wen et al., 2003[^163^](#bib163) | 1996, 1997, 1999 Metropolitan Chicago Information Center Metro Survey (MCIC-MS); United States, 275 neighbourhoods (Gini = 0.41). Sample: *n* = 3,459, age: >18. | Lower 2 of 4 (761 cases, 22.0% of total) | Individual-level: age, gender, annual household income, education level, marital status, race/ethnicity, smoking behaviour, high blood pressure, interview year  Area-level: neighbourhood health | |
| Wong et al., 2009[^164^](#bib164) | 2002 Thematic Household Surveys (THS); Hong Kong, 172 Tertiary Planning Unit (TPU) (Gini = 0.535). Sample: *n* = 24,610, age: >15. | Lower 2 of 5 (10,888 cases, 44.2% of total) | Individual-level: age group, gender, monthly household income, economic activity status, education, marital status  Area-level: NR | |
| Xi et al., 2005[^165^](#bib165) | 1996-1997 Ontario Health Survey (OHS), an expansion of the National Population Health Survey (NPHS); Canada, 42 public health unit (Gini = 0.37). Sample: *n* = 30,820, age: >25. | Lower 2 of 5 (3,942 cases, 12.8% of total) | Individual-level: age, gender, household income, educational level, marital status, regular exercise, smoking habits  Area-level: median area income | |
| Zheng, 2009[^166^](#bib166) | 1972-2004 General Social Survey (GSS); United States, 1 country (Gini = 0.429). Sample: *n* = 30,819, age: 18--89. | Lowest of 4^d^ (cases NR) | Individual-level: age in years, age squared, sex, household income, race, education, work status, marital status  Area-level: NR | |
| Bobak et al., 2007[^132^](#bib132) | 2004 New European Barometer (NEB); Central and Eastern Europe, 13 post-communist countries (Gini = 0.408). Sample: *n* = 5,330, age: >18. | Lower 2 of 5 (2,836 cases, 13.4% of total) | Individual-level: age, gender, quartile of household income, education, marital status, number of household items  Area-level: GDP | |
| Cai et al., 2020[^134^](#bib134) | 1991-2006 China Health and Nutrition Survey (CHNS); China, 9 provinces (Gini = 0.396). Sample: *n* = 40,514, age: >18. | Upper 2 of 4 (12,964 cases, 32.0% of total) | Individual-level: age, age squared, gender, household income (per capita), education level, work status, marital status, household size, smoking history, alcohol consumption, health insurance, availability of tap water, toilet types  Area-level: dummy variables of regional/temporal variation | |
| Chen and Gotway, 2012[^135^](#bib135) | 2000 Behavioural Risk Factor Surveillance System (BRFSS); United States, 52 states (incl. District of Columbia and Puerto Rico) (Gini = 0.442). Sample: *n =* 134,223, age: ≥18. | Upper 2 of 5 (57,582 cases, 42.9% of total) | Individual-level: age, gender, income, education, employment status, marital status, race/ethnicity  Area-level: county-level median household income, % in poverty, state, county inequalities, across-county income inequality | |
| Chiavegatto et al., 2012[^136^](#bib136) | 2000-2001 Health, Well-Being and Aging study; Brazil, 49 districts (Gini = 0.547). Sample: *n* = 2,143, age: >60. | Lower 1 of 5 (200 cases, 9.3% of total) | Individual-level: age, gender, individual income, education, BMI, regular physical exercise, smoking  Area-level: homicide rate | |
| Choi et al., 2015[^137^](#bib137) | 2000-2010 Health and Retirement Study (HRS); United States, 3,141 counties (Gini = 0.466). Sample: *n* = 34,994, age: >50. | Lower 2 of 5 (10,638 cases, 30.4% of total) | Individual-level: age, gender, wealth, income, education, marital status, race/ethnicity, years of living in/around current residence  Area-level: NR | |
| Critical risk of bias | | | |  |
| Fiscella and Franks, 2000[^141^](#bib141) | 1982-1984 National Health and Nutrition Examination Survey (NHANES); United States, 105 primary sampling units (Gini = 0.275). Sample: *n* = 13,280, age: 25--74. | Highest of 5 (cases NR) | Individual-level: age, gender, household income, family size  Area-level: NR | |
| Leclere and Soobader, 2000[^152^](#bib152) | 1989-1991 National Health Interview Survey (NHIS); United States, Counties^c^ (number of areas NR, Gini = 436). Sample: *n* = 161,711, age: >18. | Lower 2 of 5 (20,555 cases, 12.7% of total) | Individual-level: age (stratified), gender, logged family to income needs ratio, continuous years of schooling  Area-level: county poverty level | |
| Massa et al., 2018[^153^](#bib153) | 2013 National Health Survey (PNS); Brazil, 27 capital cities (Gini = 0.615). Sample: *n* = 27,017, age: >18. | Lower 2 of 5 (1,367 cases, 5.1% of total) | Individual-level: age, gender, educational attainment, marital status, race, smoking status, presence of hypertension, diabetes, hypercholesterolemia, depression  Area-level: per capita income, illiteracy rate, violence | |
| Bjornstrom and Medicine, 2011[^131^](#bib131) | 2000-2001 Los Angeles Family and Neighborhood Survey (L.A. FANS); Los Angeles, 65 census tracts (Gini = 0.43). Sample: *n* = 2,176, age: >18. | Lower 2 of 5 (479 cases, 22.0% of total) | Individual-level: age, gender, family income, education, currently working, relative position, marital status, race, recent move, parent, family size, BMI, current smoker, health insurance  Area-level: mortality rate, homicide rate | |
| Sommet and Elliot, 2022[^158^](#bib158) | 1972-2016 General Social Survey (GSS); United States, 279 counties (Gini = 0.45). Sample: *n* = 12,008, age: >18. | Highest of 3^d^ (1,495 cases, 12.5% of total) | Individual-level: age, gender, annual household income, years of education, race, employment status  Area-level: median household income, % in poverty, unemployment rate, % education < 9^th^ grade, population size | |
| Vauclair et al., 2015[^161^](#bib161) | 2008-2009 European Social Survey (ESS); Europe, 28 countries (Gini = 0.317). Sample: *n* = 7,819, age: >70. | Lowest of 5 (cases NR) | Individual-level: age, gender, subjective poverty, education  Area-level: NR | |
| Bobak et al., 2000[^133^](#bib133) | 1996-1998 New Democracies Barometer (NDB), New Baltic Barometer (NBB), and New Russia Barometer (NRB); Ex-communist countries, 7 post-communist countries (Gini = 0.353). Sample: *n* = 15,331, age: >20. | Lower 2 of 5 (713 cases, 18.5% of total) | Individual-level: age, gender, material deprivation, education, perceived control  Area-level: NR | |

NR = not reported, SRH = self-rated health
^a^Reported ordinal/number of SRH items

Appendix 16 Characteristics of Included Studies Reporting the Association Between Income Inequality and All-Cause Mortality

| **Author, year** | **Study information (study year/name, geographical scale, sample size and age)** | **Reported outcomes (follow-up time and no. of events)** | **Covariate adjustments for statistical model used in primary meta-analysis** |
| --- | --- | --- | --- |
| **Moderate risk of bias** | | | |
| Backlund et al., 2007[^168^](#bib168) | **1990 US National Longitudinal Mortality Study (NLMS); United States, 50 states** (Gini = 0.203)**.** Sample: ***n* = 521,248, age: >25.** | **Follow-up  =  8.4 years; No. of deaths  =  52,923, 10.2% of total** | **Individual-level: age (stratified), log of family income, household size, race, Hispanic ancestry, urbanization, marital status, education status, employment status  Area-level: region, % black** |
| Fiscella and Franks, 2000[^141^](#bib141) | **1971-1975 National Health and Nutrition Examination Survey (NHANES); United States, 105 primary sampling units** (Gini = 0.28)**.** Sample: ***n* = 13,280, age: 25--74.** | **Follow-up  =  9 years; No. of deaths  =  1,992, 15.0% of total** | **Individual-level: age, gender, household income, family size  Area-level: NR** |
| Henriksson et al., 2007[^171^](#bib171) | **1990 Sweden census; Sweden, 284 municipalities** (Gini = 0.231)**.** Sample: ***n* = 2,573,708, age: 40--64.** | **Follow-up  =  5 years; No. of deaths  =  108,120, 4.2% of total** | **Individual-level: age (categorical), gender, income distribution  Area-level: size of population in each municipality; % of manual/non-manual workers, mean income, % of poverty** |
| Kravdal, 2008[^173^](#bib173) | **1980-2002 Norway census; Norway, 431 municipalities** (Gini = 0.37)**.** Sample: ***n* = 2,500,000, age: 30--79.** | **Follow-up  =  11.5 years; No. of deaths  =  513,746, 0.9% of total** | **Individual-level: age (stratified), gender (stratified), income, education  Area-level: average income, average education, dummy variables for regional/temporal variation** |
| Ng et al., 2020[^175^](#bib175) | **1987-2004 Linnaeus Database; Sweden, 290 municipalities** (Gini = 0.293)**.** Sample: ***n* = 1,484,852, age: 65--92.** | **Follow-up  =  3 years; No. of deaths  =  344,999, 23.2% of total** | **Individual-level: age (stratified), year of birth, disposable income, level of education, marital status  Area-level: median municipality income, region type** |
| Zhao et al., 2021[^178^](#bib178) | **1968-1997 Panel Study of Income Dynamics (PSID); United States, 50 states** (Gini = 0.38)**.** Sample: ***n =* 4,774, age: >45.** | **Follow-up  =  10.16 years;  No. of deaths  =  213, 4.5% of total** | **Individual-level: age (birth cohort), gender, family income, race, educational attainment, employment status  Area-level: region of birth, mean neighbourhood family income, % of Black residents, population size** |
| Zheng, 2012[^179^](#bib179) | **1986-2004 National Health Interview Survey (NHIS); United States, 50 states** (Gini = 0.339)**.** Sample: ***n* = 701,179, age: >30.** | **Follow-up  =  11 years; No. of deaths  =  125,391, 17.9% of total** | **Individual-level: age gender, family income adjusted for inflation, years of formal education, work status, marital status, race, survey year  Area-level: GDP per capita** |
| **Serious risk of bias** | | | |
| Auger et al., 2012[^167^](#bib167) | **1991 Canadian Census Mortality Follow-up Study (CCMFS); Canada, 140 urban areas** (Gini = 0.323)**.** Sample: ***n =* 2,077,000, age: >25.** | **Follow-up  =  5.8 years; No. of deaths  =  202,354, 9.7% of total** | **Individual-level: age, gender (stratified), income, education, employment, marital status, immigration, visible minority  Area-level: mean area household income, region** |
| Blakely et al., 2003[^169^](#bib169) | **1991-1994 New Zealand census; New Zealand, 35 sub-regions** (Gini = 0.341)**.** Sample: ***n* = 1,391,118, age: 25--64.** | **Follow-up  =  2 years; No. of deaths  =  10,299, 0.7% of total** | **Individual-level: age, gender (stratified), household income (equivalised), ethnicity  Area-level: rurality, regional income** |
| Gerdtham and Johannesson, 2004[^170^](#bib170) | **1980-1996 Statistic Sweden’s Survey of Living Conditions (ULF study); Sweden, 284 municipalities** (Gini = 0.183)**.** Sample: ***n* = 41,006, age: 20--84.** | **Follow-up  =  13 years; No. of deaths  =  6,725, 16.4% of total** | **Individual-level: age, gender, income, education, unemployment, immigration, marital status, number of children, dummy variable for inclusion into study  Area-level: mean income** |
| Kimmel et al., 2013[^172^](#bib172) | **2000 U.S. Renal Data System (USRDS); United States, Metropolitan statistical areas^c^ (number of areas NR, Gini NR).** Sample: ***n* = 623,949, age: >20.** | **Follow-up  =  1.9 years; No. of deaths  =  333,427, 53.4% of total** | **Individual-level: age, gender, individual income, employment status, race, year of dialysis initiation, BMI, smoking status, insurance, primary cause of renal failure, comorbid conditions  Area-level: residential segregation** |
| Modrek et al., 2012[^174^](#bib174) | **1989 Costa Rican Longitudinal Mortality Study (CR-LMS); Costa Rica, 81 cantons** (Gini = 0.374)**.** Sample: ***n =* 15,276, age: >18.** | **Follow-up  =  9.5 years; No. of deaths  =  3,746, 24.5% of total** | **Individual-level: age, age squared, age cubed, wealth, education, marital status, health insurance status, living in the San José metropolitan, living in an urban area  Area-level: mean monthly household income, % unemployed (1989), % in migration (1989)** |
| Osler et al., 2002[^176^](#bib176) | **1976-8/ 1964--92 Copenhagen City Heart Study (CCHS), Glostrup Population Study (GPS); Copenhagen, 153 parishes** (Gini NR)**.** Sample: ***n* = 25,728, age: >15.** | **Follow-up  =  12.8 years; No. of deaths  =  7,567, 29.4% of total** | **Individual-level: age, gender (stratified), individual income, education, household structure, smoking status, physical activity, BMI, alcohol intake  Area-level: % of cohabiting partners with children <18** |
| Pabayo et al., 2013[^177^](#bib177) | **2000 Health, Well-Being, and Aging (SABE) Survey; Brazil, 72 census tracts** (Gini = 0.27)**.** Sample: ***n* = 1,024, age: ≥ 60.** | **Follow-up  =  4 years; No. of deaths  =  89, 8.7% of total** | **Individual-level: age, gender, income, education  Area-level: % of poverty** |

NR = not reported
^a^Mean/median Gini coefficient: <0.30

^b^Mean/median Gini coefficient: 0.30-0.40
^c^Mean/median Gini coefficient: >0.40

Appendix 17 Critical Appraisal Results of Studies Considering Self-Rated Health Using ROBINS-I (Interrater Reliability = 67.6%)


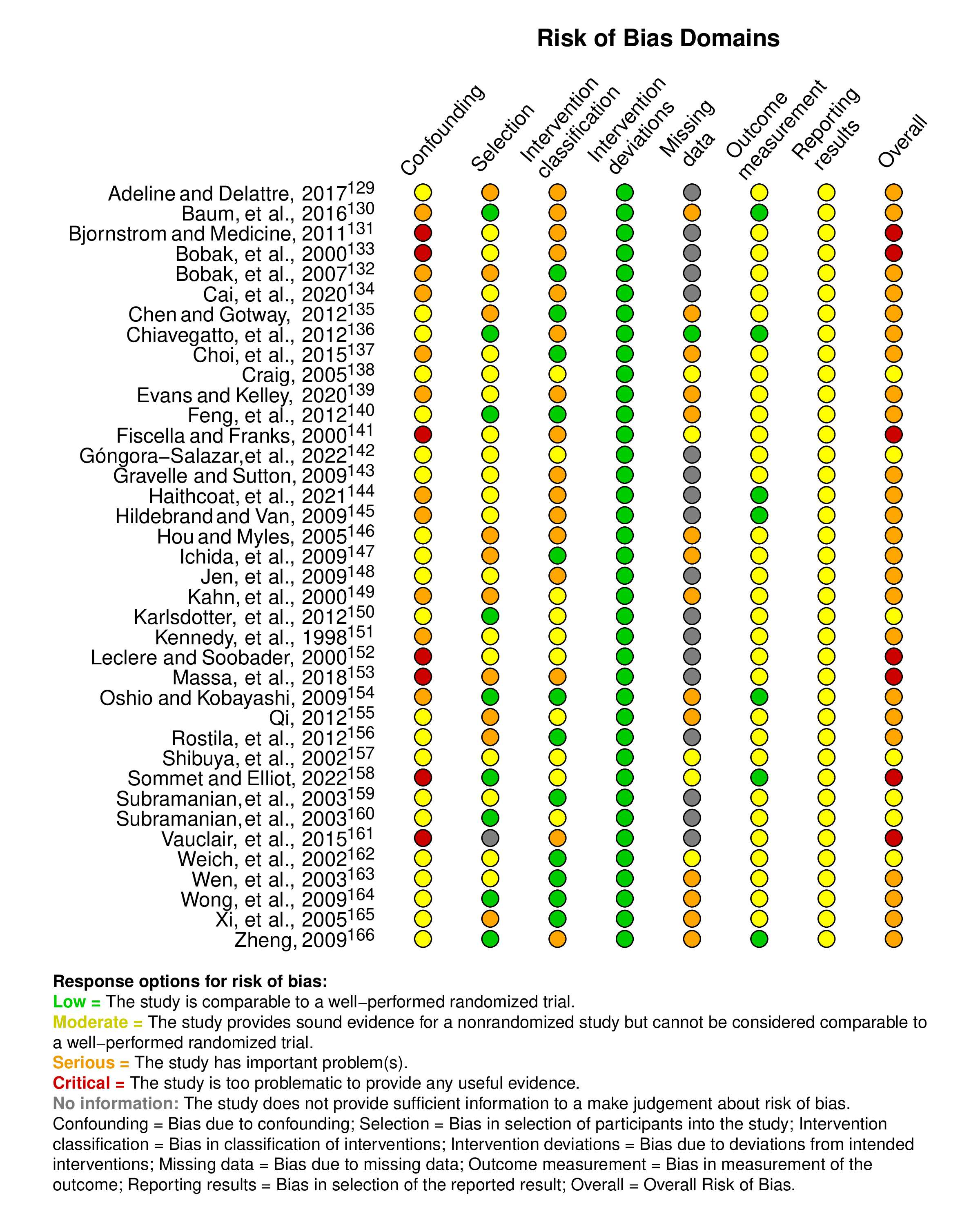


Appendix 18 Critical Appraisal Results for Studies Considering All-Cause Mortality Using ROBINS-I (Interrater Reliability: 69.2%)


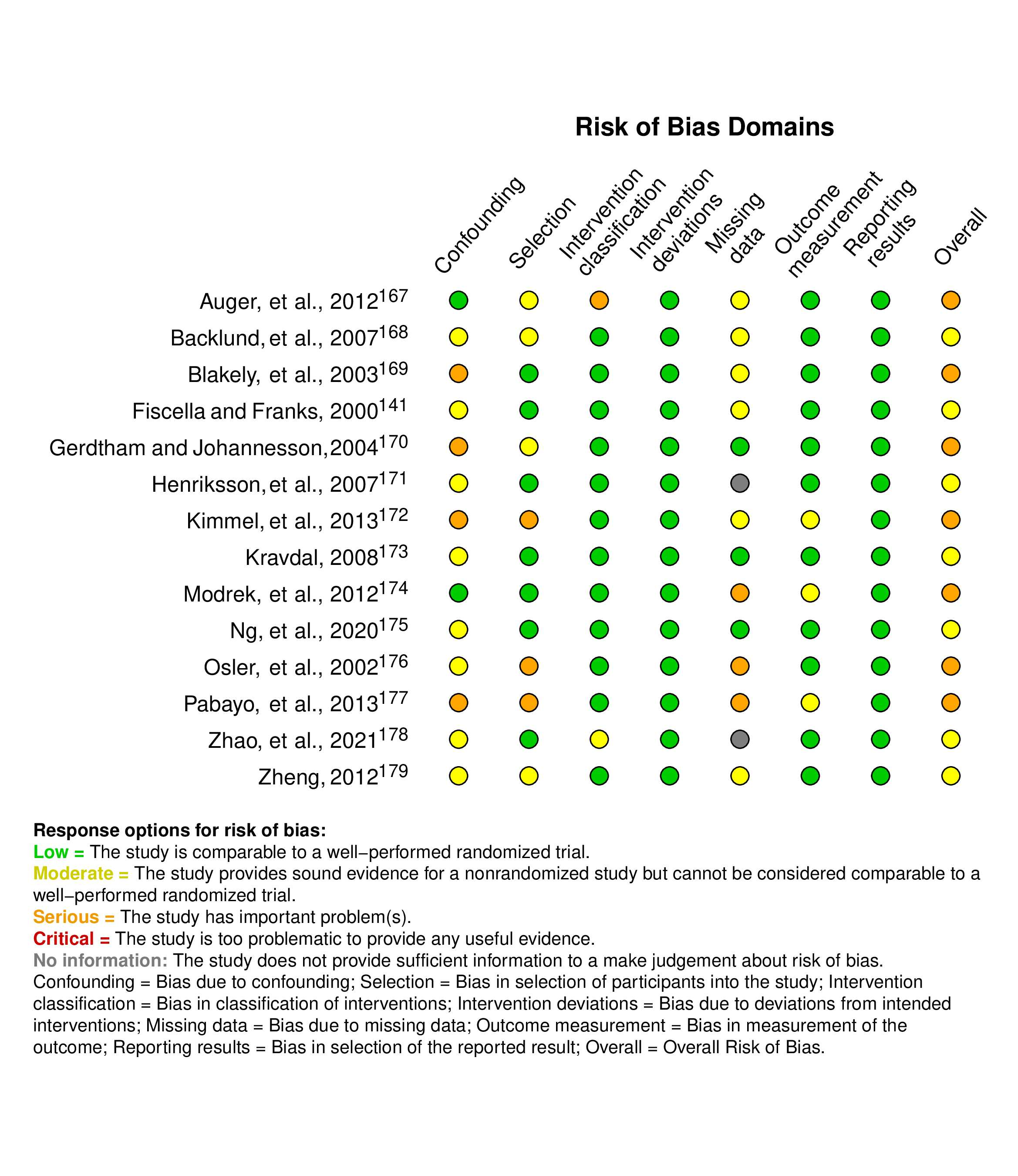


Appendix 19 Self-Rated Health Subgroup Analyses, Odds Ratio (95% Confidence Interval) per 0.05-Unit Increase in Gini Coefficient

| **Covariates** | **OR (95% CI)**[**^a^**](#tbfnafn1) | | **ꭓ^2b^** | | **p value^c^** | |
| --- | --- | --- | --- | --- | --- | --- |
| **Geographical scale**[**^d^**](#tbfnafn3) | | | | | | |
| Local level, within-country (*n*  =  20[^130^](#bib130)^,^ [^131^](#bib131)^,^ [^134^](#bib134)^-^ [^138^](#bib138)^,^ [^141^](#bib141)^,^ [^146^](#bib146)^,^ [^147^](#bib147)^,^ [^152^](#bib152)^,^ [^153^](#bib153)^,^ [^156^](#bib156)^-^ [^158^](#bib158)^,^ [^160^](#bib160)^,^ [^163^](#bib163)^-^ [^165^](#bib165)^,^ [^180^](#bib180)) | | 1.03 (1.01 – 1.06) | 6.17 | | 0.046 | |
| Regional level, within-country (*n*  =  15[^134^](#bib134)^,^ [^135^](#bib135)^,^ [^140^](#bib140)^,^ [^142^](#bib142)^-^ [^145^](#bib145)^,^ [^149^](#bib149)^-^ [^151^](#bib151)^,^ [^154^](#bib154)^,^ [^156^](#bib156)^,^ [^159^](#bib159)^,^ [^162^](#bib162)^,^ [^166^](#bib166)) | | 1.09 (1.05 – 1.14) |  | |  | |
| National level, among-country (*n*  =  8[^129^](#bib129)^,^ [^132^](#bib132)^,^ [^133^](#bib133)^,^ [^139^](#bib139)^,^ [^145^](#bib145)^,^ [^148^](#bib148)^,^ [^155^](#bib155)^,^ [^161^](#bib161)) | | 1.04 (1.00 – 1.08) |  | |  | |
| **Mean income inequality**[**^e^**](#tbfnafn4) | | | | | | |
| <0.30 (*n*  =  5[^138^](#bib138)^,^ [^141^](#bib141)^,^ [^145^](#bib145)^,^ [^150^](#bib150)^,^ [^162^](#bib162)) | 1.03 (1.01 – 1.05) | | 2.64 | | 0.27 | |
| 0.30-0.40 (*n*  =  16[^129^](#bib129)^,^ [^130^](#bib130)^,^ [^133^](#bib133)^,^ [^134^](#bib134)^,^ [^139^](#bib139)^,^ [^143^](#bib143)^,^ [^146^](#bib146)^-^ [^148^](#bib148)^,^ [^151^](#bib151)^,^ [^154^](#bib154)^-^ [^157^](#bib157)^,^ [^161^](#bib161)^,^ [^165^](#bib165)) | 1.07 (1.02 – 1.12) | |  | |  | |
| >0.40 (*n*  =  17[^131^](#bib131)^,^ [^132^](#bib132)^,^ [^135^](#bib135)^-^ [^137^](#bib137)^,^ [^140^](#bib140)^,^ [^142^](#bib142)^,^ [^144^](#bib144)^,^ [^149^](#bib149)^,^ [^152^](#bib152)^,^ [^153^](#bib153)^,^ [^158^](#bib158)^-^ [^160^](#bib160)^,^ [^163^](#bib163)^,^ [^164^](#bib164)^,^ [^166^](#bib166)) | 1.05 (1.02 – 1.08) | |  | |  | |
| **World region**[**^f^**](#tbfnafn5) | | | | | | |
| Asia and Pacific (*n*  =  7[^130^](#bib130)^,^ [^134^](#bib134)^,^ [^140^](#bib140)^,^ [^147^](#bib147)^,^ [^154^](#bib154)^,^ [^157^](#bib157)^,^ [^164^](#bib164)) | 1.10 (1.03 – 1.17) | | 9.03 | | 0.06 | |
| Europe and Central Asia (*n*  =  11[^129^](#bib129)^,^ [^132^](#bib132)^,^ [^133^](#bib133)^,^ [^138^](#bib138)^,^ [^139^](#bib139)^,^ [^143^](#bib143)^,^ [^145^](#bib145)^,^ [^150^](#bib150)^,^ [^156^](#bib156)^,^ [^161^](#bib161)^,^ [^162^](#bib162)) | 1.06 (1.02 – 1.09) | |  | |  | |
| Latin America and the Caribbean (*n*  =  4[^136^](#bib136)^,^ [^142^](#bib142)^,^ [^153^](#bib153)^,^ [^160^](#bib160)) | 1.10 (1.03 – 1.17) | |  | |  | |
| North America (*n*  =  14[^131^](#bib131)^,^ [^135^](#bib135)^,^ [^137^](#bib137)^,^ [^141^](#bib141)^,^ [^144^](#bib144)^,^ [^146^](#bib146)^,^ [^149^](#bib149)^,^ [^151^](#bib151)^,^ [^152^](#bib152)^,^ [^158^](#bib158)^,^ [^159^](#bib159)^,^ [^163^](#bib163)^,^ [^165^](#bib165)^,^ [^166^](#bib166)) | 1.04 (1.01 – 1.07) | |  | |  | |
| Multiple world regions (*n*  =  2[^148^](#bib148)^,^ [^155^](#bib155)) | 0.98 (0.92 – 1.04) | |  | |  | |
| **Study setting** | | | | | | |
| Not United States (*n*  =  28[^129^](#bib129)^,^ [^130^](#bib130)^,^ [^132^](#bib132)^--^[^134^](#bib134)^,^ [^136^](#bib136)^--^[^143^](#bib143)^,^ [^145^](#bib145)^--^[^148^](#bib148)^,^ [^150^](#bib150)^,^ [^153^](#bib153)^--^[^157^](#bib157)^,^ [^160^](#bib160)^-^ [^162^](#bib162)^,^ [^164^](#bib164)^,^ [^165^](#bib165)) | 1.06 (1.03 – 1.09) | | 0.12 | | 0.73 | |
| United States (*n*  =  10[^131^](#bib131)^,^ [^135^](#bib135)^,^ [^144^](#bib144)^,^ [^149^](#bib149)^,^ [^151^](#bib151)^,^ [^152^](#bib152)^,^ [^158^](#bib158)^,^ [^159^](#bib159)^,^ [^163^](#bib163)^,^ [^166^](#bib166)) | 1.05 (1.01 – 1.09) | |  | |  | |
| **Average population age**[**^g^**](#tbfnafn6) | | | | | | |
| ≤60 years old (*n*  =  33[^129^](#bib129)^-^[^135^](#bib135)^,^ [^138^](#bib138)^,^ [^139^](#bib139)^,^ [^141^](#bib141)^--^[^146^](#bib146)^,^ [^148^](#bib148)^-^ [^160^](#bib160)^,^ [^162^](#bib162)^--^[^166^](#bib166)) | 1.06 (1.03 – 1.09) | | 0.34 | | 0.56 | |
| >60 years old (*n*  =  7[^136^](#bib136)^,^ [^137^](#bib137)^,^ [^140^](#bib140)^,^ [^147^](#bib147)^,^ [^152^](#bib152)^,^ [^161^](#bib161)^,^ [^182^](#bib182)) | 1.09 (0.99 – 1.20) | |  | |  | |
| **Covariate adjustments**[**^h^**](#tbfnafn7) | | | | | | |
| Individual-level variables only (*n*  =  23[^130^](#bib130)^,^ [^132^](#bib132)^,^ [^133^](#bib133)^,^ [^136^](#bib136)^,^ [^137^](#bib137)^,^ [^140^](#bib140)^,^ [^141^](#bib141)^,^ [^145^](#bib145)^,^ [^146^](#bib146)^,^ [^149^](#bib149)^-^ [^151^](#bib151)^,^ [^153^](#bib153)^--^[^157^](#bib157)^,^ [^161^](#bib161)^,^ [^162^](#bib162)^,^ [^164^](#bib164)^--^[^166^](#bib166)^,^ [^184^](#bib184)) | 1.06 (1.04 – 1.09) | | 0.069 | | 0.79 | |
| With area-level variables (*n*  =  28[^129^](#bib129)^,^ [^131^](#bib131)^,^ [^132^](#bib132)^,^ [^134^](#bib134)^--^[^136^](#bib136)^,^ [^138^](#bib138)^,^ [^139^](#bib139)^,^ [^142^](#bib142)^--^[^144^](#bib144)^,^ [^146^](#bib146)^--^[^148^](#bib148)^,^ [^152^](#bib152)^--^[^160^](#bib160)^,^ [^163^](#bib163)^,^ [^165^](#bib165)^,^ [^182^](#bib182)^,^ [^184^](#bib184)^,^ [^185^](#bib185)) | 1.07 (1.03 – 1.11) | |  | |  | |
| **Measure of income distribution** |  | |  | |  | |
| Gini coefficient (*n =* 35[^131^](#bib131)^--^[^140^](#bib140)^,^ [^142^](#bib142)^--^[^166^](#bib166)) | 1.06 (1.03 – 1.08) | | 0.51 | | 0.82 | |
| Non-Gini coefficient (*n =* 3[^129^](#bib129)^,^ [^130^](#bib130)^,^ [^141^](#bib141)) | 1.06 (0.99 – 1.15) | |  | |  | |
| **Risk of bias** | | | | | | |
| Moderate (*n*  =  7[^138^](#bib138)^,^ [^142^](#bib142)^,^ [^150^](#bib150)^,^ [^157^](#bib157)^,^ [^159^](#bib159)^,^ [^160^](#bib160)^,^ [^162^](#bib162)) | 1.05 (1.03 – 1.06) | | 1.36 | | 0.51 | |
| Serious (*n*  =  24[^129^](#bib129)^,^ [^130^](#bib130)^,^ [^132^](#bib132)^,^ [^134^](#bib134)^--^[^137^](#bib137)^,^ [^139^](#bib139)^,^ [^140^](#bib140)^,^ [^143^](#bib143)^--^[^149^](#bib149)^,^ [^151^](#bib151)^,^ [^154^](#bib154)^--^[^156^](#bib156)^,^ [^163^](#bib163)^-^ [^166^](#bib166)) | 1.07 (1.03 – 1.10) | |  | |  | |
| Critical (*n*  =  7[^131^](#bib131)^,^ [^133^](#bib133)^,^ [^141^](#bib141)^,^ [^152^](#bib152)^,^ [^153^](#bib153)^,^ [^158^](#bib158)^,^ [^161^](#bib161)) | 1.04 (1.00 – 1.08) | |  | |  | |
| **Time lag^‡‡^** | | | | | | |
| ≤6 years (*n*  =  37[^129^](#bib129)^-^ [^162^](#bib162)^,^ [^164^](#bib164)^--^[^166^](#bib166)) | 1.05 (1.03 – 1.08) | | 0.69 | | 0.41 | |
| >6 years (*n*  =  6[^140^](#bib140)^,^ [^150^](#bib150)^,^ [^159^](#bib159)^,^ [^163^](#bib163)^,^ [^181^](#bib181)^,^ [^183^](#bib183)) | 1.08 (1.03 – 1.13) | |  | |  | |

^a^Odds ratio (OR) (95% confidence intervals (CI)) for each subgroup reflects change in poor self-rated health (SRH) per 0.05-unit increase in Gini coefficient. Calculated using random effects models with restricted maximum likelihood estimate.

^b^χ2 and corresponding ^c^p value evaluating between group differences, which should be interpreted with caution when there are few studies in each subgroup. For testing difference between null hypothesis (coefficient has no effect) compared to coefficient affects estimate.

^d^Studies reporting estimates with multiple geographical areas: China Health and Nutrition Survey (CHNS)[^134^](#bib134); Behavioral Risk Factor Surveillance System (BRFSS)[^135^](#bib135); European Community Household Panel survey (ECHP)[^145^](#bib145); Departamento Administrativo Nacional de Estadística (DANE)[^180^](#bib180); Stockholm County Council’s Public Health Questionnaire (PHQ).[^156^](#bib156)

^e^Based on categories from Lin et al., 2017.[^51^](#bib51)

^f^Based on World Bank classifications.[^52^](#bib52)

^g^Studies reporting estimates with both levels of average age: National Health Survey (PNS).[^153^](#bib153)^,^ [^182^](#bib182)

^h^Studies reporting estimates with individual-level only and individual and area-level variable adjustments: New European Barometer (NEB)[^132^](#bib132); Health, Well-Being and Aging study[^136^](#bib136); Comprehensive Survey of Living Conditions of People on Health and Welfare (CSLCPHW)[^154^](#bib154); World Values Survey (WVS)[^155^](#bib155); Behavioral Risk Factor Surveillance System (BRFSS)[^185^](#bib185); Current Population Survey (CPS)[^184^](#bib184); Canadian National Population Health Survey (CNPHS)[^146^](#bib146); National Health Survey (PNS)[^153^](#bib153); National Health Survey (PNS)[^182^](#bib182); Stockholm County Council’s Public Health Questionnaire (PHQ)[^156^](#bib156); Comprehensive Survey of Living Conditions of People on Health and Welfare (CSLCPHW)[^157^](#bib157); Ontario Health Survey (OHS), an expansion of the National Population Health Survey (NPHS).[^165^](#bib165)

^i^Studies reporting estimates with ≤ 6 year time lag and >6 year time lag: Chinese Longitudinal Healthy Longevity Survey (CLHLS)[^140^](#bib140); Life Conditions Survey (LCS)[^150^](#bib150); Canadian National Population Health Survey (CNPHS)[^181^](#bib181); China Health and Nutrition Survey (CHNS)[^183^](#bib183); Current Population Survey (CPS).[^159^](#bib159)

Appendix 20 All-Cause Mortality Subgroup Analyses, Risk Ratio (95% Confidence Interval) per 0.05-unit Increase in Gini Coefficient

| **Covariates** | **RR (95% CI)**[**^a^**](#tbfnafn1) | **ꭓ^2b^** | **p value^c^** |
| --- | --- | --- | --- |
| **Geographical scale**[**^d^**](#tbfnafn3) | | | |
| Local level, within-country (*n*  =  11[^167^](#bib167)^,^ [^170^](#bib170)^--^[^178^](#bib178)^,^ [^141^](#bib141)) | 1.00 (0.99 – 1.02) | 2.64 | 0.10 |
| Regional level, within-country (*n*  =  4[^168^](#bib168)^,^ [^169^](#bib169)^,^ [^178^](#bib178)^,^ [^179^](#bib179)) | 1.09 (0.99 – 1.21) |  |  |
| **Mean income inequality**[**^e^**](#tbfnafn4) | | | |
| <0.30 Gini coefficient (*n*  =  7[^168^](#bib168)^,^ [^170^](#bib170)^,^ [^171^](#bib171)^,^ [^175^](#bib175)^-^ [^177^](#bib177)^,^ [^141^](#bib141)) | 1.01 (0.99 – 1.03) | 0.58 | 0.75 |
| 0.30-0.40 Gini coefficient (*n*  =  6[^167^](#bib167)^,^ [^169^](#bib169)^,^ [^173^](#bib173)^,^ [^174^](#bib174)^,^ [^178^](#bib178)^,^ [^179^](#bib179)) | 1.03 (0.98 – 1.08) |  |  |
| >0.40 Gini coefficient (*n*  =  1[^172^](#bib172)) | 1.01 (1.01 – 1.02) |  |  |
| **World region**[**^f^**](#tbfnafn5) | | | |
| Asia and Pacific (*n*  =  1[^169^](#bib169)) | 1.03 (0.96 – 1.10) | 4.7 | 0.20 |
| Europe and Central Asia (*n*  =  5[^170^](#bib170)^,^ [^171^](#bib171)^,^ [^173^](#bib173)^,^ [^175^](#bib175)^,^ [^176^](#bib176)) | 0.99 (0.98 – 1.01) |  |  |
| Latin America and the Caribbean (*n*  =  2[^174^](#bib174)^,^ [^177^](#bib177)) | 1.03 (0.89 – 1.18) |  |  |
| North America (*n*  =  6[^167^](#bib167)^,^ [^168^](#bib168)^,^ [^172^](#bib172)^,^ [^178^](#bib178)^,^ [^179^](#bib179)^,^ [^141^](#bib141)) | 1.07 (1.00 – 1.14) |  |  |
| **Study setting** | | | |
| Not United States (*n*  =  8[^167^](#bib167)^,^ [^170^](#bib170)^,^ [^171^](#bib171)^,^ [^173^](#bib173)^-^ [^177^](#bib177)) | 1.00 (0.98 – 1.01) | 3.55 | 0.06 |
| United States (*n*  =  6[^168^](#bib168)^,^ [^169^](#bib169)^,^ [^172^](#bib172)^,^ [^178^](#bib178)^,^ [^179^](#bib179)^,^ [^141^](#bib141)) | 1.08 (1.00 – 1.17) |  |  |
| **Average population age**[**^g^**](#tbfnafn6) | | | |
| ≤60 years old (*n*  =  12[^167^](#bib167)^-^[^174^](#bib174)^,^ [^176^](#bib176)^,^ [^178^](#bib178)^,^ [^179^](#bib179)^,^ [^141^](#bib141)) | 1.04 (1.00 – 1.08) | 7.53 | 0.0061 |
| >60 years old (*n*  =  5[^167^](#bib167)^,^ [^168^](#bib168)^,^ [^173^](#bib173)^,^ [^175^](#bib175)^,^ [^177^](#bib177)) | 0.98 (0.97 – 1.00) |  |  |
| **Covariate adjustments**[**^h^**](#tbfnafn7) | | | |
| Individual-level variables only (*n*  =  7[^167^](#bib167)^,^ [^168^](#bib168)^,^ [^172^](#bib172)^,^ [^174^](#bib174)^,^ [^175^](#bib175)^,^ [^178^](#bib178)^,^ [^141^](#bib141)) | 1.03 (0.98 – 1.08) | 1.05 | 0.3 |
| With area-level variables (*n*  =  11[^167^](#bib167)^-^[^171^](#bib171)^,^ [^173^](#bib173)^--^[^177^](#bib177)^,^ [^179^](#bib179)) | 1.01 (0.99 – 1.02) |  |  |
| **Measure of income distribution** |  |  |  |
| Gini coefficient (*n =* 11[^167^](#bib167)^--^[^175^](#bib175)^,^ [^177^](#bib177)^,^ [^178^](#bib178)^,^ [^187^](#bib187)) | 1.02 (0.99 – 1.05) | 0.52 | 0.47 |
| Non-Gini coefficient (*n =* 3[^168^](#bib168)^,^ [^176^](#bib176)^,^ [^179^](#bib179)) | 1.03 (1.00 – 1.07) |  |  |
| **Risk of bias** | | | |
| Moderate (*n*  =  7[^168^](#bib168)^,^ [^171^](#bib171)^,^ [^173^](#bib173)^,^ [^175^](#bib175)^,^ [^178^](#bib178)^,^ [^179^](#bib179)^,^ [^141^](#bib141)) | 1.02 (0.99 – 1.06) | 0.62 | 0.43 |
| Serious (*n*  =  7[^167^](#bib167)^,^ [^169^](#bib169)^,^ [^170^](#bib170)^,^ [^172^](#bib172)^,^ [^174^](#bib174)^,^ [^176^](#bib176)^,^ [^177^](#bib177)) | 1.01 (1.00 to 1.02) |  |  |
| **Time lag**[**^i^**](#tbfnafn8) | | | |
| ≤6 years (*n*  =  13[^167^](#bib167)^-^[^169^](#bib169)^,^ [^171^](#bib171)^--^[^179^](#bib179)^,^ [^141^](#bib141)) | 1.02 (1.00 – 1.05) | 4.26 | 0.039 |
| >6 years (*n*  =  4[^170^](#bib170)^,^ [^173^](#bib173)^,^ [^175^](#bib175)^,^ [^179^](#bib179)) | 0.98 (0.96 – 1.01) |  |  |
| **Follow-up time** | | | |
| <5 years (*n*  =  4[^169^](#bib169)^,^ [^172^](#bib172)^,^ [^175^](#bib175)^,^ [^177^](#bib177)) | 0.99 (0.97 – 1.00) | 7.86 | 0.02 |
| >10 years (*n*  =  5[^170^](#bib170)^,^ [^173^](#bib173)^,^ [^176^](#bib176)^,^ [^178^](#bib178)^,^ [^179^](#bib179)) | 1.03 (0.97 – 1.09) |  |  |
| 5-10 years (*n*  =  5[^167^](#bib167)^,^[^168^](#bib168)^,^ [^171^](#bib171)^,^ [^174^](#bib174)^,^ [^141^](#bib141)) | 1.02 (1.00 – 1.05) |  |  |

^a^Risk ratio (RR) (95% CI) for each subgroup reflects change in all-cause mortality per 0.05-unit increase in Gini coefficient. Calculated using random effects models with restricted maximum likelihood estimate.

^b^χ2 and corresponding ^c^p value evaluating between group differences, which should be interpreted with caution when there are few studies in each subgroup. For testing difference between null hypothesis (coefficient has no effect) compared to coefficient affects estimate.

^d^Panel Study of Income Dynamics (PSID).[^178^](#bib178)

^e^Based on categories from Lin et al., 2017[^51^](#bib51)

^f^Based on World Bank classifications.[^52^](#bib52)

^g^Studies reporting estimates with both levels of average age: Canadian Census Mortality Follow-up Study (CCMFS)[^167^](#bib167); US National Longitudinal Mortality Study (NLMS)[^168^](#bib168); Norway census.[^173^](#bib173)

^h^Studies reporting estimates with individual-level only and individual and area-level variable adjustments: Canadian Census Mortality Follow-up Study (CCMFS)[^167^](#bib167); United States National Longitudinal Mortality Study (NLMS)[^168^](#bib168); Costa Rican Longitudinal Mortality Study (CR-LMS)[^174^](#bib174); Linnaeus Database.[^175^](#bib175)

^i^Studies reporting estimates with a time lag <6 year and > 6 year: Norway census[^173^](#bib173); National Health Interview Survey (NHIS)[^179^](#bib179); Linnaeus Database.[^175^](#bib175)

Appendix 21 Sensitivity Analysis Results for (a) Self-Rated Health and (b) All-Cause Mortality

| **(A) Self-rated health sensitivity analyses** | **Odds ratio (95% CI)**[**^a^**](#tbfnafn1) |
| --- | --- |
| **Primary analysis** (*n*  =  38[^129^](#bib129)^-^[^166^](#bib166)) | 1.06 (1.03 – 1.08) |
| Including studies with non-Gini coefficient estimates (*n*  =  39[^129^](#bib129)^-^[^166^](#bib166)^,^ [^186^](#bib186)) | 1.06 (1.03 – 1.08) |
| Excluding studies at critical risk of bias (*n*  =  31[^129^](#bib129)^,^ [^130^](#bib130)^,^ [^132^](#bib132)^,^ [^134^](#bib134)^--^[^140^](#bib140)^,^ [^142^](#bib142)^,^ [^151^](#bib151)^,^ [^154^](#bib154)^,^ [^157^](#bib157)^,^ [^159^](#bib159)^,^ [^160^](#bib160)^,^ [^162^](#bib162)^,^ [^166^](#bib166)) | 1.06 (1.04 – 1.09) |
| Excluding ordinal outcomes (*n*  =  31[^131^](#bib131)^-^[^138^](#bib138)^,^ [^140^](#bib140)^,^ [^142^](#bib142)^,^ [^144^](#bib144)^,^ [^145^](#bib145)^,^ [^147^](#bib147)^,^ [^157^](#bib157)^,^ [^159^](#bib159)^,^ [^165^](#bib165)) | 1.05 (1.03 – 1.08) |
| Excluding all non-Gini coefficient estimates (*n*  =  35[^131^](#bib131)^-^[^140^](#bib140)^,^ [^142^](#bib142)^,^ [^166^](#bib166)) | 1.06 (1.03 – 1.08) |
| **Multiple observations** |  |
| Geographical scale[^b^](#tbfnafn2) (*n*  =  38[^129^](#bib129)^-^[^141^](#bib141)^,^ [^143^](#bib143)^,^ [^180^](#bib180)) | 1.05 (1.03 – 1.07) |
| Covariate adjustments[^c^](#tbfnafn3) (*n*  = 37[^129^](#bib129)^--^[^140^](#bib140)^,^ [^142^](#bib142)^,^ [^158^](#bib158)^,^ [^160^](#bib160)^,^ [^166^](#bib166)^,^ [^184^](#bib184)) | 1.06 (1.04 – 1.09) |
| Time lag[^d^](#tbfnafn4) (*n*  =  38[^129^](#bib129)^-^[^133^](#bib133)^,^ [^135^](#bib135)^,^ [^145^](#bib145)^,^ [^147^](#bib147)^,^ [^166^](#bib166)^,^ [^181^](#bib181)^,^ [^183^](#bib183)) | 1.05 (1.03 – 1.07) |
| **(B) All-cause mortality sensitivity analyses** | **Risk ratio (95% CI)**[**^a^**](#tbfnafn1) |
| **Primary analysis** (*n*  = 14[^167^](#bib167)^--^[^179^](#bib179)^,^ [^141^](#bib141)) | 1.02 (1.00 – 1.04) |
| Including studies that do not address clustering or time dummy variables[^e^](#tbfnafn5) (*n*  = 16[^167^](#bib167)^--^[^179^](#bib179)^,^ [^189^](#bib189)^,^ [^192^](#bib192)^,^ [^141^](#bib141)) | 1.02 (1.00 – 1.05) |
| Including abstract-only studies (*n*  =  15[^167^](#bib167)^-^[^179^](#bib179)^,^ [^193^](#bib193)^,^ [^141^](#bib141)) | 1.02 (1.00 – 1.05) |
| Excluding studies with non-Gini coefficient estimates (*n*  =  11[^167^](#bib167)^,^ [^169^](#bib169)^,^ [^175^](#bib175)^,^ [^177^](#bib177)^,^ [^179^](#bib179)) | 1.02 (0.99 – 1.05) |
| **Multiple observations** |  |
| Geographical scale[^b^](#tbfnafn2) (*n*  =  14[^167^](#bib167)^-^[^172^](#bib172)^,^ [^174^](#bib174)^,^ [^179^](#bib179)^,^ [^188^](#bib188)^,^ [^141^](#bib141)) | 1.02 (1.00 – 1.04) |
| Covariate adjustments[^c^](#tbfnafn3) (*n*  =  13[^167^](#bib167)^-^[^172^](#bib172)^,^ [^174^](#bib174)^,^ [^179^](#bib179)^,^ [^190^](#bib190)) | 1.03 (1.00 – 1.06) |
| Time lag[^d^](#tbfnafn4) (*n*  =  14[^167^](#bib167)^-^ [^179^](#bib179)^,^ [^141^](#bib141)) | 1.02 (0.99 – 1.05) |
| Different Swedish census sample[^f^](#tbfnafn6) (*n*  =  14[^167^](#bib167)^-^[^170^](#bib170)^,^ [^172^](#bib172)^,^ [^179^](#bib179)^,^ [^192^](#bib192)^,^ [^141^](#bib141)) | 1.02 (0.99 – 1.05) |
| Refugee sample from Swedish census[^g^](#tbfnafn7) (*n =* 14[^167^](#bib167)^--^[^170^](#bib170)^,^ [^172^](#bib172)^,^ [^179^](#bib179)^,^ [^191^](#bib191)^,^ [^141^](#bib141)) | 1.02 (0.99 – 1.05) |

^a^Odds ratio (OR) (95% confidence intervals (CI)) for each analysis reflects change in poor self-rated health (SRH) per 0.05-unit increase in Gini coefficient. Risk ratio (RR) (95% CI) for each analysis reflects change in all-cause mortality per 0.05-unit increase in Gini coefficient. Calculated using random effects models with restricted maximum likelihood estimate.

^b^Studies with multiple observations by geographical scale considering self-rated health: Behavioral Risk Factor Surveillance System (BRFSS)[^135^](#bib135); China Health and Nutrition Survey (CHNS)[^134^](#bib134); Departamento Administrativo Nacional de Estadística (DANE)[^180^](#bib180); European Community Household Panel survey (ECHP)[^145^](#bib145); Stockholm County Council’s Public Health Questionnaire (PHQ)[^156^](#bib156). Studies with multiple observations by geographical scale considering all-cause mortality: Panel Study of Income Dynamics (PSID).[^178^](#bib178)

^c^Studies with multiple observations by adjustments for individual-level variables only or individual and area-level variables considering self-rated health: Canadian National Population Health Survey (CNPHS)[^146^](#bib146); Current Population Survey (CPS)[^184^](#bib184); Comprehensive Survey of Living Conditions of People on Health and Welfare (CSLCPHW)[^157^](#bib157); New European Barometer (NEB); Ontario Health Survey (OHS), an expansion of the National Population Health Survey (NPHS)[^165^](#bib165); Stockholm County Council’s Public Health Questionnaire (PHQ)[^156^](#bib156); World Values Survey (WVS).[^155^](#bib155) Studies with multiple observations by adjustments considering all-cause mortality: Canadian Census Mortality Follow-up Study (CCMFS); Costa Rican Longitudinal Mortality Study (CR-LMS)[^174^](#bib174); Linnaeus Database[^175^](#bib175); US National Longitudinal Mortality Study (NLMS).[^168^](#bib168)

^d^Studies with multiple observations by time lag considering self-rated health: China Health and Nutrition Survey (CHNS)[^183^](#bib183); Chinese Longitudinal Healthy Longevity Survey (CLHLS)[^140^](#bib140); Canadian National Population Health Survey (CNPHS)[^181^](#bib181); Current Population Survey (CPS)[^159^](#bib159); Life Conditions Survey (LCS).[^150^](#bib150) Studies with multiple observations by time lag considering all-cause mortality: Linnaeus Database[^175^](#bib175); National Health Interview Survey (NHIS)[^179^](#bib179); Norway census.[^173^](#bib173)

^e^The analysis includes estimates from Daly 1998[^189^](#bib189) (which does not address cluster), replacing the estimates reported by Zhao, et al., 2021[^178^](#bib178). These reports[^178^](#bib178)^,^ [^189^](#bib189) draw on an overlapping sample from the Panel Study of Income Dynamics (PSID). The analysis also includes estimates from Kahn et al., 1999[^192^](#bib192) which does not address clustering and draws on a sample from the Cancer Prevention Study-II (CPS-II).

^f^The analysis includes estimates reported by Henriksson et al., 2006[^192^](#bib192) which replaces the estimates reported by Henriksson et al., 2007.[^171^](#bib171) In Henriksson et al., 2006[^192^](#bib192), income inequality is measured using the 90/10 ratio). These reports[^178^](#bib178)^,^[^189^](#bib189) draw on an overlapping sample from the Swedish census with Henriksson et al., 2007[^171^](#bib171) considering the effects of income inequality (measured by the Gini coefficient) on a sample stratified by occupation.

^g^The analysis includes estimates reported by Grönqvist et al., 2012[^191^](#bib191) which is a natural experimental study exploiting a refugee resettlement policy, replacing the estimates reported in Henriksson et al., 2007.[^171^](#bib171) The sample of refugees analysed in Grönqvist et al., 2012[^191^](#bib191) is a subset of the Swedish census sample described in Henriksson et al., 2007.[^171^](#bib171)

Appendix 22 Funnel Plot Exploring Publication Bias Among Studies Considering Self-Rated Health


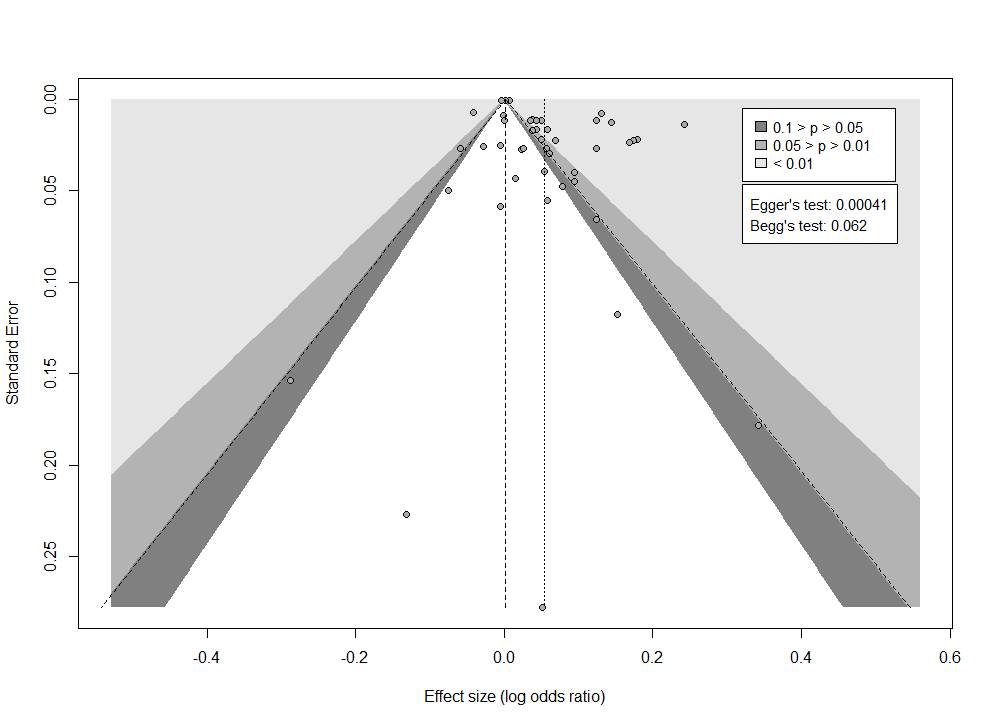


Appendix 23 Funnel Plot Exploring Publication Bias Among Studies Considering All-Cause Mortality


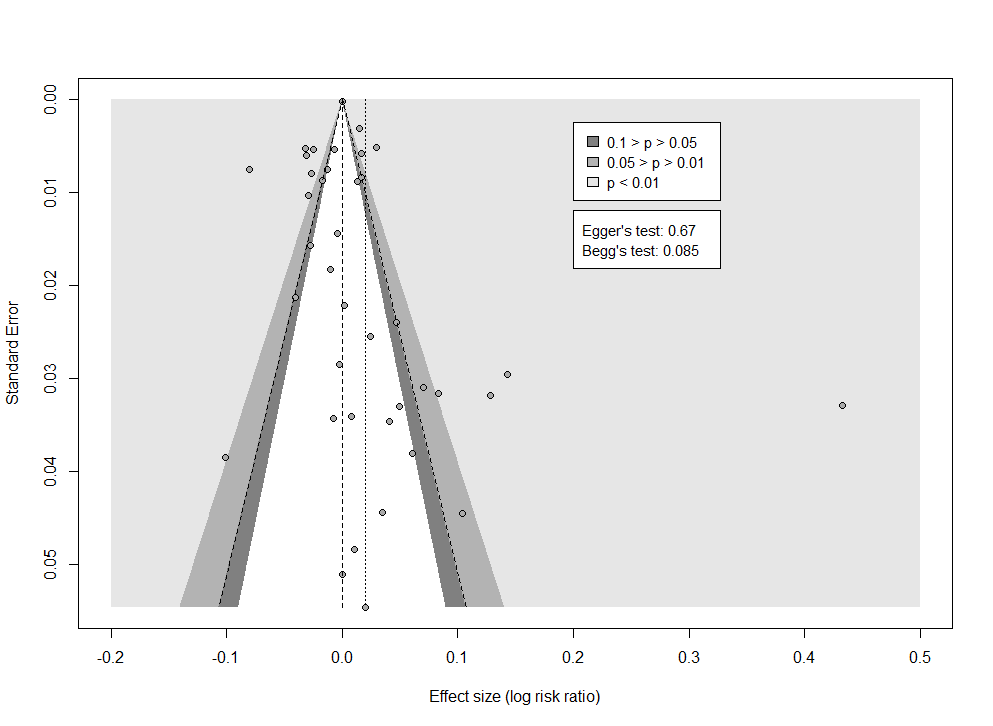

Supplement: Supplementary file 1 — Online Appendix [file MILQ-102-141-s001.docx]
